# Weight trajectories and obesity onset between 17 and 60 years of age, and cause-specific mortality: the Obesity and Disease Development Sweden (ODDS) pooled cohort study

**DOI:** 10.1016/j.eclinm.2026.103870

**Published:** 2026-04-10

**Authors:** Huyen T. Le, Marisa da Silva, Louise Bennet, Ahmed Elhakeem, Christel Häggström, Ming Sun, Innocent B. Mboya, Jens Wahlström, Karl Michaëlsson, Sven Sandin, Patrik K.E. Magnusson, Ylva Trolle Lagerros, Abbas Chabok, Lena Lönnberg, Sölve Elmståhl, Karolin Isaksson, Sara Hägg, Bright I. Nwaru, Hannu Kankaanranta, Linnea Hedman, Anton Nilsson, Josef Fritz, Tanja Stocks

**Affiliations:** a Department of Translational Medicine, Lund University, Malmö, Sweden; b School of Information Technology, Halmstad University, Halmstad, Sweden; c Department of Clinical Sciences in Malmö, Lund University, Malmö, Sweden; d Population Health Sciences, Bristol Medical School, University of Bristol, Bristol, UK; e MRC Integrative Epidemiology Unit, University of Bristol, Bristol, UK; f Northern Registry Centre, Department of Diagnostics and Intervention, Oncology, Umeå University, Umeå, Sweden; g Department of Pharmacy, Xuanwu Hospital of Capital Medical University, Beijing, People's Republic of China; h Africa Academy for Public Health, Dar es Salaam, Tanzania; i Department of Epidemiology and Biostatistics, School of Public Health, KCMC University, Moshi, Tanzania; j Department of Epidemiology and Global Health, Umeå University, Umeå, Sweden; k Medical Epidemiology, Department of Surgical Sciences, Uppsala University, Uppsala, Sweden; l Department of Medical Epidemiology and Biostatistics, Karolinska Institutet, Stockholm, Sweden; m Department of Psychiatry, Icahn School of Medicine at Mount Sinai, New York, USA; n Department of Medicine, Huddinge, Karolinska Institutet, Stockholm, Sweden; o Department of Clinical Sciences, Division of Surgery, Danderyd Hospital, Karolinska Institutet, Stockholm, Sweden; p Centre for Clinical Research Västmanland, Uppsala University, Västerås, Sweden; q Department of Clinical Sciences, Surgery, Lund University, Lund, Sweden; r Department of Surgery, Skåne University Hospital, Kristianstad, Sweden; s Krefting Research Centre, Department of Internal Medicine and Clinical Nutrition, Institute of Medicine, Sahlgrenska Academy, University of Gothenburg, Gothenburg, Sweden; t Wallenberg Centre for Molecular and Translational Medicine, University of Gothenburg, Gothenburg, Sweden; u Department of Respiratory Medicine, Seinäjoki Central Hospital, Wellbeing Services County of South Ostrobothnia, Seinäjoki, Finland; v Faculty of Medicine and Health Technology, University of Tampere, Tampere, Finland; w Department of Public Health and Clinical Medicine, The OLIN and Sunderby Research Unit, Umeå University, Umeå, Sweden; x Institute of Clinical Epidemiology, Public Health, Health Economics, Medical Statistics and Informatics, Medical University of Innsbruck, Innsbruck, Austria

**Keywords:** Obesity, Weight trajectories, Mortality, Pooled cohort study

## Abstract

**Background:**

Longitudinal data on weight change, its timing, and the age of obesity onset in relation to cause-specific mortality are limited.

**Methods:**

From ODDS, a nationwide pooled cohort study in Sweden, we included 258,269 men and 361,784 women with at least three weight assessments between ages 17 and 60, collected in 1963–2015. We applied linear mixed-effects models to estimate weight trajectories, age of obesity onset, and age-specific weight changes between ages 17 and 60. Outcomes were all-cause and cause-specific mortality, assessed from 5 years after the last weight assessment until death, emigration, or 31 December 2020. Associations with mortality were calculated using multivariable Cox regression models.

**Findings:**

Over a median follow-up of 23.3 years in men and 11.7 years in women, 86,673 men and 29,076 women died. The median weight change between ages 17 and 60 was 0.42 kg/year in both sexes. A steep weight gain trajectory over this period, early obesity onset, and high weight gain between ages 17 and 29 were associated with higher all-cause mortality and with 13 of 23 specific causes of death investigated in men and 12 of 19 in women. Affected causes included cardiovascular diseases (including most subtypes), cancer (including specific types), type 2 diabetes, and digestive and genitourinary diseases. Hazard ratios (95% confidence intervals) of all-cause mortality associated with obesity onset at ages 17–29 vs. never by age 60 were 1.69 (1.60–1.79) in men and 1.71 (1.55–1.88) in women; and per 0.5 kg/year weight change at ages 17–29, 1.18 (1.17–1.19) and 1.16 (1.14–1.18), respectively. Weight gain later in adulthood generally showed weaker associations, except for cancer mortality in women, where the association was similar to that observed with earlier weight gain.

**Interpretation:**

Weight gain in adulthood, especially in young adulthood, and obesity onset before age 30 are strong risk factors for mortality from multiple non-communicable diseases, underscoring the importance of early obesity prevention. Future studies should incorporate richer confounding data and, ideally, measures of changes in central adiposity and muscle mass.

**Funding:**

The 10.13039/501100004359Swedish Research Council, 10.13039/501100002794Swedish Cancer Society, Crafoord Foundation, Malmö General Hospital Cancer Foundation, and the Swedish Foundation for Strategic Research.

**Research in context**

**Evidence before this study**

We searched PubMed and Embase for prospective epidemiological studies and meta-analyses on the association between weight or body mass index (BMI) trajectories or changes, and mortality, from database inception until May 31, 2025. Search terms included (“weight change” OR “weight gain” OR “weight trajector∗” OR “BMI trajector∗”) AND (“mortality” OR “death”), limited to studies of adults in the general population. We identified a systematic review and meta-analysis examining weight or BMI change between two points in midlife (ages 40–65) in relation to all-cause, cardiovascular disease, and cancer mortality, as well as a few subsequent studies on similar topics. However, very few studies have used multiple weight assessments across adulthood to explore longitudinal, non-linear weight trajectories in relation to all-cause and cause-specific mortality. While existing evidence suggests that adult weight gain is positively associated with mortality from major non-communicable diseases, no previous study has combined a large population with repeated weight measurements across early to late adulthood, along with a large number and wide range of cause-specific mortality outcomes. The timing of weight gain and obesity onset has also not been well studied in this longitudinal context.

**Added value of this study**

This study of a large Swedish population incorporated all of the above features, providing a more comprehensive understanding of the relationship between adult weight trajectories and mortality than was previously available. The observed weight gain patterns were similar to those in other Western populations, suggesting generalisability of the findings. Greater weight gain across adulthood, particularly during early adulthood (ages 17–29), and earlier obesity onset were associated with increased mortality from all causes and from 13 of 23 specific causes in men and 12 of 19 in women, including many non-communicable diseases. Among women, weight gain at any age was similarly associated with an increased cancer mortality.

**Implications of all the available evidence**

Together with existing evidence, these findings emphasise the importance of prevention strategies that target weight gain early in adulthood. The association between later-life weight gain and cancer mortality in women also suggests a potentially sensitive period around the menopausal transition that may be relevant for cancer prevention. To enable more precise risk estimates for weight gain patterns and health outcomes, future studies should incorporate more detailed covariate information. To better assess the public health and economic burden of adult weight gain, population-level data on contemporary weight gain patterns are also needed.

## Introduction

Obesity, now affecting one in eight individuals globally, is a major contributor to many health issues and a well-established risk factor for premature death.^1^ Studies on cause-specific mortality have typically relied on once-measured body mass index (BMI),^2^, ^3^, ^4^, ^5^ which does not capture dynamic changes in body weight over time. While weight change between two time points in adulthood has been thoroughly examined for some major causes of death, generally showing positive associations,^6^, ^7^, ^8^, ^9^ assessing non-linear patterns and lifelong weight change requires more frequent weight measurements.^10^ A few studies have explored non-linear longitudinal BMI trajectories and found that stable normal weight or slow weight gain was associated with the lowest risk of all-cause mortality and common death causes, such as cardiovascular disease (CVD) and cancer.^11^^,^^12^ Comprehensive investigations into the associations between body weight trajectories and specific causes of death are lacking.

In Western populations, body weight generally increases until late adulthood,^13^^,^^14^ with somewhat different patterns observed between men and women.^14^ In men, weight gain tends to be steeper in young adulthood, whereas in women, it occurs at a more stable rate across adulthood. Also, weight gain at different life stages reflects distinct changes in body composition. In early adulthood, it typically involves an increase in both fat and muscle mass, whereas in later adulthood, it primarily involves fat mass accumulation.^15^ While weight change across different ages and between men and women may have varying associations with mortality, few studies have explored these relationships.^9^^,^^16^ Previous research on weight change in older adults did not adjust for earlier weight changes, making it difficult to separate the effects of weight gain in later life from those in earlier years. Additionally, investigating the age of obesity onset alongside age-specific weight changes could offer valuable insights into whether prolonged exposure to excess weight, or the timing of weight gain, is the primary driver of increased mortality risk. However, studies that address this issue are scarce.^16^

In a pooled Swedish cohort of individuals with at least three weight assessments between age 17 and 60, we aimed to investigate (1) weight trajectories throughout adulthood, (2) the age of obesity onset, and (3) weight changes during different age periods in relation to all-cause and cause-specific mortality.

## Methods

### Study population

We used data from the Obesity and Disease Development Sweden (ODDS) study, described in detail elsewhere.^17^ Briefly, the ODDS study was established to investigate the association between adult body size measures and morbidity and mortality. Body size assessments from age 17 onwards as well as height and smoking information were pooled from multiple cohorts across Sweden. Sociodemographic information was retrieved from national registers using the unique national personal identity number. Information on date of birth, country of birth, and marital status were retrieved from the Total Population Register. Information on highest attained education level was retrieved from the Longitudinal Integrated Database for Health Insurance and Labour Market Studies (LISA) (since 1990) and the Population and Housing Censuses (for the period 1960–1990). In total, the ODDS study includes 4,295,859 individuals with 7,733,901 weight assessments recorded between ages 17 and 103, collected from 1963 to 2020; the earliest recalled weights pertain to historical data from 1911.

**Fig. 1:**
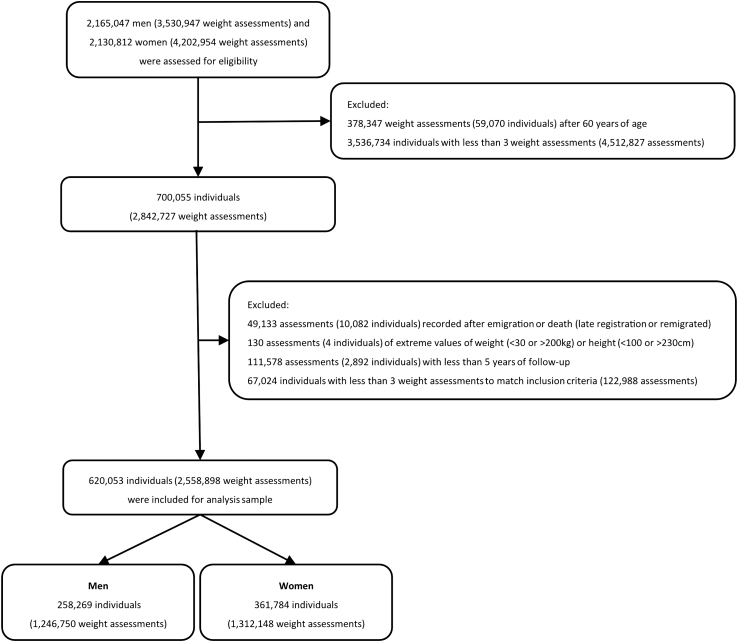
**Flowchart of the study population**. When exclusions were based on the number of individuals (with the number of assessments shown in brackets), the entire individual and all their assessments were excluded. In contrast, when exclusions were based on weight assessments (with the number of individuals shown in brackets), only those specific assessments were excluded, while remaining eligible assessments for the same individuals were retained for subsequent steps.

This study included 258,269 men (1,246,750 assessments) and 361,784 women (1,312,148 assessments) with at least three weight assessments between ages 17 and 60, after exclusions for mismatched dates, extreme height (<100 or >230 cm) or weight (<30 or >200 kg), or less than 5 years of follow-up to reduce the potential influence of prevalent disease on weight (Fig. 1). The Construction Workers Cohort (CWC)^18^ made up 67% of weight assessments in men, and the Medical Birth Register (MBR)^19^ made up 60% of assessments in women. These cohorts are both nationwide and collected objectively measured weights; in the MBR, weight was recorded around gestational week 8–10 of pregnancy, before any appreciable pregnancy-related weight gain has occurred.^20^ Details of the study cohorts, including the number and years of weight assessments, are provided in Table S1. Overall, 74% of weight assessments were objectively measured, 10% were self-reported current weight, and 16% were self-reported recalled weight.

### Ethics

The study was approved by the Swedish Ethical Review Authority (no: 2020-03846). Some of the included cohorts collected informed consent from participants, and others did not. The two largest cohorts in ODDS include individuals in national registers (military conscripts and women giving birth) from whom informed consent was not collected. However, the requirement for informed consent was waived by the Swedish Ethical Review Authority due to the reuse of pseudonymised data from existing cohorts, the absence of access to re-identification keys by the research team, and the register-based nature of the study.

### Follow-up

The unique national personal identity number of all Swedish inhabitants was used to follow individuals for emigration in the Total Population Register, and for date and underlying cause of death in the Cause of Death Register,^21^ until 31 December 2020. Main outcomes included all-cause mortality, all-cause mortality excluding respiratory diseases and lung cancer (due to potential residual confounding by smoking), CVD mortality, cancer mortality, and obesity-related cancer mortality. Cause-specific mortality was further analysed for obesity-related death causes, provided the number of events exceeded 250. The selection of obesity-related death causes is described in the Appendix p 5, with the categorisation and number of deaths in Table S2. The categorisation was guided by the International Classification of Diseases, in which, for example, in the 10th Revision (ICD-10), hypertension, but not type 2 diabetes mellitus (T2DM), is classified under Chapter I: Diseases of the circulatory system (CVDs).

### Statistical analysis

The following exposures were investigated separately for men and women in relation to mortality: weight trajectories between ages 17 and 60, categorised into quintiles (Aim 1); age of obesity onset, categorised as 17–29, 30–44, 45–60 years, or never developing obesity by age 60 (Aim 2); and weight changes during the age periods 17–29, 30–44 and 45–60, per 0.5 kg/year increase (Aim 3). These age periods spanned around 15 years, and weight changes were approximately linear within each period. We used a two-stage approach to analyse longitudinal and survival data.

In the first stage, we used linear mixed-effects (LME) models to estimate the exposures.^22^ These models accounted for the hierarchical structure of the data, with repeated weight measurements nested within individuals. We estimated individual weight trajectories between age 17 and 60, using an LME model with fixed effects including natural cubic splines of age (four knots, following Harrell's recommendation),^23^ mode of weight assessment (measured, self-reported, recalled) and, for women, pregnancy status at weight assessment (assessment from the MBR, yes/no). Random effects included an individual-specific random intercept and individual-specific random slope for age, which were estimated via best linear unbiased prediction (BLUP). We used the random slope (i.e. how the individual-level trajectory deviated from the population-level trajectory) to categorise individual weight trajectories into sex-specific quintiles (Aim 1). The weight trajectories were modelled from all observed weight measurements prior to the start of follow-up. BMI for ages 17 to 60 was calculated using predicted weight (kg) divided by measured height (m) squared. The age of obesity onset was defined as the first age with a predicted BMI ≥30 kg/m^2^ (Aim 2). Weight changes in age periods were calculated using LME models with fixed effects including linear splines of age (two knots at ages 30 and 45), mode of weight assessment, and pregnancy status at weight assessment (for women). Random effects included an individual-specific random intercept and individual-specific random slopes for age within each of the three age ranges described. Individual-specific weight changes (slope) for each age period were derived by combining the fixed and random slopes for the corresponding period (Aim 3). The rationale for the LME models is provided in the Appendix p 5.

In the second stage, we used Cox regression with age as timescale to estimate hazard ratios (HRs) of mortality outcomes. Follow-up started 5 years after the last weight assessment and continued until emigration, death, or the end of follow-up, whichever came first. The 5-year lag was chosen to align with our selection criterion of excluding weight assessments with less than 5 years of follow-up, thereby avoiding immortal time. The Cox regression models were stratified by decades of birth cohort and adjusted for potential confounders: predicted weight at age 17 (continuous), height (continuous; not adjusted for in the obesity onset analysis), and categories as in Table 1 for country of birth, highest attained education, and marital status and current smoking at the last weight assessment. In the analysis of weight changes in different age periods, we additionally adjusted for weight changes from previous age periods. Among the co-variables, current smoking had the highest proportion of missingness: 1.8% in men and 14.5% in women, while other co-variables (height, education level, birth country, and marital status) had less than 1% missing data. Missing values for co-variables were imputed using multiple imputation by chained equations (MICE), under the assumption of missing at random (MAR) (details in the Appendix pp 5 and 6). We also graphically examined the shape of the association between weight changes in age periods and the main outcomes using restricted cubic splines (four knots), with the same strata and adjustments as described earlier. An evaluation of the proportional hazards assumption using Schoenfeld residuals over analysis time showed no major violations. Age-standardised mortality rates were calculated using the age distribution of men and women in Sweden in 2020.^24^

**Table 1:** Characteristics of men and women, respectively, in quintile one or five of weight trajectories (for Aim 1), or with obesity onset between age 17 and 29 or never by age 60 (for Aim 2).

Characteristic^a^	Men (Full population N = 258,269)				Women (Full population N = 361,784)			
	Weight trajectories		Age of obesity onset^b^		Weight trajectories		Age of obesity onset^b^	
	Quintile 1 (N = 51,654)	Quintile 5 (N = 51,653)	Ages 17–29 (N = 4884)	Never by age 60 (N = 209,120)	Quintile 1 (N = 72,357)	Quintile 5 (N = 72,356)	Ages 17–29 (N = 20,534)	Never by age 60 (N = 256,086)
Age at last obs (yrs), median (IQR)	54.4 (44.5–59.7)	44.4 (33.8–52.8)	40.2 (29.4–50.6)	49.8 (37.0–58.0)	47.5 (36.6–57.5)	35.2 (31.2–40.8)	32.5 (29.2–36.5)	38.5 (33.0–52.1)
Calendar year of first obs, median (IQR)	1972 (1962–1975)	1974 (1972–1978)	1975 (1972–1981)	1973 (1971–1976)	1981 (1964–1995)	1994 (1984–2001)	1998 (1992–2004)	1988 (1973–1999)
Years from first to last obs, median (IQR)	17.8 (11.4–34.0)	16.7 (11.0–29.1)	14.0 (8.4–21.8)	15.6 (9.0–30.0)	19.9 (10.0–33.8)	10.5 (7.0–17.1)	8.5 (5.9–12.4)	11.6 (7.0–27.7)
Calendar year of birth, median (IQR)	1939 (1927–1949)	1951 (1943–1958)	1953 (1943–1962)	1945 (1933–1954)	1955 (1943–1968)	1969 (1960–1976)	1974 (1968–1980)	1962 (1949–1973)
Weight at last obs (kg), mean (SD)	72.1 (8.7)	92.6 (12.3)	106.7 (15.7)	77.4 (8.7)	58.5 (7.4)	87.0 (12.9)	97.2 (14.8)	63.5 (7.6)
Weight change, 17–60 yrs (kg/yr), median (IQR)	0.16 (0.09–0.21)	0.73 (0.66–0.85)	0.62 (0.36–0.90)	0.38 (0.25–0.49)	0.17 (0.10–0.22)	0.78 (0.69–0.94)	0.87 (0.67–1.10)	0.34 (0.24–0.44)
Height (cm), mean (SD)	175.8 (6.6)	180.0 (6.4)	177.4 (7.2)	177.9 (6.4)	163.9 (5.9)	167.1 (6.2)	165.0 (6.6)	165.9 (6.1)
BMI at last obs (kg/m^2^), mean (SD)	23.3 (2.5)	28.6 (3.6)	33.9 (4.3)	24.5 (2.3)	21.8 (2.4)	31.2 (4.6)	35.7 (4.7)	23.1 (2.5)
BMI at last obs in categories, n (%)^b^								
Underweight	666 (1.3)	4 (<0.1)	1 (<0.1)	1054 (0.5)	4234 (5.9)	3 (<0.1)	5 (<0.1)	5284 (2.1)
Normal weight	40,332 (78.1)	7642 (14.8)	43 (0.9)	122,204 (58.5)	61,887 (85.5)	3244 (4.5)	184 (0.9)	198,550 (77.5)
Overweight	10,085 (19.5)	28,218 (54.6)	523 (10.7)	84,764 (40.5)	5712 (7.9)	29,251 (40.4)	1206 (5.9)	51,329 (20.0)
Obesity	571 (1.1)	15,789 (30.6)	4317 (88.4)	1097 (0.5)	524 (0.7)	39,859 (55.1)	19,138 (93.2)	923 (0.4)
Current smoker at last obs, n (%)								
No	33,532 (64.9)	37,280 (72.2)	3394 (69.5]	142,628 (68.2)	56,651 (78.3)	58,586 (81.0)	15,829 (77.1)	210,141 (82.1)
Yes	18,122 (35.1)	14,373 (27.8)	1490 (30.5)	66,491 (31.8)	15,706 (21.7)	13,770 (19.0)	4704 (22.9)	45,945 (17.9)
Highest achieved education, n (%)								
Pre-upper secondary school <9 yrs	19,681 (38.1)	10,059 (19.5)	1073 (22.0)	61,552 (29.4)	8597 (11.9)	4816 (6.7)	1267 (6.2)	20,155 (7.9)
Pre-upper secondary school 9 yrs	3644 (7.0)	5805 (11.2)	554 (11.3)	17,435 (8.3)	5498 (7.6)	7476 (10.3)	2444 (11.9)	18,229 (7.1)
Upper secondary school <3 yrs	15,128 (29.3)	22,148 (42.9)	2273 (46.5)	73,315 (35.1)	19,176 (26.5)	20,940 (28.9)	5558 (27.1)	64,177 (25.0)
Upper secondary school 3 yrs	6601 (12.8)	6841 (13.2)	511 (10.5)	28,911 (13.8)	9301 (12.8)	15,792 (21.8)	5755 (28.0)	40,112 (15.7)
Post-upper secondary school ≥1 yrs	6600 (12.8)	6800 (13.2)	473 (9.7)	27,906 (13.4)	29,785 (41.2)	23,332 (32.3)	5509 (26.8)	113,413 (44.3)
Birth country, n (%)								
Born in SE and both parents born in SE	47,542 (92.0)	46,824 (90.7)	4375 (89.6)	191,583 (91.6)	60,657 (83.8)	51,990 (71.8)	14,318 (69.7)	207,623 (81.1)
Born in SE and one/both parents born abroad	1375 (2.7)	2649 (5.1)	306 (6.3)	7872 (3.8)	4599 (6.4)	6710 (9.3)	2241 (10.9)	19,596 (7.6)
Born abroad	2737 (5.3)	2180 (4.2)	203 (4.1)	9664 (4.6)	7101 (9.8)	13,656 (18.9)	3974 (19.4)	28,867 (11.3)
Marital status at last obs, n (%)								
Unmarried	9336 (18.1)	16,283 (31.5)	2220 (45.4)	52,254 (25.0)	13,315 (18.4)	19,103 (26.4)	6332 (30.8)	55,558 (21.7)
Married/registered partner	36,431 (70.5)	30,370 (58.8)	2323 (47.6)	136,025 (65.0)	46,566 (64.4)	44,095 (60.9)	11,873 (57.8)	166,783 (65.1)
Divorced or widow/-er from spouse/partner	5887 (11.4)	5000 (9.7)	341 (7.0)	20,841 (10.0)	12,476 (17.2)	9158 (12.7)	2328 (11.4)	33,745 (13.2)

Abbreviations: obs, observation; yr, year; BMI, body mass index; IQR, interquartile range; SD, standard deviation; SE, Sweden.

a Characteristics after multiple imputation of missing values.

b The age of obesity onset was defined based on the predicted BMI (predicted weight from a linear mixed-effects model divided by measured height in kg/m^2^). As a result, there is a slight mismatch between the age-of-onset groups and the observed BMI categories at the last observation.

We conducted several additional analyses. For weight changes in age periods, we performed analyses that i) excluded sex-specific cancers from obesity-related cancer deaths, ii) excluded individuals losing (predicted) weight, and iii) used age-specific weight change per standard deviation (SD)/year increase instead of per 0.5 kg/year as exposure. For all exposures, we performed analyses that i) included individuals with at least two weight assessments in addition to those with at least three weight assessments, ii) included only individuals with at least one weight assessment per age period, 17–29, 30–44 and 45–60 years, iii) were unadjusted for smoking, iv) used BMI at the last assessment as exposure, and v) used a negative control outcome, brain cancer mortality. Post-hoc analyses included i) applying the E-value method to evaluate the robustness of observed associations with respect to unmeasured confounding, and ii) performing complete-case analyses and comparing the results with those obtained using multiple imputation to evaluate the robustness of the imputation model. The rationale for and further details of the additional analyses are described in the Appendix pp 6–8.

All analyses were conducted using Stata/MP version 18.0 (StataCorp LLC, College Station, Texas), with two-sided tests and a significance threshold of <0.05.

### Role of the funding source

The funders of the study had no role in study design, data collection, data analysis, data interpretation, writing of the paper, or decision to submit the paper for publication.

## Results

### Population characteristics

**Fig. 2:**
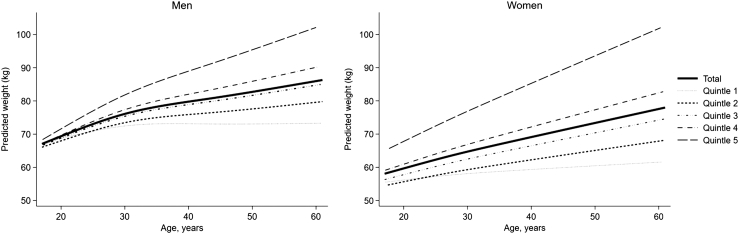
**Weight trajectories between 17 and 60 years of age in men (left) and women (right) in total and in quintiles**. The weight trajectories were estimated using a linear mixed-effects model of weight with natural cubic splines of age (four knots). Other predictors in the linear mixed-effects model were the mode of weight measurement (measured, self-reported, recalled) and pregnancy status at the time of weight assessment (yes, no) (for women). In men, the median weight gain between ages 17 and 60 years was 7.0, 13.5, 18.1, 22.8, and 31.4 kg for quintiles 1–5, respectively, corresponding to 7.3, 13.4, 17.9, 23.2 and 33.7 kg in women.

The median number of weight assessments (interquartile range [IQR]) per individual was 4 (3–6) in men and 3 (3–4) in women. Weight trajectories in quintiles throughout ages 17–60 are shown in Fig. 2, and population characteristics are shown in Table 1 (partial population) and Table S3a and b (full population). The median weight change throughout ages 17 and 60 was a gain of 18.1 kg in men and 17.8 kg in women, corresponding to an average annual gain of 0.42 kg/year in both sexes; however, weight trajectories over this period differed between men and women. Weight gain decreased from younger to older age periods, especially in men, and the proportion of individuals with obesity onset increased by each age period (Table S4).

Weight assessments were typically concentrated in earlier calendar years for men than for women, resulting in longer follow-up periods in men. During a median (IQR) follow-up of 23.3 years (14.6–26.0) in men and 11.7 years (4.9–17.8) in women, 86,673 men and 29,076 women died. The median (IQR) age at death was 77.0 years (68.5–84.0) in men and 78.4 years (68.0–86.7) in women.

### Summary of key findings

**Fig. 3:**
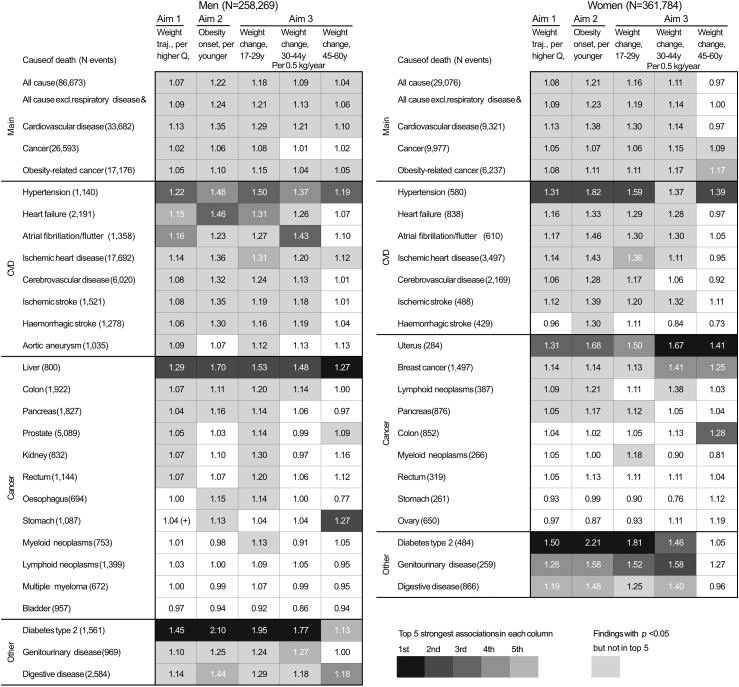
**Summary findings of adulthood weight change and obesity metrics in relation to all-cause and cause-specific mortality in men (left) and women (right)**. Black and grey-shaded cells indicate positive associations with a p-value below 0.05, with a gradually darker shade indicating the top five strongest associations in each analysis, as represented by columns. These were judged based on hazard ratios (as indicated within cells) of mortality from the causes listed in the left panel for i) per higher quintile of weight trajectories at ages 17–60 years (*Aim 1.* “+” denotes an association for quintile 5 vs. 1 but not per higher quintile), ii) per younger age category of obesity onset, i.e., ages 17–29, 30–44, 45–60 years, or never developing obesity by age 60 years (*Aim 2*), and iii) per 0.5 kg/year weight change at ages 17–29, 30–44 and 45–60 (*Aim 3*). Excl.: excluding, CVD: cardiovascular disease, traj.: trajectory, Q: quintile, cat.: category, y: years. Mortality from aortic aneurysm and from cancer of the oesophagus, liver, kidney, and bladder, and multiple myeloma, were not analysed in women due to a small number of events (<250). Details on the models used for each analysis are shown in Fig. 4 for Aim 1, Fig. 5 for Aim 2, and Fig. 6 for Aim 3.

Fig. 3 summarises the findings from the investigated aims. Steeper upward weight trajectories between ages 17 and 60, obesity onset at younger ages, and higher weight gain at ages 17–29 were all significantly associated with higher risks of all five main mortality outcomes, 13 of 23 specific death causes in men, and 12 of 19 specific death causes in women. Associations with all-cause mortality were stronger after excluding respiratory diseases and lung cancer, and associations with cancer were stronger for obesity-related cancers only. Among individual causes of death, associations were generally the strongest for T2DM, but were also strong for hypertension, liver cancer (in men), and uterine cancer.

Mortality from bladder cancer, lymphoid neoplasms and multiple myeloma in men, and from rectal, stomach and ovarian cancer in women, showed no significant associations with any exposure (Aims 1–3).

### Weight trajectories between ages 17 and 60, and mortality (Aim 1)

**Fig. 4:**
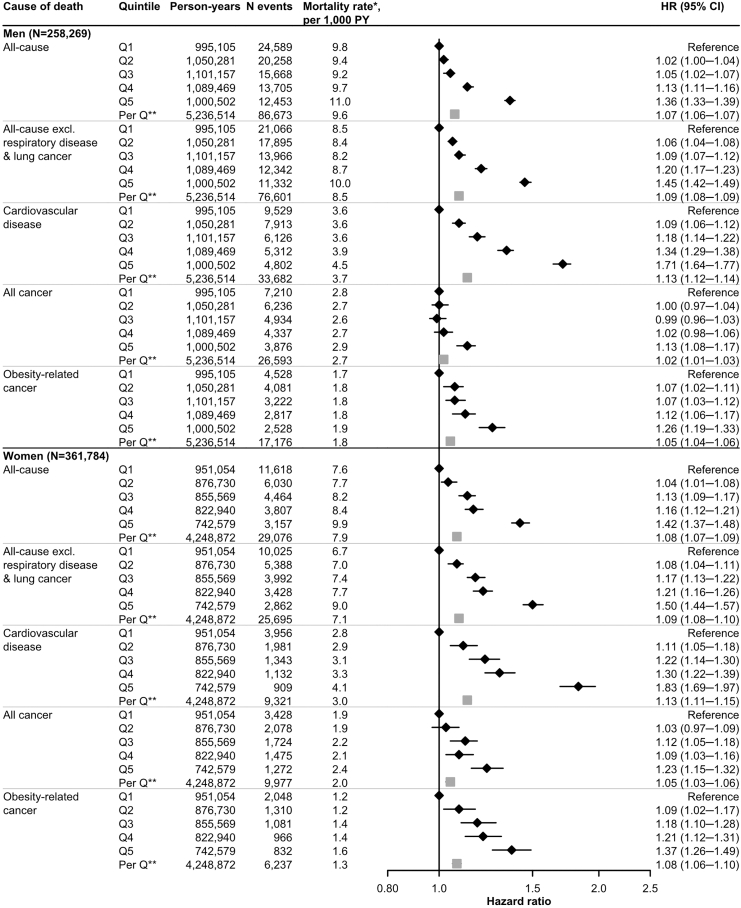
**Hazard ratios (95% confidence intervals) of mortality from all causes, all causes excluding respiratory disease and lung cancer, all cancer, obesity-related cancer, and cardiovascular disease, according to weight trajectories at ages 17–60 (Aim 1), according to quintiles in men and women, respectively**. The weight trajectories were estimated using a linear mixed-effects model of weight with natural cubic splines of age (four knots). Other predictors in the linear mixed-effects model were the mode of weight measurement (measured, self-reported, recalled) and pregnancy status at the time of weight assessment (yes, no) (for women). Multivariable Cox regression with age as timescale was used to estimate the HRs and 95% CIs. The HRs were adjusted for predicted weight at age 17, height, highest attained education, birth country, marital status and current smoking at the last weight assessment, and stratified by birth decade. The HR per quintile was derived from a Cox model treating quintiles of weight trajectories as a continuous variable, adjusted for the same variables as above. ∗Mortality rates were age-standardised to the Swedish population in 2020. ∗∗Person-years, N events, and mortality rates per quintile regards the value across all quintiles. PY, person-years; HR, hazard ratio; CI, confidence interval; excl., excluding; Q, quintile.

Fig. 4 shows the associations to progressively higher risks of main mortality outcomes across increasing quintiles of weight trajectories between ages 17 and 60. This pattern was observed for all investigated non-cancer death causes, except haemorrhagic stroke in women, and for several cancers (Figure S1a and b).

### Age of obesity onset and mortality (Aim 2)

**Fig. 5:**
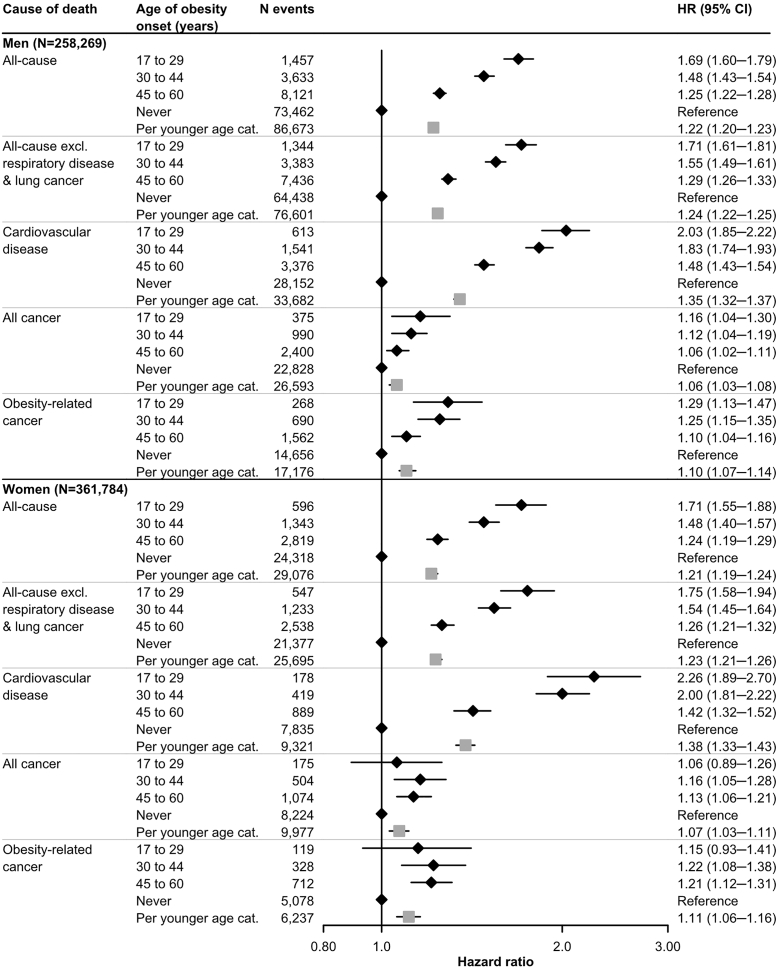
**Hazard ratios (95% confidence intervals) of mortality from all causes, all causes excluding respiratory disease and lung cancer, all cancer, obesity-related cancer, and cardiovascular disease according to age of obesity onset in men and women (Aim 2), respectively**. The number at risk for age of obesity onset at 17–29, 30–44, 45–60 years of age, and never were 4,885, 13,570, 30,695 and 209,119 in men; and 20,534, 34,103, 51,059 and 256,088 in women∗. Predicted individual weight within ages 17–60 years was derived from a linear mixed-effects model of weight using natural cubic splines of age (four knots). Other predictors in the linear mixed-effects model were the mode of weight measurement (measured, self-reported, recalled) and pregnancy status at the time of weight assessment (yes, no) (for women). Individual BMI at any age within 17–60 years was calculated as predicted individual weight at each age divided by measured height (kg/m^2^). Age at the first time with a predicted BMI ≥30 kg/m^2^ was treated as the age of obesity onset and used to classify into four groups, i.e. obesity onset at 17–29, 30–44, 45–60 and never developing obesity within ages 17–60 years (called never in the forest plot). Multivariable Cox regression with age as timescale was used to estimate the HRs and 95% CIs. The HRs were adjusted for predicted weight at age 17, highest attained education, birth country, marital status and current smoking at the last weight assessment, and stratified by birth decade. The HR per younger age category was derived from a Cox model including the categorical variable as a continuous variable, adjusted for the same variables as above. HR: hazard ratio, CI: confidence interval, cat.: category, excl.: excluding. ∗The number at risk and number of events were taken from imputed dataset 1, but varies slightly between imputed datasets.

Obesity onset at younger age was associated with higher mortality from all non-cancer causes of death, except aortic aneurysm in men, and with mortality from several cancers (Fig. 5 for main mortality outcomes and Figure S2a and b for cause-specific mortality). Compared to individuals never developing obesity by age 60, the HR (95% confidence interval [CI]) of all-cause mortality for obesity onset at ages 17–29 was 1.69 (1.60–1.79) in men and 1.71 (1.55–1.88) in women.

### Weight changes in age periods and mortality (Aim 3)

Weight changes at ages 17–29 were generally positively and linearly associated with all-cause, CVD, and cancer mortality. By contrast, at older ages, the associations were generally J-shaped, with the lowest risk occurring at approximately 0–0.25 kg/year (Figure S3a and b).

**Fig. 6:**
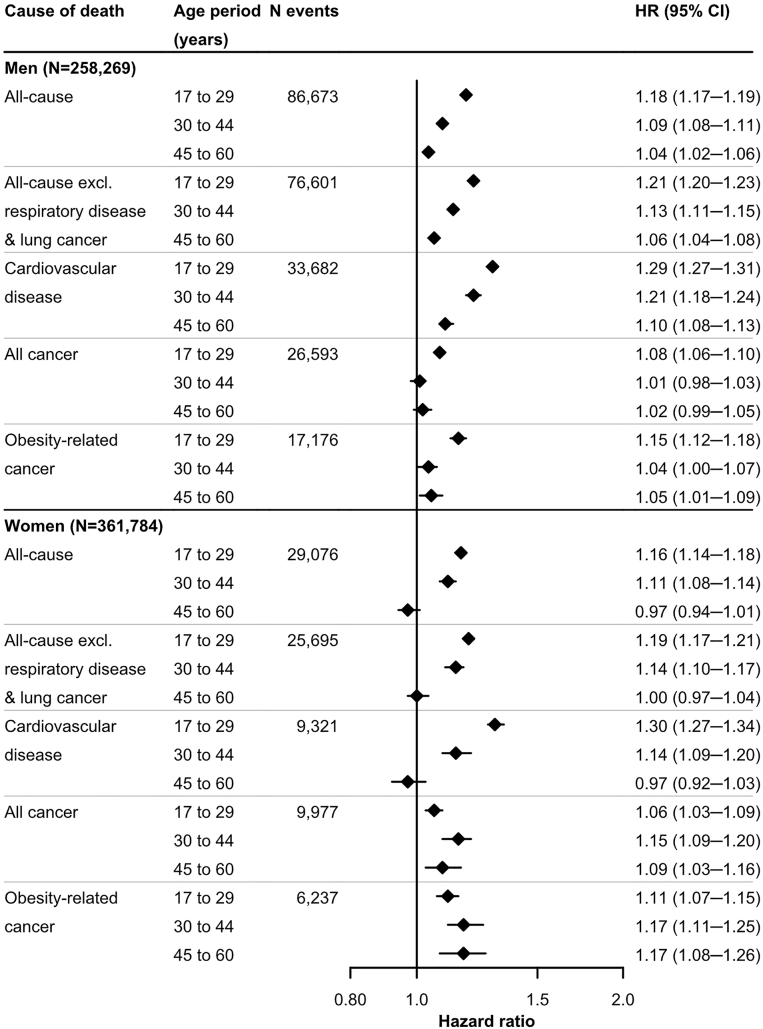
**Hazard ratios (95% confidence intervals) of mortality from all causes, all causes excluding respiratory disease and lung cancer, all cancer, obesity-related cancer, and cardiovascular disease per 0.5 kg/year weight change at ages 17–29, 30–44 and 45–60 years in men and women (Aim 3), respectively**. The individual weight change at ages 17–29, 30–44 and 45–60 years was the individual random slope derived from a linear mixed-effects model of weight using linear splines of age at these periods. Other predictors in the linear mixed-effects model were the mode of weight measurement (measured, self-reported, recalled) and pregnancy status at the time of weight assessment (yes, no) (for women). Multivariable Cox regression with age as timescale was used to estimate the HRs and 95% CIs. The HRs were adjusted for predicted weight at age 17, height, highest attained education, birth country, marital status and current smoking at the last weight assessment, and stratified by birth decade. The age periods 30–44 and 45–60 additionally included adjustment for weight change in the previous age periods. HR, hazard ratio; CI, confidence interval; excl., excluding.

In linear risk models, the HR (95% CI) for all-cause mortality per 0.5 kg/year weight change at ages 17–29 was 1.18 (1.17–1.19) in men and 1.16 (1.14–1.18) in women, and was lower for weight change at older ages (Fig. 6). Similar weaker associations for weight change in older age than in young age were observed for CVD mortality including from most separate causes, and for mortality from T2DM, digestive disease and genitourinary disease (Figure S4a and b). These patterns were also found for cancer mortality and most cancer subtypes in men. In women, however, weight change across age periods showed similar positive associations with cancer mortality, with overlapping CIs for any cancer and most cancer subtypes.

### Additional analyses

The associations between weight change during different age periods and mortality from obesity-related cancers remained consistent after excluding sex-specific cancers (prostate, uterus, ovary, and breast in women), with early adulthood weight gain showing the strongest association to mortality in men, and similar associations observed for weight gain in both early and late adulthood in women (Table S5). Excluding individuals losing weight slightly strengthened associations for mortality outcomes, especially in older ages, but for outcomes most strongly associated with weight change at ages 17–29 in the primary analyses, the strongest associations generally remained within that age (Figure S5a and b). The difference in HRs between the youngest and oldest age periods overall remained similar when weight change was analysed per age-specific 1-SD/year rather than per 0.5 kg/year (Figure S6a and b). Cancer mortality in women was an exception; in this analysis, it showed the strongest positive association with weight change at ages 17–29—similar to the patterns observed in men and for other causes of death, although the difference in effect sizes between the age periods remained small.

Additional analyses conducted for all exposures showed that: i) including individuals with at least two weight assessments showed similar results to the analysis requiring at least three assessments per individual (Table S6), ii) restricting the analyses to individuals with at least one weight assessment per age period showed broadly similar patterns, except for cancer mortality where HR CIs largely overlapped between the three age periods (Table S7), iii) not adjusting for smoking slightly attenuated the associations (Table S8), iv) BMI at the last assessment was positively associated with all death causes, except those weakly or not associated with any investigated exposure (Table S9), and v) brain cancer mortality as negative control showed no association with any investigated exposure (Table S10). Post-hoc analyses showed that: i) the E-value varied by outcome, ranging from 1.5 to 8.6 for HR estimates comparing weight trajectory quintile 5 vs. 1 across mortality outcomes in men, and from 1.8 to 11.1 in women (Table S11), and the estimates from the complete-case analyses were very similar to those obtained using multiple imputation (Table S12).

## Discussion

In this large Swedish cohort, adult weight gain, particularly in young adulthood, and earlier obesity onset were associated with higher mortality. These associations were evident for all-cause mortality and for 13 of 23 specific causes of death in men and 12 of 19 in women. Affected causes included CVD (including most subtypes), cancer (including specific types), T2DM, and digestive and genitourinary diseases. Weight gain in early adulthood showed stronger associations with most outcomes than weight gain later in life. Among women, weight gain at any age was similarly associated with increased cancer mortality.

Consistent with previous studies,^13^^,^^14^ weight increased between ages 17 and 60 in our cohort, with the most rapid gain in early adulthood, especially among men. Individuals with the steepest weight trajectory had an around 40% higher risk of all-cause mortality than those with the flattest trajectory. By comparison, in a U.S. study, individuals with the greatest weight gain between ages 25 and 57 had only a 14% higher risk of mortality than those with stable weight, despite similar differences in absolute weight gain between groups and similar model adjustments to ours.^9^ The weaker association in the U.S. study may reflect exclusion of ages 17–24, reliance on recalled weight at age 25, and use of only two weight assessments to calculate weight changes, increasing susceptibility to nondifferential measurement error and underestimation of the true effect.^25^ Adjustment for physical activity, diet, and general health further attenuated estimates in the U.S. study, highlighting the influence of multiple factors and the need to interpret effect sizes with caution and to focus more on patterns and directions of association. Our finding of weaker associations in later adulthood aligns with other U.S. data showing higher mortality risks with earlier overweight or obesity onset.^9^^,^^16^

The association between weight gain and all-cause mortality was largely driven by CVD, which accounted for 37% of deaths, along with strong associations for mortality from T2DM and, to a lesser extent, genitourinary and digestive diseases. While few studies have examined weight change and T2DM mortality, early obesity onset is a well-established strong risk factor for incident T2DM.^26^ Importantly, using only the underlying cause of death, as in our study, likely underestimates the burden of T2DM, which contributes to other fatal conditions, particularly CVD. Weight gain is a risk factor for CVD mortality,^6^^,^^9^^,^^11^^,^^16^ but specific causes of CVD death have been less investigated. Most specific CVD causes we examined followed the pattern of overall CVD mortality, except haemorrhagic stroke in women and aortic aneurysm in men, which showed weak associations. These exceptions align with our sensitivity analyses of BMI and haemorrhagic stroke mortality in women, and a weaker association between BMI and aortic aneurysm mortality compared to most other CVD deaths in both our data and a previous study.^2^ Our findings of higher all-cause and CVD mortality associated with early weight gain and obesity onset suggest that the duration of obesity, rather than weight gain in late adulthood, may be the key factor underlying risk. Long-term exposure to insulin resistance, inflammation, and hypercoagulation due to adipocytokines released from adipose tissue likely contribute to these risks.^26^

Associations with cancer mortality, accounting for 31% of deaths, varied by cancer site and sex. Consistent with large studies,^7^^,^^12^ we found positive associations between adult weight gain and overall cancer mortality. In men, this was driven by early adulthood weight gain, whereas in women, a broadly similar positive association was observed for weight gain across all ages. The persisting positive association also in the oldest age period (45–60 years) in women may partly reflect the role of sex hormones in female cancers. After menopause, adipose tissue becomes the main source of circulating oestrogen, influencing breast and reproductive cancers,^27^ which could be an important pathway underlying our findings for weight gain during this period and obesity-related cancer mortality. However, this association persisted even after excluding breast and reproductive cancers, suggesting that other pathways may also be involved. These could include insulin, insulin-like growth factors, and systemic inflammation, which are hypothesised to link adiposity with several cancers, including cancer of the liver, colorectum, pancreas, and uterus.^27^

Our study has several strengths. Weight trajectories were estimated from a large number of weight assessments spanning through adulthood, with most weights being objectively measured. The study draws on nationwide cohort data across Sweden. While representativeness is difficult to assess for the full population, it is high for the two cohorts contributing the most weight assessments; attendance rate was over 80% in the CWC, and virtually all women giving birth in Sweden are included in the MBR.^17^ The large sample size and long follow-up enabled robust analysis of multiple specific causes of death, as recorded in a high-quality nationwide register.^21^ Previous simulation results have shown that the two-stage modelling approach we used produces unbiased estimates of how growth trajectories influence longitudinal outcomes.^28^ Support for the validity of our approach was also provided by our negative control analysis with brain cancer as the outcome. Furthermore, the adjustment for prior weight change in age period analyses allowed us to avoid mixing up the effects of weight gain in older age from that in younger age, an approach lacking in previous studies.^9^^,^^16^

Our study also has limitations. First, we lacked data on the intentionality of weight loss. To mitigate effects of prevalent disease, we excluded weight measures with less than 5 years of follow-up. We also conducted sensitivity analyses excluding individuals who lost weight, which somewhat strengthened the associations, especially at older ages when comorbidities are more common. Second, information on several potential confounders was lacking or incomplete. Smoking was adjusted for only as current smoker (yes/no). Removing this adjustment slightly weakened associations for smoking-related mortality, suggesting that adjustment for more detailed smoking data might reveal stronger associations. Potentially important confounders for which we lacked information include physical activity, diet (including alcohol intake), co-morbidities, medications, and female-specific factors (e.g., parity, menopausal status, and hormone therapy usage). However, because some of these variables may have bidirectional relationships with BMI,^29^^,^^30^ adjusting for them could obscure some of the true effect of weight gain by removing mediating pathways. To assess the robustness of our findings to unmeasured confounding, we performed negative control analyses and calculated E-values. No association was observed in the negative control analysis, suggesting that residual confounding or systematic bias is unlikely to largely account for the observed associations. Additionally, the E-values provided a rough indication of the robustness of the associations to unmeasured confounding for specific outcomes. For some outcomes, such as mortality from T2DM and hypertension, the E-values were large, suggesting that unmeasured confounding is less likely to fully explain the observed associations. Third, a limitation of the two-stage modelling approach is that uncertainty from the first-stage estimates is not propagated to the second stage, which may lead to underestimation of standard errors. Alternatives, such as joint modelling or bootstrapping, could address this issue but are currently computationally too intensive for large datasets like ours. Finally, we did not apply corrections for multiple testing to avoid obscuring potentially important findings. This approach increases the risk of false-positive results; therefore, we emphasise patterns of results, biological plausibility, and consistency with prior research. Overall, results should be interpreted cautiously, and causality cannot be inferred. Further studies with richer data on potential confounders are needed to validate our findings. The investigation of other measures than weight, reflecting changes in central adiposity and muscle mass, would further strengthen these studies.

Overall, weight gain across adulthood, particularly during early adulthood, and early obesity onset were associated with increased mortality from a wide range of non-communicable diseases. These findings suggest a duration–response association between obesity and later health outcomes. In women, weight gain in later adulthood was also associated with increased cancer mortality, suggesting a potentially sensitive period in this age period. Together, these results support the importance of early and sustained obesity prevention strategies to reduce premature mortality.

## Contributors

HTL, MdS, JF and TS were involved in the conception and design of the study. CH, JW, KM, SS, PKEM, YTL, AC, SE, KI, SH, BIN, and LH contributed original cohort data. MdS harmonised the cohort data. JF and TS harmonised register data. HTL analysed data. HTL and TS drafted the manuscript. HTL, MdS, LB, AN, JF, and TS revised the manuscript. MdS, LB, AN, JF, and TS supervised the work. HTL, MdS, AN, JF, and TS had full access to all the data in the study and take responsibility for the integrity of the data and the accuracy of the analyses. All authors (HTL, MdS, LB, AE, CH, MS, IBM, JW, KM, SS, PKEM, YTL, AC, LL, SE, KI, SH, BIN, HK, LH, AN, JF, TS) made significant contributions to the manuscript, including reviewing and editing, and have read and approved the final version.

## Data sharing statement

All data are located on Statistics Sweden's Microdata Online Access (MONA) server and may only be accessed from countries in the European Union or the European Economic Area. Data access covered by the ethical approval will be considered in agreement with the principal investigator of ODDS, Tanja Stocks, and upon approval from register holders and steering committees of ODDS cohorts.

The analyses were conducted using Stata/MP version 18.0. The analytical code is not publicly available but can be shared with researchers upon reasonable request to the corresponding author.

## Declaration of interests

YTL reports receiving the Prince Daniel's Professorship in Cardiovascular Prevention (2024), funded through donations from the Swedish Heart-Lung Foundation, Werlabs, Region Stockholm, and Scania. YTL's department received payment for a lecture delivered to pharmacists. YTL received hourly compensation for expert work for the Dental and Pharmaceutical Benefits Agency and serves as an unpaid expert to the Public Health Agency of Sweden. YTL is an unpaid board member of the Swedish Association for the Study of Obesity (SFO) and is involved in distributing small research grants from a foundation supporting research on women's health. LB reports receiving a grant from the Swedish Foundation for Strategic Research. TS reports receiving grants from the Swedish Research Council, the Swedish Cancer Society, the Crafoord Foundation, and the Malmö General Hospital Cancer Foundation.
