# Supplementary material for: Weight trajectories and obesity onset between 17 and 60 years of age, and cause-specific mortality: the Obesity and Disease Development Sweden (ODDS) pooled cohort study
Source: eClinicalMedicine. 2026 Apr 10;94:103870. doi: 10.1016/j.eclinm.2026.103870 (PMC13133536; doi:10.1016/j.eclinm.2026.103870)
Supplement: Supplementary Material [file mmc1.docx]

**Supplementary Appendix**

**Weight trajectories and obesity onset between 17 and 60 years of age, and cause-specific mortality: the Obesity and Disease Development Sweden (ODDS) pooled cohort study**

Huyen T. Le, M.Sc.^1^, Marisa da Silva, Ph.D.^1,2^, Louise Bennet, M.D, Ph.D.^3^, Ahmed Elhakeem, Ph.D.^4,5^, Christel Häggström, Ph.D.^6^, Ming Sun, Ph.D.^1,7^, Innocent B. Mboya, Ph.D.^1,8,9^, Jens Wahlström, Ph.D.^10^, Karl Michaëlsson, M.D., Ph.D.^11^, Sven Sandin, Ph.D.^12,13^, Patrik K.E. Magnusson, Ph.D.^12^, Ylva Trolle Lagerros, Ph.D.^14^, Abbas Chabok, M.D, Ph.D.^15^, Lena Lönnberg, Ph.D.^16^, Sölve Elmståhl, Ph.D.^3^, Karolin Isaksson, Ph.D.^17,18^, Sara Hägg, Ph.D.^12^, Bright I. Nwaru, Ph.D.^19,20^, Hannu Kankaanranta, M.D., Ph.D.^19,21,22^, Linnea Hedman, Ph.D.^23^, Anton Nilsson, Ph.D.^1^, Josef Fritz, Ph.D.^1,24^, Tanja Stocks, Ph.D.^1^

^1^ Department of Translational Medicine, Lund University, Malmö, Sweden

^2^ School of Information Technology, Halmstad University, Halmstad, Sweden

^3^ Department of Clinical Sciences in Malmö, Lund University, Malmö, Sweden

^4^ Population Health Sciences, Bristol Medical School, University of Bristol, Bristol, UK

^5^ MRC Integrative Epidemiology Unit, University of Bristol, Bristol, UK

^6^ Northern Registry Centre, Department of Diagnostics and Intervention, Oncology, Umeå University, Umeå, Sweden

^7^ Department of Pharmacy, Xuanwu Hospital of Capital Medical University, Beijing, People's Republic of China.

^8^ Africa Academy for Public Health, Dar es Salaam, Tanzania

^9^ Department of Epidemiology and Biostatistics, School of Public Health, KCMC University, Moshi, Tanzania

^10^ Department of Epidemiology and Global Health, Umeå University, Umeå, Sweden

^11^ Medical Epidemiology, Department of Surgical Sciences, Uppsala University, Uppsala, Sweden

^12^ Department of Medical Epidemiology and Biostatistics, Karolinska Institutet, Stockholm, Sweden

^13^ Department of Psychiatry, Icahn School of Medicine at Mount Sinai, New York, USA

^14^ Department of Medicine, Huddinge, Karolinska Institutet, Stockholm, Sweden

^15^ Department of Clinical Sciences, Division of Surgery, Danderyd Hospital, Karolinska Institutet, Stockholm, Sweden

^16^ Centre for Clinical Research Västmanland, Uppsala university, Västerås, Sweden

^17^ Department of Clinical Sciences, Surgery, Lund University, Lund, Sweden

^18^ Department of Surgery, Skåne University Hospital, Kristianstad, Sweden

^19^ Krefting Research Centre, Department of Internal Medicine and Clinical Nutrition, Institute of Medicine, Sahlgrenska Academy, University of Gothenburg, Gothenburg, Sweden

^20^ Wallenberg Centre for Molecular and Translational Medicine, University of Gothenburg, Gothenburg, Sweden

^21^ Department of Respiratory Medicine, Seinäjoki Central Hospital, Wellbeing Services County of South Ostrobothnia, Seinäjoki, Finland

^22^ Faculty of Medicine and Health Technology, University of Tampere, Tampere, Finland

^23^ Department of Public Health and Clinical Medicine, The OLIN and Sunderby Research Unit, Umeå University, Umeå, Sweden

^24^ Institute of Clinical Epidemiology, Public Health, Health Economics, Medical Statistics and Informatics, Medical University of Innsbruck, Innsbruck, Austria

Corresponding author: Ms Huyen T. Le, Lund University, Department of Translational Medicine, Clinical Research Centre, Box 50332, 202 13, Malmö, Sweden. E-mail: [huyen.le@med.lu.se](mailto:huyen.le@med.lu.se)

CONTENTS

[SUPPLEMENTAL METHODS 5](#_Toc224125005)

[Obesity-related death causes 5](#_Toc224125006)

[Linear mixed-effects (LME) model 5](#_Toc224125007)

[Imputation of missing values 5](#_Toc224125008)

[Additional analyses 6](#_Toc224125009)

[SUPPLEMENTAL FIGURES 9](#_Toc224125010)

[Figure S1a. Hazard ratios (95% confidence intervals) of cause-specific mortality according to weight trajectories in quintiles in men. 9](#_Toc224125011)

[Figure S1b. Hazard ratios (95% confidence intervals) of cause-specific mortality according to weight trajectories in quintiles in women. 10](#_Toc224125012)

[Figure S2a. Hazard ratios (95% confidence intervals) of cause-specific mortality according to age of obesity onset in men 11](#_Toc224125013)

[Figure S2b. Hazard ratios (95% confidence interval) of cause-specific mortality according to age of obesity onset in women. 12](#_Toc224125014)

[Figure S3a. Hazard ratios (95% confidence intervals) of mortality from main causes according to weight change (kg/year) at ages 17–29, 30–44 and 45–60 years in men, allowing for non-linear effects. 13](#_Toc224125015)

[Figure S3b. Hazard ratios (95% confidence intervals) of mortality from main causes according to weight change (kg/year) at ages 17–29, 30–44 and 45–60 years in women, allowing for non-linear effects. 14](#_Toc224125016)

[Figure S4a. Hazard ratios (95% confidence intervals) of cause-specific mortality per 0.5 kg/year weight change at ages 17–29, 30–44 and 45–60 years in men. 15](#_Toc224125017)

[Figure S4b. Hazard ratios (95% confidence intervals) of cause-specific mortality per 0.5 kg/year weight change at ages 17–29, 30–44 and 45–60 years in women. 16](#_Toc224125018)

[Figure S5a. Hazard ratios (95% confidence intervals) of all-cause and cause-specific mortality per 0.5 kg/year weight change at ages 17–29, 30–44 and 45–60 years in men after excluding individuals with predicted weight loss in each age period. 17](#_Toc224125019)

[Figure S5b. Hazard ratios (95% confidence intervals) of all-cause and cause-specific mortality per 0.5 kg/year weight change at ages 17–29, 30–44 and 45–60 years in women after excluding individuals with predicted weight loss in each age period. 18](#_Toc224125020)

[Figure S6a. Hazard ratios (95% confidence intervals) of all-cause and cause-specific mortality per 1-SD weight change per year at ages 17–29, 30–44 and 45–60 years in men. 19](#_Toc224125021)

[Figure S6b. Hazard ratios (95% confidence intervals) of all-cause and cause-specific mortality per 1-SD weight change per year at ages 17–29, 30–44 and 45–60 years in women. 20](#_Toc224125022)

[SUPPLEMENTAL TABLES 21](#_Toc224125023)

[Table S1. Numbers and years of weight assessments by cohort, in men and women separately. 21](#_Toc224125024)

[Table S2. Definitions of causes of death according to International Classification of Diseases (ICD) codes and number of deaths. 22](#_Toc224125025)

[Table S3a. Characteristics of men according to weight trajectories and age of obesity onset 23](#_Toc224125026)

[Table S3b. Characteristics of women according to weight trajectories and age of obesity onset 24](#_Toc224125027)

[Table S4. Weight change in different age periods, and obesity onset, in the study population. 25](#_Toc224125028)

[Table S5. Hazard ratios (95% confidence intervals) of mortality from obesity-related cancers excluding sex-specific cancers per 0.5 kg/year weight change at ages 17–29, 30–44 and 45–60 years in men and women, respectively. 26](#_Toc224125029)

[Table S6. Hazard ratios (95% confidence intervals) of primary mortality outcomes in relation to weight trajectories at ages 17–60, age of obesity onset, and weight change in age periods in men and women separately with at least two weight assessments. 27](#_Toc224125030)

[Table S7. Hazard ratios (95% confidence intervals) of primary mortality outcomes in relation to weight trajectories at ages 17–60, age of obesity onset, and weight change in age periods in men and women with at least one weight assessment per age period 17–29, 30–44 and 45–60. 28](#_Toc224125031)

[Table S8. Hazard ratios (95% confidence intervals) of the five death causes with the strongest associations with smoking, in relation to weight trajectories at 17–60 years, age of obesity onset, and weight changes in age periods in men and women separately without adjusting for smoking information. 29](#_Toc224125032)

[Table S9. Hazard ratios (95% confidence intervals) of all-cause and cause-specific mortality in relation to per 5-unit BMI (kg/m^2^) at the last assessment, in men and women separately. 30](#_Toc224125033)

[Table S10. Hazard ratios (95% confidence intervals) of mortality from brain cancer in relation to weight trajectories at ages 17–60 years, age of obesity onset, and weight changes in age periods in men and women separately. 31](#_Toc224125034)

[Table S11. Hazard ratios (95% confidence intervals) and E-values for point estimates and lower confidence limits of hazard ratios of mortality associated with weight trajectory 5 vs weight trajectory 1. 32](#_Toc224125035)

[Table S12. Hazard ratios (95% confidence intervals) of primary mortality outcomes in relation to weight trajectories at ages 17–60, age of obesity onset, and weight change in age periods in men and women with five imputed data and complete data. 33](#_Toc224125036)

[References 34](#_Toc224125037)

SUPPLEMENTAL METHODS

# Obesity-related death causes

Cause-specific mortality was investigated only for death causes positively associated with obesity, defined as an increased risk for obesity (BMI >30 kg/m^2^) *vs.* normal weight (BMI 18.5–25 kg/m^2^) in a large prospective study by Bhaskaran *et al.^1^* This included mortality from: hypertension, ischemic heart disease, atrial fibrillation/flutter, heart failure, and aortic aneurysm; cancers of the oesophagus, stomach, colon, rectum, liver, pancreas, female breast, uterus, ovary, kidney, and bladder, and lymphoid neoplasms, multiple myeloma, and myeloid neoplasms; and from type 2 diabetes, digestive disease, and genitourinary disease. Additionally, mortality from prostate cancer and from cerebrovascular disease (including ischemic stroke and haemorrhagic stroke), were included based on findings for BMI and prostate cancer mortality in a large cohort study within the ODDS population,^2^ as well as in an umbrella review on adiposity and cardiovascular outcomes.^3^ Obesity-related cancer mortality, one of the main outcomes, comprised all obesity-related cancer deaths identified in Bhaskaran’s study, plus death from prostate cancer.

# Linear mixed-effects (LME) model

In the linear mixed-effects models, level 1 (within-subject) represents repeated weight measurements over age for each individual, while level 2 (between-subject) captures differences in weight trajectories between individuals.

We first inspected the weight trajectory over ages 17–60 years by visualising the observed population weight over ages. We graphically evaluated the fit of different linear mixed-effects (LME) models for weight using linear, quadratic and cubic splines of age, allowing random intercepts and slopes. The best fit was the LME model with a natural cubic spline of age (four knots) because it most closely matched the shape of the observed population trajectory. Additionally, the linearity constraints at the tails of the natural splines made the weight trajectories less erratic for the lowest and highest ages.^4^ In the selected model, fixed effects included a natural cubic spline of age (4 knots at the 5^th^, 35^th^, 65^th^, and 95^th^ percentiles), type of weight measurement (measured, current self-reported, or recalled self-reported), and pregnancy status at the time of measurement (for women). Random effects included an individual-specific random intercept and individual-specific random slope for age, capturing deviations from the population-average trajectory.

For weight changes in age periods, we used linear spline LME models with random intercept and slope to estimate weight change within each age period (17–29, 30–44, and 45–60 years). Linear splines were chosen due to the approximately linear shape of observed population weight within each of these three age periods and the simpler interpretability of linear spline slope coefficients.^4^ In the model, fixed effects included linear spline of age (2 knots at ages 30 and 45), type of weight measurement, and pregnancy status (for women). Random effects included an individual-specific random intercept and individual-specific random slopes for age within each period, allowing individual weight change to vary across age intervals. Individual-specific slopes for each age period were derived by combining the fixed and random slopes.

# Imputation of missing values

Among the co-variables, current smoking had the highest proportion of missingness: 1.8% in men and 14.5% in women. Other co-variables (height, education level, birth country, and marital status) had less than 1% missing data.

We assumed smoking data were missing at random (MAR), conditional on cohorts, because missing information primarily occurred in specific cohorts and during years when smoking information were not collected. The Medical Birth Register accounted for 90% of the missingness in women. In this cohort, the completeness was high for smoking status at the time of weight assessment, i.e., around gestational week 8-10 when the pregnancy was already known and likely had triggered a substantial proportion of smoking women to quit smoking. For this reason, we did not use this information but rather the much less complete information on smoking status 3 months before pregnancy. Though height, educational level, marital status and birth country could be sensitive to departure from MAR assumption, their small fraction of missingness might insubstantially affect methods of handling missing data.

We used multiple imputation by chained equations (MICE), with linear regression to impute continuous variables, logistic regression to impute binary variables, and multicategory logistic regression to impute multinominal variables. As recommended in the literature, the imputation model included all co-variables in the analysis model^5^ (some of them might also be able to predict the missing values), outcome information^6^ (event indicator of all-cause death, and the Nelson-Aalen estimator of the cumulative hazard function), cohort information (ability to predict smoking missingness)^5^, and death from respiratory disease or lung cancer (ability to predict missing smoking data)^5^.

We generated five imputed datasets, which has been deemed sufficient.^5^ The relatively low number of imputations was chosen due to the computational intensity associated with the large sample size, and due to the fairly low proportion of missing data. The imputation was done separately by sex, and results were pooled using Rubin’s rule.^5^ MICE imputation was performed using the *mi imputed chained* command in Stata.

# Additional analyses

**Analyses performed for weight changes in age periods**

For weight changes in age periods, we performed sensitivity analyses that (i) excluded sex-specific cancers from obesity-related cancer deaths, (ii) excluded individuals losing weight, and (iii) used age-specific weight change per standard deviation (SD)/year increase instead of per 0.5 kg/year as exposure.

(i) Sex-specific cancers, i.e. prostate cancer in men, and cancers of the breast, uterus and ovaries in women, were excluded in an additional analysis of obesity-related cancer deaths to investigate whether the differential findings for men and women from the age-period analyses of obesity-related cancer death were due to the inclusion of different cancers for men and women.

(ii) The analysis that excluded individuals losing weight in each age period was performed due to observed non-linear associations with mortality in older ages in our study, potentially influenced by unintentional weight loss following comorbidity. To enhance linearity and minimise the impact of J-shaped associations, we restricted the age-period analyses to individuals who did not lose weight within each age period. The estimated weight change for each individual in each age period (17–29, 30–44, and 45–60 years) were used to determine whether they lost weight during that period. In the Cox regression analysis for each age period, we excluded individuals losing weight only within that specific period, regardless of their weight changes in the other two periods.

(iii) We analysed mortality per 1-SD/year weight change instead of per 0.5 kg/year. This relative metric provides a different but relevant interpretation compared to the absolute metric of 0.5 kg/year, as it accounts for the larger weight gain in younger ages compared to older ages.

**Analyses performed for all exposures**

For all exposures, we performed sensitivity analyses that (i) included individuals with at least two weight assessments in addition to those with at least three weight assessments, ii) included only individuals with at least one weight assessment per age period, 17–29, 30–44 and 45–60 years, (iii) were unadjusted for smoking (for the outcomes most strongly associated with smoking), (iv) used BMI at the last assessment as exposure, and (v) used brain cancer mortality as a negative control outcome.

(i) We included individuals with at least two weight assessments to assess potential selection bias, as our main analysis was conducted on individuals with at least three weight assessments. Individuals with more weight assessments may, for example, be healthier and more engaged in participating in cohort examinations. Additionally, 60% of weight assessments in women were from the MBR; thus, our main study population is overrepresented by women with higher parity.

(ii) To assess potential misclassification bias, we conducted an analysis restricted to individuals with at least one weight assessment in each age period, 17–29, 30–44, and 45–60 years. This addressed our concern that, in the main analysis, weight assessments may not have been evenly distributed across the three age periods, potentially reducing the precision of individual-level weight change estimates in certain age periods. This restriction also resulted in a population with more repeated measurements per individual; the median number of weight assessments (interquartile range) was 5 (4 to 6) for both men and women.

(iii) We conducted an analysis unadjusted for smoking to compare the results with the smoking-adjusted results in our main analysis. This aimed to estimate the directional effect of smoking adjustment on the association between weight changes and mortality, which would indicate whether our findings may be underestimated or overestimated due to the lack of more detailed smoking information in the study. This analysis was performed for the five mortality outcomes with the strongest associations with smoking in our data. These were mortality from oesophageal cancer, liver cancer, ischemic heart disease, aortic aneurysm and digestive disease in men, and all-cause mortality, pancreas cancer, cardiovascular disease, ischemic heart disease and digestive disease mortality in women. These outcomes were all positively associated with smoking; we did not observe any cause of death negatively associated with smoking.

(iv) The analysis of BMI at the last assessment and mortality was conducted in response to the weak associations observed for *all* exposures (i.e. weight trajectories, age at obesity onset, and weight changes in age periods) with certain cancer-specific causes of death in our study. This result was unexpected, as our study focused on obesity-related mortality and all outcomes were therefore expected, a priori, to be associated with at least one exposure metric. Consequently, we explored the association between BMI and mortality in our population.

(v) To contrast the analysis of exclusively obesity-related death causes in this study, we employed a negative control outcome analysis, which could detect potential analytical errors and sources of bias. Brain cancer death was identified as the best negative control available in our data based on the following. First, we assumed that brain cancer death would not be associated with weight changes, based on the null association between BMI and brain cancer death in the study by Bhaskaran *et al.^1^*. Though this assumption was not entirely perfect because the findings were based on BMI rather than on weight changes, overall positive associations between the exposures of this study and brain cancer death would not be expected given a null association with BMI. Second, the confounders shared between the weight change-brain cancer mortality association were approximately similar to those in the weight change-mortality association under investigation, which is one of the conditions for a valid negative control outcome.^7^ Last, the number of brain cancer deaths in our population was sufficiently high (873 in men and 331 in women) to provide adequate statistical power.

**Post-hoc analyses**

We conducted post-hoc analyses, including (i) applying the E-value method to evaluate the robustness of observed associations with respect to unmeasured confounding, and (ii) performing complete-case analyses and comparing the results with those obtained using multiple imputation to evaluate the robustness of the imputation model.

(i) Because residual confounding due to unmeasured or imperfectly measured variables cannot be fully excluded in observational studies, we conducted an E-value analysis to assess the robustness of the observed associations to potential unmeasured confounding. The E-value quantifies the minimum strength of association that an unmeasured confounder would need to have with both the exposure and the outcome, beyond the measured covariates, to fully explain away the observed effect estimate.

(ii) To further evaluate the robustness of our handling of missing data, we performed complete-case analyses restricted to individuals with no missing information on any covariates included in the analysis model. We compared the effect estimates from the complete-case analyses with those obtained using multiple imputation to assess the consistency of the results across different approaches to handling missing data.

SUPPLEMENTAL FIGURES

# Figure S1a. Hazard ratios (95% confidence intervals) of cause-specific mortality according to weight trajectories in quintiles in men.

The number of men in trajectory Q1-Q4, was 51,654 each, and it was 51,653 in Q5. The weight trajectories were estimated using a linear mixed-effects model of weight with natural cubic splines of age (four knots). Other predictors in the linear mixed-effects model were the mode of weight measurement (measured, self-reported, recalled). Multivariable Cox regression with age as timescale was used to estimate the HRs and 95% CIs. The HRs were adjusted for predicted weight at age 17, height, highest attained education, birth country, marital status and current smoking at the last weight assessment, and stratified by birth decade. The HR per quintile was derived from a Cox model treating quintiles of weight trajectories as a continuous variable, adjusted for the same variables as above. HR: hazard ratio, CI: confidence interval, Q: quintile.

**Cardiovascular disease -specific**

**Cancer-specific**

**Other**


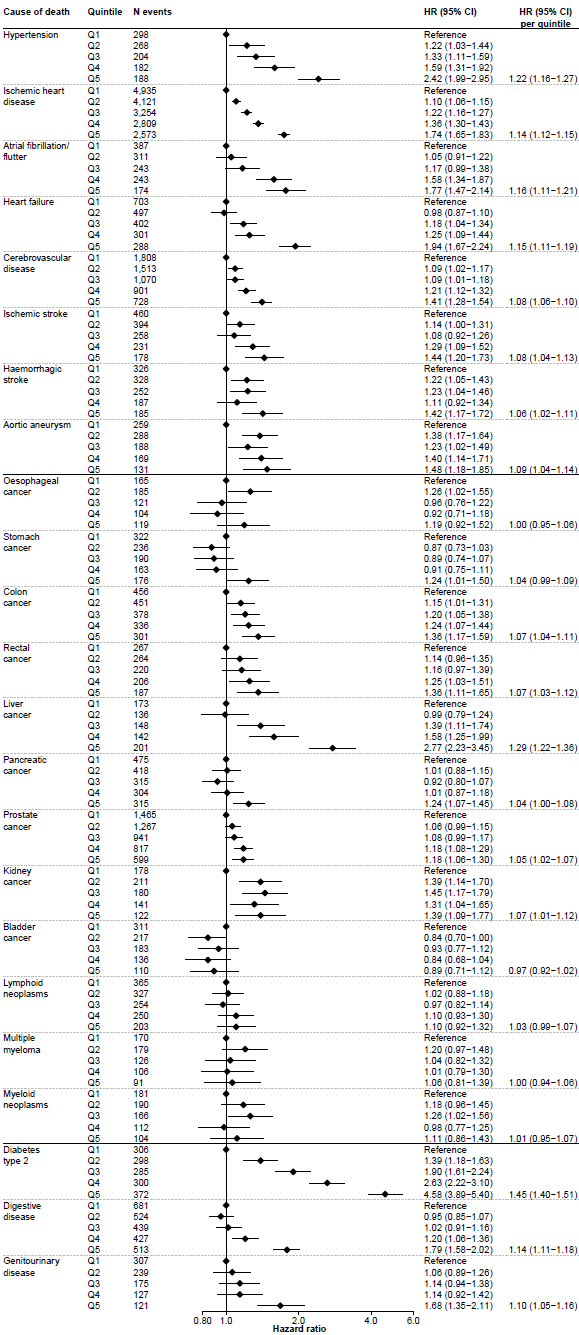


**Cardiovascular disease -specific**

**Cancer-specific**

**Others**


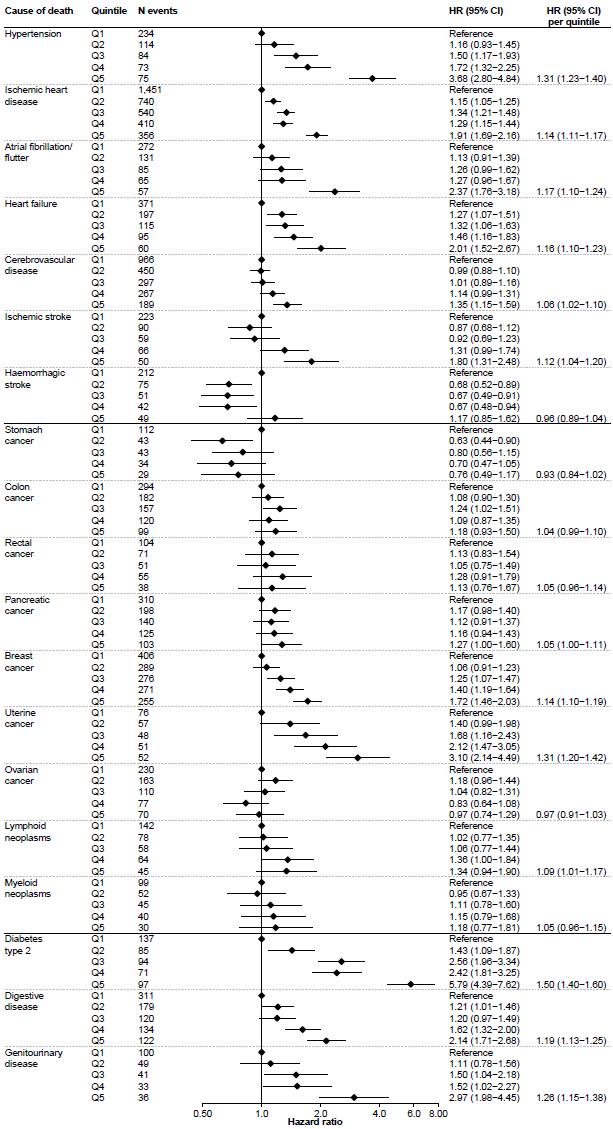


# Figure S1b. Hazard ratios (95% confidence intervals) of cause-specific mortality according to weight trajectories in quintiles in women.

The number of women in Q1-Q4 was 72,357 each, and it was 72,356 in Q5. The weight trajectories were estimated using a linear mixed-effects model of weight with natural cubic splines of age (four knots). Other predictors in the linear mixed-effects model were the mode of weight measurement (measured, self-reported, recalled) and pregnancy status at the time of weight assessment (yes, no). Multivariable Cox regression with age as timescale was used to estimate the HRs and 95% CIs. The HRs were adjusted for predicted weight at age 17, height, highest attained education, birth country, marital status andcurrent smoking at the last weight assessment, and stratified by birth decade. The HR per quintile was derived from a Cox model treating quintiles of weight trajectories as a continuous variable, adjusted for the same variables as above. HR: hazard ratio, CI: confidence interval, Q: quintile.

**Cardiovascular disease -specific**

**Cancer-specific**

**Other**


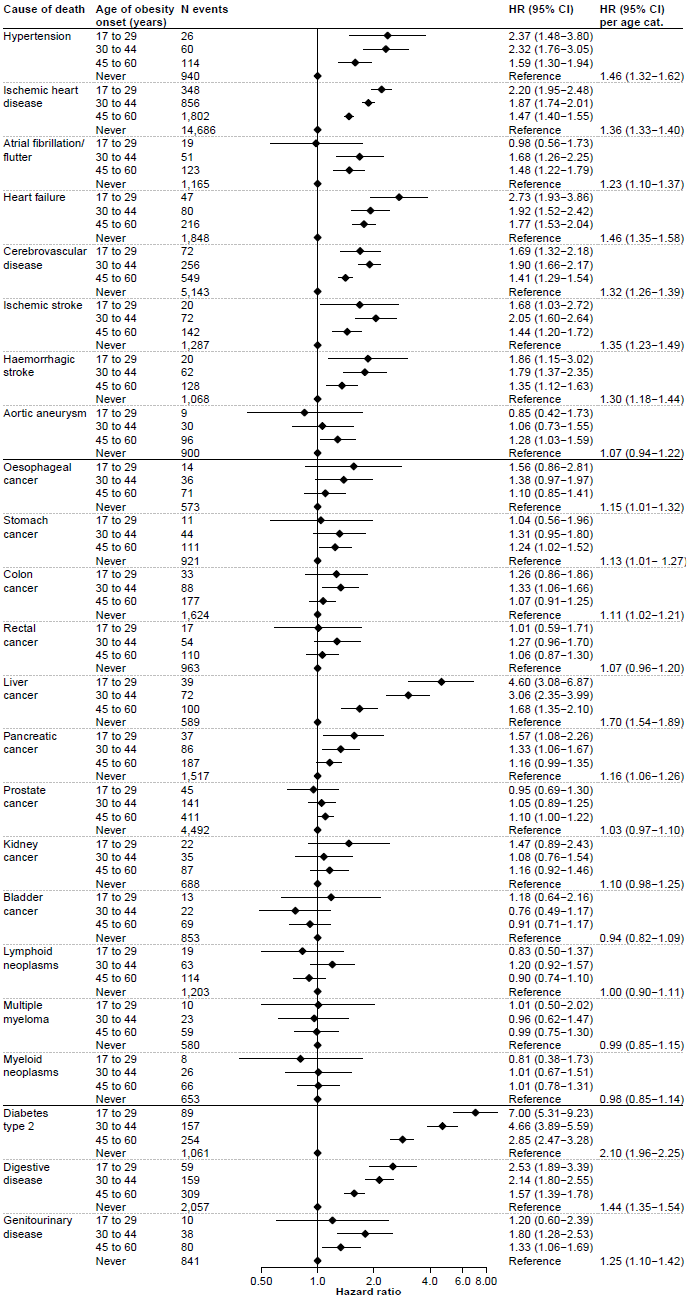


Figure S2a. Hazard ratios (95% confidence intervals) of cause-specific mortality according to age of obesity onset in men**.**

The number at risk for age of obesity onset at 17–29, 30–44, 45–60 years of age and never were 4,885, 13,570, 30,695 and 209,119*. Predicted individual weight within ages 17–60 years was derived from a linear mixed-effects model of weight using natural cubic splines of age (four knots). Other predictors in the linear mixed-effects model were the mode of weight measurement (measured, self-reported, recalled). Individual BMI at any age within 17–60 years was calculated as predicted individual weight at each age divided by measured height (kg/m^2^). Age at the first time with a predicted BMI ≥30 kg/m^2^ was treated as the age of obesity onset and used to classify into four groups, i.e. obesity onset at 17–29, 30–44, 45–60 and never developing obesity within ages 17–60 years (called never in the forest plot). Multivariable Cox regression with age as timescale was used to estimate the HRs and 95% CIs. The HRs were adjusted for predicted weight at age 17, highest attained education, birth country, marital status and current smoking at the last weight assessment, and stratified by birth decade. The HR per younger age category was derived from a Cox model treating group of obesity onset as a continuous variable, adjusted for the same variables as above.HR: hazard ratio, CI: confidence interval, excl.: excluding, cat.: category. *The number at risk and number of events were taken from imputed dataset 1 but varies slightly between imputed datasets.

**Cardiovascular disease -specific**

**Cancer-specific**

**Other**


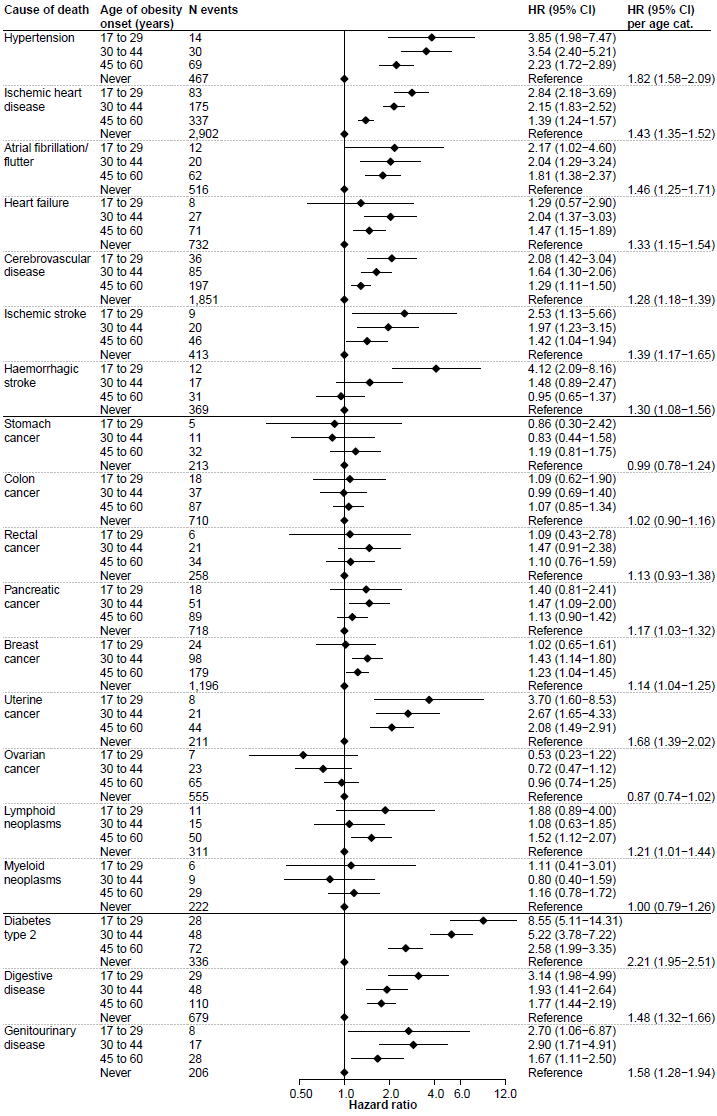


# Figure S2b. Hazard ratios (95% confidence interval) of cause-specific mortality according to age of obesity onset in women.

The number at risk for age of obesity onset at 17–29, 30–44, 45–60 years of age and never were 20,534, 34,103, 51,059 and 256,088*. Predicted individual weight within ages 17–60 years was derived from a linear mixed-effects model of weight using natural cubic splines of age (four knots). Other predictors in the linear mixed-effects model were the mode of weight measurement (measured, self-reported, recalled) and pregnancy status at the time of weight assessment (yes, no). Individual BMI at any age within 17–60 years was calculated as predicted individual weight at each age divided by measured height (kg/m^2^). Age at the first time with a predicted BMI ≥30 kg/m^2^ was treated as the age of obesity onset and used to classify into four groups, i.e. obesity onset at 17–29, 30–44, 45–60 and never developing obesity within ages 17–60 (called never in the forest plot). Multivariable Cox regression with age as timescale was used to estimate the HRs and 95% CIs. The HRs were adjusted for predicted weight at age 17, highest attained education, birth country, marital status and current smoking at the last weight assessment, and stratified by birth decade. The HR per younger age category was derived from a Cox model treating group of obesity onset as a continuous variable, adjusted for the same variables as above. HR: hazard ratio, CI: confidence interval, excl.: excluding, cat.: category. *The number at risk and number of events were taken from imputed dataset 1 but varies slightly between imputed datasets.

# Figure S3a. Hazard ratios (95% confidence intervals) of mortality from main causes according to weight change (kg/year) at ages 17–29, 30–44 and 45–60 years in men, allowing for non-linear effects.

The reference weight change (corresponding to a HR of 1.0) was 0 kg/year (i.e., stable weight). The individual weight change at ages 17–29, 30–44 and 45–60 years was the individual coefficient slope derived from a linear mixed-effects model of weight using linear splines of age at these periods. Other predictors in the linear mixed-effects model were the mode of weight measurement (measured, self-reported, recalled). Multivariable Cox regression with age as timescale and natural cubic spline of weight change (four knots) was used to estimate the non-linear association between weight change and HRs (95% CIs) of mortality. The HRs were adjusted for predicted weight at age 17, height, highest attained education, birth country, marital status and current smoking at the last weight assessment, and stratified by birth decade. The age periods 30–44 and 45–60 additionally included adjustment for weight change in the previous age periods. Solid lines are HR estimates, and shaded areas indicate 95% CI. p5 and p95 indicate percentile 5 and percentile 95 of the weight change in this age period. HR: hazard ratio, CI: confidence interval.


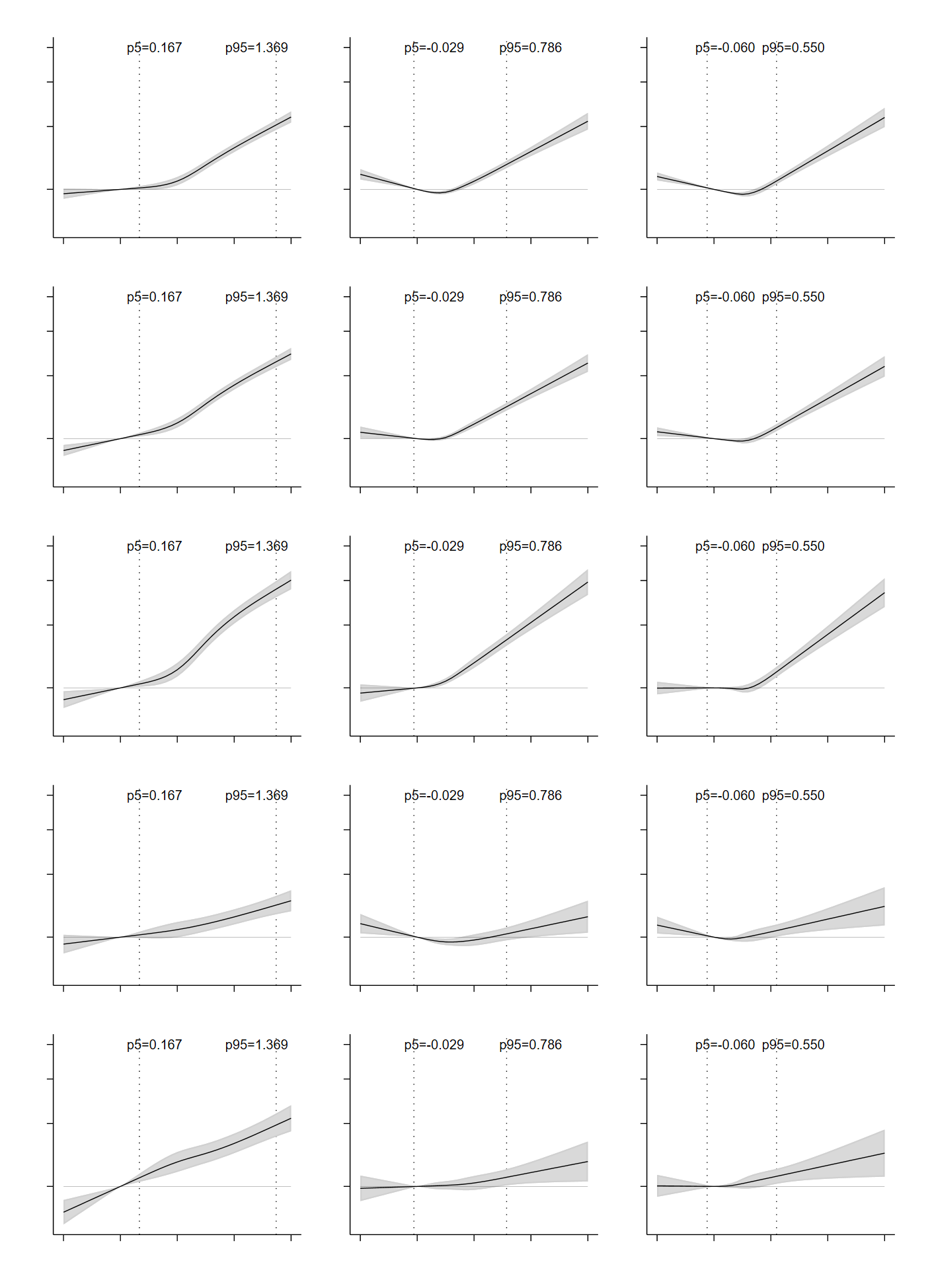


1.0

1.5

2.0

2.5

HR (95% CI)

1.0

1.5

2.0

2.5

HR (95% CI)

HR (95% CI)

1.0

1.5

2.0

2.5

HR (95% CI)

1.0

1.5

2.0

2.5

HR (95% CI)

All-cause mortality

All-cause mortality excl.
 respiratory disease & lung cancer

Cardiovascular disease mortality

All cancer mortality

Obesity-related cancer mortality

Age span 17─29 years

Age span 30─44 years

Age span 45─60 years

1.0

1.5

2.0

2.5

-0.5

0.0

0.5

1.0

1.5

Weight change (kg/year)

-0.5

0.0

0.5

1.0

1.5

Weight change (kg/year)

-0.5

0.0

0.5

1.0

1.5

Weight change (kg/year)

# Figure S3b. Hazard ratios (95% confidence intervals) of mortality from main causes according to weight change (kg/year) at ages 17–29, 30–44 and 45–60 years in women, allowing for non-linear effects.

The reference weight change (corresponding to a HR of 1.0) was 0 kg/year (i.e., stable weight). The individual weight change at ages 17–29, 30–44 and 45–60 years was the individual coefficient slope derived from a linear mixed-effects model of weight using linear splines of age at these periods. Other predictors in the linear mixed-effects model were the mode of weight measurement (measured, self-reported, recalled) and pregnancy status at the time of weight assessment (yes, no). Multivariable Cox regression with age as timescale and natural cubic spline of weight change (four knots) was used to estimate the non-linear association between weight change and HRs (95% CIs) of mortality. The HRs were adjusted for predicted weight at age 17, height, highest attained education, birth country, marital status and current smoking at the last weight assessment, and stratified by birth decade. The age periods 30–44 and 45–60 additionally included adjustment for weight change in the previous age periods. Solid lines are HR estimates, and shaded areas indicate 95% CI. p5 and p95 indicate percentile 5 and percentile 95 of the weight change in this age period. HR: hazard ratio, CI: confidence interval.


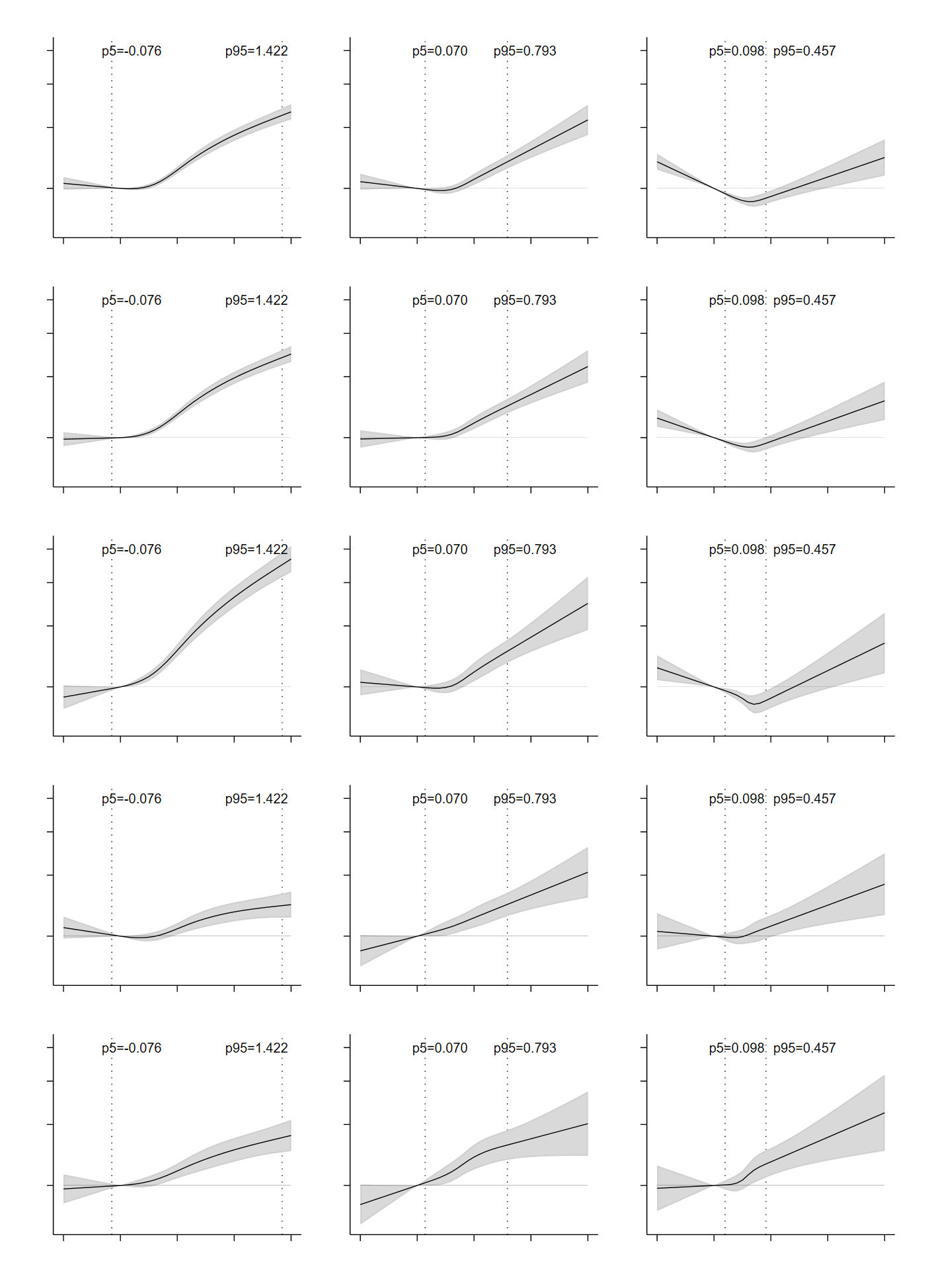


1.0

1.5

2.0

2.5

HR (95% CI)

1.0

1.5

2.0

2.5

HR (95% CI)

1.0

1.5

2.0

2.5

HR (95% CI)

1.0

1.5

2.0

2.5

HR (95% CI)

1.0

1.5

2.0

2.5

HR (95% CI)

All-cause mortality

All-cause mortality excl.
respiratory disease & lung cancer

Cardiovascular disease mortality

All cancer mortality

Obesity-related cancer mortality

Age span 17─29 years

Age span 30─44 years

Age span 45─60 years

-0.5

0.0

0.5

1.0

1.5

Weight change (kg/year)

-0.5

0.0

0.5

1.0

1.5

Weight change (kg/year)

-0.5

0.0

0.5

1.0

1.5

Weight change (kg/year)

**Cardiovascular disease -specific**

**Cancer-specific**

**Other**


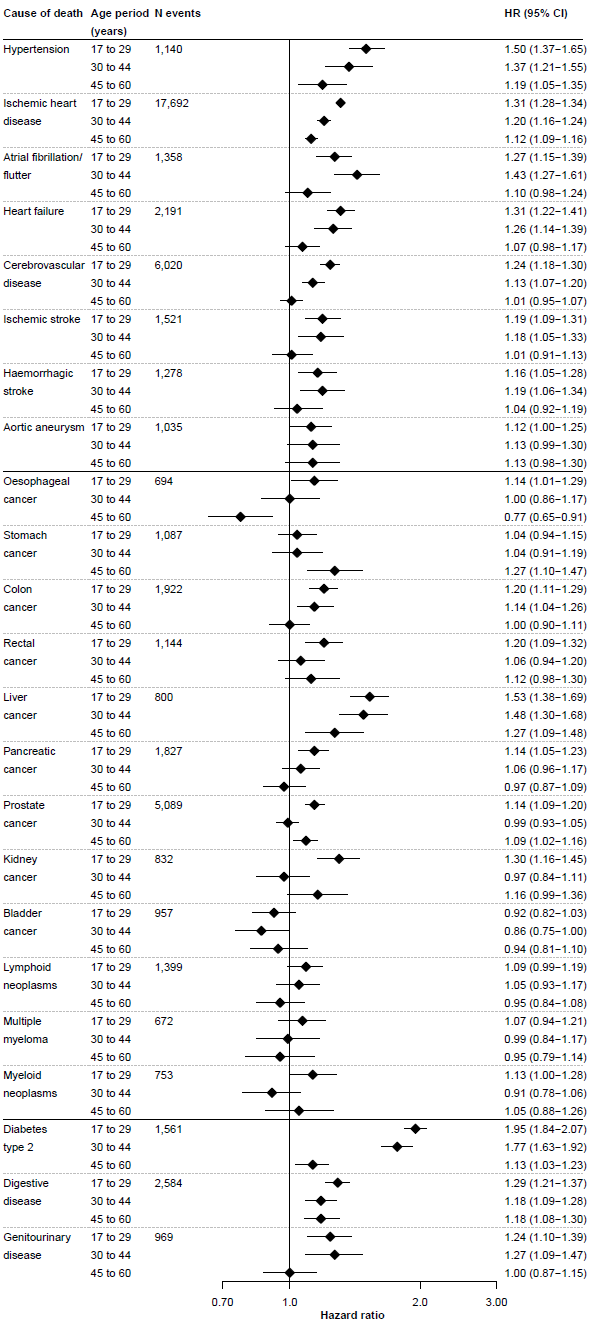


# Figure S4a. Hazard ratios (95% confidence intervals) of cause-specific mortality per 0.5 kg/year weight change at ages 17–29, 30–44 and 45–60 years in men.

The number at risk for each analysis was 258,269. The individual weight change at ages 17–29, 30–44 and 45–60 years was the individual coefficient slope derived from a linear mixed-effects model of weight using linear splines of age at these periods. Other predictors in the linear mixed-effects model were the mode of weight measurement (measured, self-reported, recalled). Multivariable Cox regression with age as timescale was used to estimate the HRs and 95% CIs. The HRs were adjusted for predicted weight at age 17, height, highest attained education, birth country, marital status and current smoking at the last weight assessment, and stratified by birth decade. The age periods 30–44 and 45–60 additionally included adjustment for weight change in the previous age periods. HR: hazard ratio, CI: confidence interval, excl.: excluding.

**Cardiovascular disease -specific**

**Cancer-specific**

**Other**


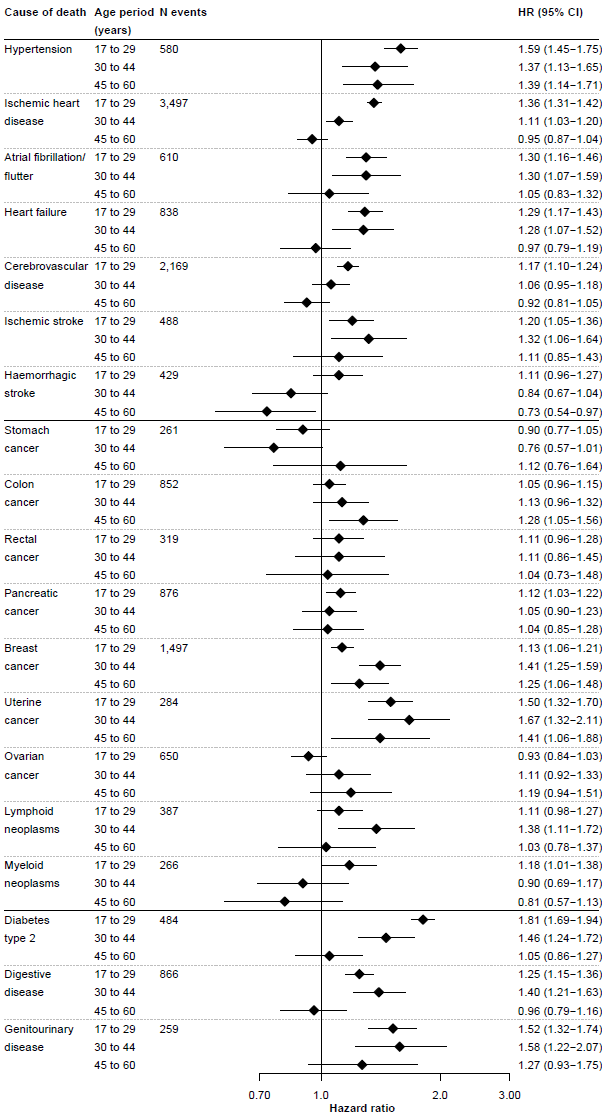


# Figure S4b. Hazard ratios (95% confidence intervals) of cause-specific mortality per 0.5 kg/year weight change at ages 17–29, 30–44 and 45–60 years in women.

The number at risk for each analysis was 361,784. The individual weight change at ages 17–29, 30–44 and 45–60 years was the individual coefficient slope derived from a linear mixed-effects model of weight using linear splines of age at these periods. Other predictors in the linear mixed-effects model were the mode of weight measurement (measured, self-reported, recalled) and pregnancy status at the time of weight assessment (yes, no). Multivariable Cox regression with age as timescale was used to estimate the HRs and 95% CIs. The HRs were adjusted for predicted weight at age 17, height, highest attained education, birth country, marital status and current smoking at the last weight assessment, and stratified by birth decade. The age periods 30–44 and 45–60 additionally included adjustment for weight change in the previous age periods. HR: hazard ratio, CI: confidence interval, excl.: excluding.

**Cardiovascular disease -specific**

**Cancer-specific**

**Other**

**Main**


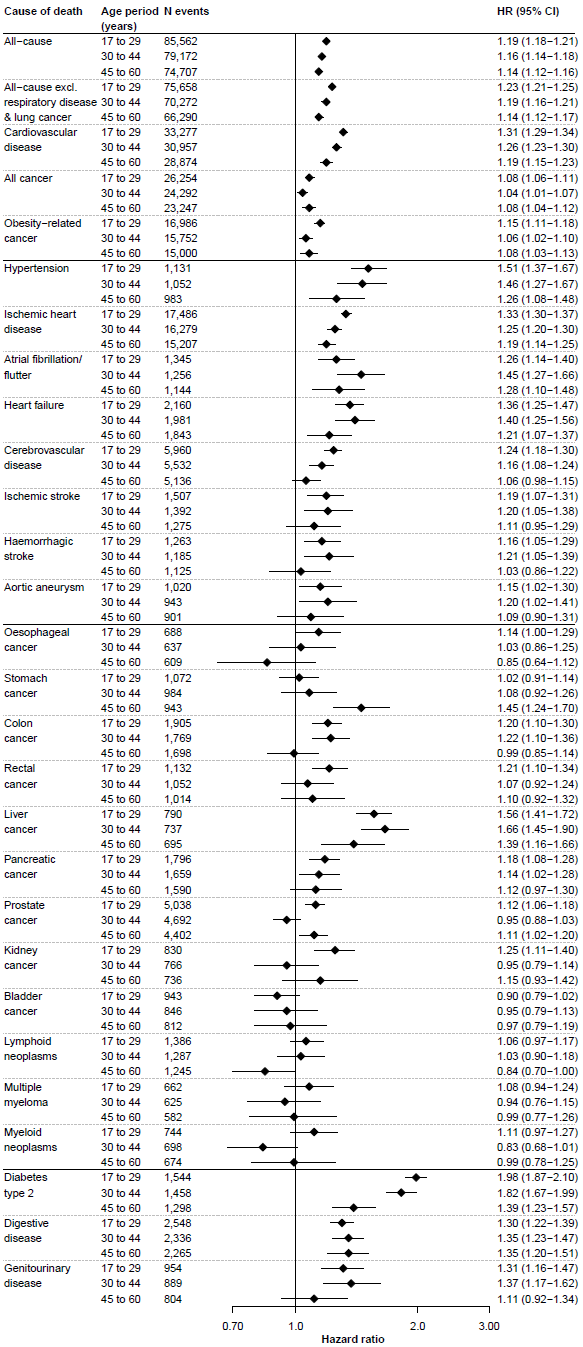


# Figure S5a. Hazard ratios (95% confidence intervals) of all-cause and cause-specific mortality per 0.5 kg/year weight change at ages 17–29, 30–44 and 45–60 years in men after excluding individuals with predicted weight loss in each age period.

The number at risk for analysis of ages 17─29, 30─44 and 45─60 years was 254,367, 241,641 and 238,604, respectively. The individual weight change at ages 17–29, 30–44 and 45–60 years was the individual coefficient slope derived from a linear mixed-effects model of weight using linear splines of age at these periods. Other predictors in the linear mixed-effects model were the mode of weight measurement (measured, self-reported, recalled). Multivariable Cox regression with age as timescale was used to estimate the HRs and 95% CIs. The HRs were adjusted for predicted weight at age 17 (continuous), height, highest attained education, birth country, marital status and current smoking at the last weight assessment, and stratified by birth decade. The age periods 30–44 and 45–60 additionally included adjustment for weight change in the previous age periods. HR: hazard ratio, CI: confidence interval, excl.: excluding.

**Cardiovascular disease -specific**

**Cancer-specific**

**Other**

**Main**


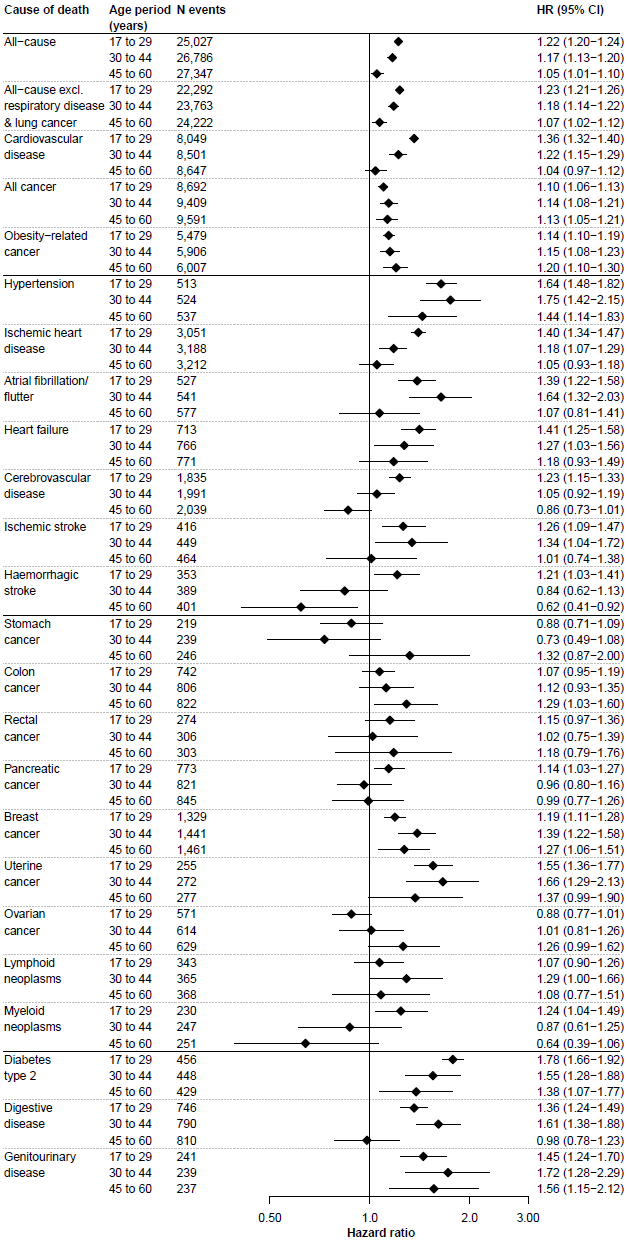


# Figure S5b. Hazard ratios (95% confidence intervals) of all-cause and cause-specific mortality per 0.5 kg/year weight change at ages 17–29, 30–44 and 45–60 years in women after excluding individuals with predicted weight loss in each age period.

The number at risk for analysis of ages 17─29, 30─44 and 45─60 years was 332,147, 350,934 and 355,410. The individual weight change at ages 17–29, 30–44 and 45–60 years was the individual coefficient slope derived from a linear mixed-effects model of weight using linear splines of age at these periods. Other predictors in the linear mixed-effects model were the mode of weight measurement (measured, self-reported, recalled) and pregnancy status at the time of weight assessment (yes, no). Multivariable Cox regression with age as timescale was used to estimate the HRs and 95% CIs. The HRs were adjusted for predicted weight at age 17, height, highest attained education, birth country, marital status and current smoking at the last weight assessment, and stratified by birth decade. The age periods 30–44 and 45–60 additionally included adjustment for weight change in the previous age periods. HR: hazard ratio, CI: confidence interval, excl.: excluding.

**Cardiovascular disease -specific**

**Cancer-specific**

**Other**

**Main**


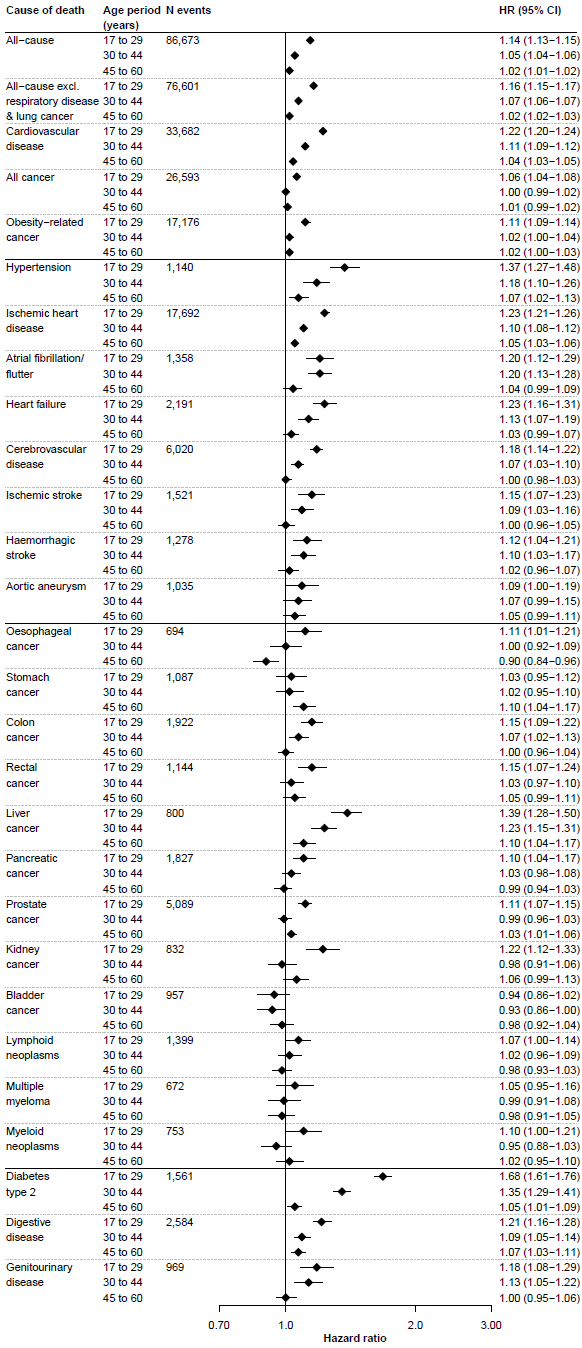


# Figure S6a. Hazard ratios (95% confidence intervals) of all-cause and cause-specific mortality per 1-SD weight change per year at ages 17–29, 30–44 and 45–60 years in men.

The number at risk for each analysis was 258,269. The 1-SD weight change at ages 17–29, 30–44, and 45–60 years was 0.387, 0.261, and 0.202 kg/year, respectively. The individual weight change at ages 17–29, 30–44 and 45–60 years was the individual coefficient slope derived from a linear mixed-effects model of weight using linear splines of age at these periods. Other predictors in the linear mixed-effects model were the mode of weight measurement (measured, self-reported, recalled). Multivariable Cox regression with age as timescale was used to estimate the HRs and 95% CIs. The HRs were adjusted for predicted weight at age 17, height, highest attained education, birth country, marital status and current smoking at the last weight assessment, and stratified by birth decade. The age periods 30–44 and 45–60 additionally included adjustment for weight change in the previous age periods. HR: hazard ratio, CI: confidence interval, excl.: excluding.

**Cardiovascular disease -specific**

**Cancer-specific**

**Other**

**Main**


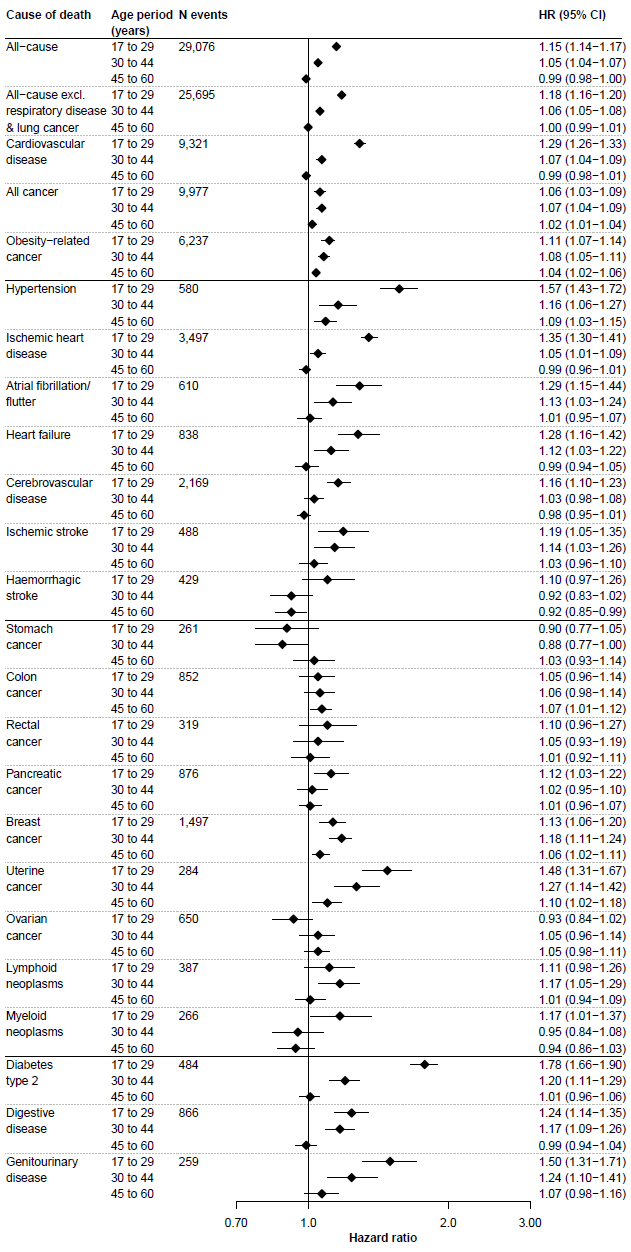


# Figure S6b. Hazard ratios (95% confidence intervals) of all-cause and cause-specific mortality per 1-SD weight change per year at ages 17–29, 30–44 and 45–60 years in women.

The number at risk for each analysis was 361,784. The 1-SD weight change at ages 17–29, 30–44, and 45–60 years was 0.483, 0.236, and 0.132 kg/year, respectively. The individual weight change at ages 17–29, 30–44 and 45–60 years was the individual coefficient slope derived from a linear mixed-effects model of weight using linear splines of age at these periods. Other predictors in the linear mixed-effects model were the mode of weight measurement (measured, self-reported, recalled) and pregnancy status at the time of weight assessment (yes, no). Multivariable Cox regression with age as timescale was used to estimate the HRs and 95% CIs. The HRs were adjusted for predicted weight at age 17, height, highest attained education, birth country, marital status and current smoking at the last weight assessment, and stratified by birth decade. The age periods 30–44 and 45–60 additionally included adjustment for weight change in the previous age periods. HR: hazard ratio, CI: confidence interval, excl.: excluding.

SUPPLEMENTAL TABLES

| Table S1. Numbers and years of weight assessments by cohort, in men and women separately. | | | | | | |
| --- | --- | --- | --- | --- | --- | --- |
| **Cohort** | **Men** | | **Women** | | |  |
|  | **N** | **Years**** | **N** | **Years**** |  |  |
| Construction Workers Cohort | 838,035 | 1971–1993 | 27,840 | 1971–1993 |  |  |
| National Medical Birth Register | - | - | 787,544 | 1982–2015 |  |  |
| SIMPLER** | 188,716 | 1938–2008 | 176,101 | 1934–2008 |  |  |
| Women's Lifestyle and Health** | - | - | 105,655 | 1960–2004 |  |  |
| Northern Sweden Health and Disease Study | 42,113 | 1985–2015 | 76,548 | 1985–2015 |  |  |
| Swedish Military Conscription Register | 91,557 | 1969–2014 | 4,552 | 1970–2014 |  |  |
| Swedish Twin Registry** | 30,441 | 1927–2013 | 40,137 | 1927–2013 |  |  |
| Swedish National March Cohort** | 17,001 | 1928–1998 | 34,053 | 1929–2006 |  |  |
| Malmö Cohorts*^,^ ** | 24,513 | 1943–2015 | 23,491 | 1943–2015 |  |  |
| Westmannia Cardiovascular Risk Factors Study | 6,604 | 1989–2000 | 7,776 | 1989–2000 |  |  |
| EpiHealth** | 5,717 | 1955–2015 | 8,625 | 1955–2015 |  |  |
| Melanoma in Southern Sweden** | - | - | 12,391 | 1944–2003 |  |  |
| LifeGene | 498 | 2009–2015 | 4,006 | 2009–2015 |  |  |
| West Sweden Asthma Study | 435 | 2008 | 2,437 | 2008 |  |  |
| Obstructive Lung Disease in Northern Sweden | 1,120 | 1986–2005 | 992 | 1986–2005 |  |  |
| **Total**** | **1,246,750** | **1927–2015** | **1,312,148** | **1927–2015** |  |  |
| *Includes the Malmö Preventive Project, the Malmö Diet and Cancer Study and the Malmö Offspring Study  SIMPLER: Swedish Infrastructure for Medical Population-based Life-course and Environmental Research.  **Also includes early years corresponding to ages for which weight was recalled. The earliest year of weight data collection was 1963 for both men and women. | | | | | | |

| Table S2. Definitions of causes of death according to International Classification of Diseases (ICD) codes and number of deaths. | | | | | | | | | | |
| --- | --- | --- | --- | --- | --- | --- | --- | --- | --- | --- |
| **Cause of death** | **ICD codes** | | | |  | | **N deaths** | | |  |
|  | **ICD-10** | **ICD-9** | **ICD-8** | |  | **Men** | | **Women** |  |  |
| All-cause | A00-Y89 | 001-E999 | 000-E999 | |  | 86,673 | | 29,076 |  |  |
| All-cause excluding respiratory disease & Lung/Trachea/Bronchus cancer | - | - | - | |  | 76,601 | | 25,695 |  |  |
| All cancer | C00-C97 | 140-208 | 140-209 | |  | 26,593 | | 9,977 |  |  |
| Obesity-related cancer* | - | - | - | |  | 17,176 | | 6,237 |  |  |
| Oesophagus | C15 | 150 | 150 | |  | 694 | | 96** |  |  |
| Lung/Trachea/Bronchus*** | C33-C34 | 162 | 162 | |  | 4,499 | | 1,514 |  |  |
| Stomach | C16 | 151 | 151 | |  | 1,087 | | 261 |  |  |
| Colon | C18 | 153 | 153 | |  | 1,922 | | 852 |  |  |
| Rectum | C19-20 | 154.0, 154.1 | 154.0, 154.1 | |  | 1,144 | | 319 |  |  |
| Liver | C22 | 155 | 155, 197.8 | |  | 800 | | 219* |  |  |
| Pancreas | C25 | 157 | 157 | |  | 1,827 | | 876 |  |  |
| Breast | C50 | 174-175 | 174 | |  | - | | 1,497 |  |  |
| Uterus | C54-C55 | 179, 182 | 182 | |  | - | | 284 |  |  |
| Ovary | C56 | 183.0 | 183.0 | |  | - | | 650 |  |  |
| Prostate | C61 | 185 | 185 | |  | 5,089 | | - |  |  |
| Kidney | C64 | 189.0 | 189.0 | |  | 832 | | 193** |  |  |
| Bladder | C67 | 188 | 188 | |  | 957 | | 142** |  |  |
| Lymphoid neoplasms | C81-85, C88, C91, C96 | 200-202, 204 | 200-202, 204 | |  | 1,399 | | 387 |  |  |
| Multiple myeloma | C90 | 203 | 203 | |  | 672 | | 195** |  |  |
| Myeloid neoplasms | C92-95, D46, D47.1 | 205-207, 209 | 205-207, 209 | |  | 753 | | 266 |  |  |
| Cardiovascular disease | I00-I99 | 390-459 | 390-444.1, 444.3-458, 782.4 |  | | 33,682 | | 9,321 |  |  |
| Hypertension | I10-15 | 401-405 | 400-404 |  | | 1,140 | | 580 |  |  |
| Ischemic heart disease | I20-25 | 410-414 | 410-414 |  | | 17,692 | | 3,497 |  |  |
| Atrial fibrillation/flutter | I48 | 427.3 | 427.4 |  | | 1,358 | | 610 |  |  |
| Heart failure | I50 | 428 | 427.0, 427.1 |  | | 2,191 | | 838 |  |  |
| Cerebrovascular disease | I60-I69 | 430-438 | 430-438 |  | | 6,020 | | 2,169 |  |  |
| - Ischaemic stroke | I63 | 434 | 433-434 |  | | 1,521 | | 488 |  |  |
| - Haemorrhagic stroke | I61 | 431 | 431 |  | | 1,278 | | 429 |  |  |
| Aortic aneurysm | I71.1-9 | 441.1-9 | 441.1-9 |  | | 1,035 | | 152** |  |  |
| Other |  |  |  |  | |  | |  |  |  |
| Diabetes type 2 | E11, E14 | 250 | 250 |  | | 1,561 | | 484 |  |  |
| Respiratory disease*** | J00-J99 | 460-519 | 460-519 |  | | 5,573 | | 1,867 |  |  |
| Digestive disease | K00-K92 | 520-579 | 520-577, 444.2 |  | | 2,584 | | 866 |  |  |
| Genitourinary disease | N00-N99 | 580-629 | 580-629, 792 |  | | 969 | | 259 |  |  |
| Negative control outcome |  |  |  |  | |  | |  |  |  |
| Brain cancer | C70-C72 | 191-192 | 191-192 |  | | 873 | | 331 |  |  |
| *Obesity-related cancer regards cancer deaths associated with BMI: the oesophagus, stomach, colon, rectum, liver, pancreas, kidney, bladder, prostate, uterus, ovary, female breast, lymphoid neoplasms, myeloid neoplasms, and multiple myeloma.  **Cause-specific mortality in men or women with less than 250 events was not analysed separately.  ***Not analysed as a separate outcome, but was excluded in the analysis of all-cause mortality excluding respiratory disease and Lung/Trachea/Bronchus Cancer, which was one of the main outcomes. | | | | | | | | | | |

| Table S3a. Characteristics of men according to weight trajectories and age of obesity onset. | | | | | | | | | | | |
| --- | --- | --- | --- | --- | --- | --- | --- | --- | --- | --- | --- |
| **Characteristic*** | **Weight trajectories in quintiles** | | | | |  | **Age of obesity onset**** | | | |  |
|  | **Quintile 1** | **Quintile 2** | **Quintile 3** | **Quintile 4** | **Quintile 5** |  | **Ages 17-29** | **Ages 30-44** | **Ages 45-60** | **Never by ages 60** | **Total** |
| N individuals | 51,654 | 51,654 | 51,654 | 51,654 | 51,653 |  | 4,884 | 13,570 | 30,696 | 209,119 | 258,269 |
| Age at last obs (yrs), median (IQR) | 54.4 (44.5–59.7) | 50.4 (39.3–58.6) | 45.8 (31.9–56.1) | 43.9 (30.8–54.2) | 44.4 (33.8–52.8) |  | 40.2 (29.4–50.6) | 41.4 (31.6–50.8) | 43.9 (32.4–53.2) | 49.8 (37.0–58.0) | 48.5 (35.7–57.3) |
| Calendar year of first obs, median (IQR) | 1972 (1962–1975) | 1973 (1971–1976) | 1974 (1972–1979) | 1974 (1972–1979) | 1974 (1972–1978) |  | 1975 (1972–1981) | 1975 (1972–1980) | 1974 (1972–1979) | 1973 (1971–1976) | 1973 (1971–1977) |
| Years from first to last obs, median (IQR) | 17.8 (11.4–34.0) | 15.1 (8.8–29.2) | 13.0 (7.2–20.9) | 13.7 (7.7–21.3) | 16.7 (11.0–29.1) |  | 14.0 (8.4–21.8) | 14.7 (9.1–22.0) | 15.0 (9.2–22.7) | 15.6 (9.0–30.0) | 15.5 (9.1–29.1) |
| Calendar year of birth, median (IQR) | 1939 (1927–1949) | 1943 (1931–1952) | 1947 (1937–1957) | 1949 (1940–1958) | 1951 (1943–1958) |  | 1953 (1943–1962) | 1952 (1943–1960) | 1950 (1941–1959) | 1945 (1933–1954) | 1946 (1935–1955) |
| Weight at last obs (kg), mean (SD) | 72.1 (8.7) | 76.5 (7.8) | 79.3 (8.3) | 83.1 (9.2) | 92.6 (12.3) |  | 106.7 (15.7) | 99.1 (11.3) | 90.9 (9.1) | 77.4 (8.7) | 80.7 (11.7) |
| Weight change, 17–60 yrs (kg/yr), median (IQR) | 0.16 (0.09–0.21) | 0.31 (0.28–0.34) | 0.42 (0.39–0.45) | 0.53 (0.50–0.57) | 0.73 (0.66–0.85) |  | 0.62 (0.36–0.90) | 0.76 (0.60–0.95) | 0.67 (0.56–0.80) | 0.38 (0.25–0.49) | 0.42 (0.28–0.57) |
| Height (cm), mean (SD) | 175.8 (6.6) | 176.9 (6.4) | 178.0 (6.1) | 178.8 (6.1) | 180.0 (6.4) |  | 177.4 (7.2) | 177.8 (6.6) | 178.2 (6.5) | 177.9 (6.4) | 177.9 (6.6) |
| BMI at last obs (kg/m^2^), mean (SD) | 23.3 (2.5) | 24.5 (2.3) | 25.0 (2.5) | 26.0 (2.7) | 28.6 (3.6) |  | 33.9 (4.3) | 31.3 (2.9) | 28.6 (2.3) | 24.5 (2.3) | 25.5 (3.6) |
| BMI at last obs in categories, n (%)** |  |  |  |  |  |  |  |  |  |  |  |
| Underweight | 666 (1.3) | 144 (0.3) | 168 (0.3) | 73 (0.1) | 4 (<0.1) |  | 1 (<0.1) | 0 (0) | 0 (0) | 1,054 (0.5) | 1055 (0.4) |
| Normal weight | 40,332 (78.1) | 31,100 (60.2) | 25,609 (49.6) | 18,807 (36.4) | 7,642 (14.8) |  | 43 (0.9) | 3 (<0.1) | 1,240 (4.1) | 122,204 (58.5) | 123,490 (47.8) |
| Overweight | 10,085 (19.5) | 19,685 (38.1) | 24,285 (47.0) | 28,745 (55.7) | 28,218 (54.6) |  | 523 (10.7) | 4,697 (34.6) | 21,034 (68.5) | 84,764 (40.5) | 111,018 (43.0) |
| Obesity | 571 (1.1) | 725 (1.4) | 1,592 (3.1) | 4,029(7.8) | 15,789 (30.6) |  | 4,317 (88.4) | 8,870 (65.4) | 8,422 (27.4) | 1,097 (0.5) | 22,706 (8.8) |
| Current smoker at last obs, n (%) |  |  |  |  |  |  |  |  |  |  |  |
| No | 33,532 (64.9) | 34,870 (67.5) | 35,521 (68.8) | 36,494 (70.7) | 37,280 (72.2) |  | 3,394 (69.5] | 9,651 (71.1) | 22,024 (71.7) | 142,628 (68.2) | 177,697 (68.8) |
| Yes | 18,122 (35.1) | 16,784 (32.5) | 16,133 (31.2) | 15,160 (29.3) | 14,373 (27.8) |  | 1,490 (30.5) | 3,919 (28.9) | 8,672 (28.3) | 66,491 (31.8) | 80,572 (31.2) |
| Highest achieved education, n (%) |  |  |  |  |  |  |  |  |  |  |  |
| Pre–upper secondary school <9 yrs | 19,681 (38.1) | 17,193 (33.3) | 13,669 (26.5) | 11,881 (23.0) | 10,059 (19.5) |  | 1,073 (22.0) | 2,805 (20.7) | 7,053 (23.0) | 61,552 (29.4) | 72,483 (28.1) |
| Pre–upper secondary school 9 yrs | 3,644 (7.0) | 3,9047 (7.5) | 4,483 (8.7) | 4,979 (9.6) | 5,805 (11.2) |  | 554 (11.3) | 1,556 (11.4) | 3,270 (10.6) | 17,435 (8.3) | 22,815 (8.8) |
| Upper secondary school <3 yrs | 15,128 (29.3) | 17,029 (33.0) | 19,520 (37.8) | 20,956 (40.6) | 22,148 (42.9) |  | 2,273 (46.5) | 6,186 (45.6) | 13,007 (42.4) | 73,315 (35.1) | 94,781 (36.7) |
| Upper secondary school 3 yrs | 6,601 (12.8) | 7,008 (13.6) | 7,185 (13.9) | 7,081 (13.7) | 6,841 (13.2) |  | 511 (10.5) | 1,492 (11.0) | 3,802 (12.4) | 28,911 (13.8) | 34,716 (13.4) |
| Post–upper secondary school ≥1 yrs | 6,600 (12.8) | 6,520 (12.6) | 6,797 (13.1) | 6,757 (13.1) | 6,800 (13.2) |  | 473 (9.7) | 1,531 (11.3) | 3,564 (11.6) | 27,906 (13.4) | 33,474 (13.0) |
| Birth country, n (%) |  |  |  |  |  |  |  |  |  |  |  |
| Born in SE and both parents born in SE | 47,542 (92.0) | 47,487 (91.9) | 47,072 (91.1) | 46,855 (90.7) | 46,824 (90.7) |  | 4,375 (89.6) | 12,125 (89.4) | 27,697 (90.2) | 191,583 (91.6) | 235,780 (91.3) |
| Born in SE and one/both parents born abroad | 1,375 (2.7) | 1,663 (3.2) | 2,333 (4.5) | 2,507 (4.9) | 2,649 (5.1) |  | 306 (6.3) | 794 (5.8) | 1,556 (5.1) | 7,872 (3.8) | 10,527 (4.1) |
| Born abroad | 2,737 (5.3) | 2,504 (4.9) | 2,249 (4.4) | 2,292 (4.4) | 2,180 (4.2) |  | 203 (4.1) | 651 (4.8) | 1,444 (4.7) | 9,664 (4.6) | 11,962 (4.6) |
| Marital status at last obs, n (%) |  |  |  |  |  |  |  |  |  |  |  |
| Unmarried | 9,336 (18.1) | 11,054 (21.4) | 15,606 (30.2) | 17,260 (33.4) | 16,283 (31.5) |  | 2,220 (45.4) | 4,991 (36.8) | 10,074 (32.8) | 52,254 (25.0) | 69,539 (26.9) |
| Married/registered partner | 36,431 (70.5) | 35,419 (68.6) | 31,408 (60.8) | 29,990 (58.1) | 30,370 (58.8) |  | 2,323 (47.6) | 7,451 (54.9) | 17,820 (58.1) | 136,025 (65.0) | 163,618 (63.4) |
| Divorced or widow/–er from spouse/partner | 5,887 (11.4) | 5,182 (10.0) | 4,640 (9.0) | 4,403 (8.5) | 5,000 (9.7) |  | 341 (7.0) | 1,128 (8.3) | 2,802 (9.1) | 20,841 (10.0) | 25,112 (9.7) |
| Abbreviations: obs: observation, yr: year; BMI: body mass index, IQR: interquartile range, SD: standard deviation, SE: Sweden.  *Characteristics after multiple imputation of missing values.  **The age of obesity onset was defined based on the predicted BMI (predicted weight from a linear mixed-effects model divided by measured height in kg/m²). As a result, there is a slight mismatch between the age-of-onset groups and the observed BMI categories at the last observation. | | | | | | | | | | | |

| Table S3b. Characteristics of women according to weight trajectories and age of obesity onset. | | | | | | | | | | | |
| --- | --- | --- | --- | --- | --- | --- | --- | --- | --- | --- | --- |
|  | **Weight trajectories in quintiles** | | | | |  | **Age of obesity onset**** | | | |  |
| **Characteristic*** | **Quintile 1** | **Quintile 2** | **Quintile 3** | **Quintile 4** | **Quintile 5** |  | **Ages 17–29** | **Ages 30-44** | **Ages 45–60** | **Never by ages 60** | **Total** |
| N individuals | 72,357 | 72,357 | 72,357 | 72,357 | 72,356 |  | 20,533 | 34,107 | 51,058 | 256,086 | 361,784 |
| Age at last obs (yrs), median (IQR) | 47.5 (36.6–57.5) | 37.4 (32.6–50.5) | 35.5 (31.3–45.1) | 35.1 (30.9–42.8) | 35.2 (31.2–40.8) |  | 32.5 (29.2–36.5) | 34.4 (30.5–39.7) | 35.4 (31.2–42.8) | 38.5 (33.0–52.1) | 36.9 (32.1–50.0) |
| Calendar year of first obs, median (IQR) | 1981 (1964–1995) | 1992 (1976–2000) | 1993 (1982–2001) | 1993 (1983–2001) | 1994 (1984–2001) |  | 1998 (1992–2004) | 1995 (1986–2002) | 1993 (1983–2001) | 1988 (1973–1999) | 1992 (1977–2000) |
| Years from first to last obs, median (IQR) | 19.9 (10.0–33.8) | 10.2 (6.5–24.0) | 9.0 (6.0–18.6) | 9.2 (6.0–17.2) | 10.5 (7.0–17.1) |  | 8.5 (5.9–12.4) | 9.7 (6.4–15.0) | 10.0 (6.5–17.9) | 11.6 (7.0–27.7) | 10.7 (6.7–23.3) |
| Calendar year of birth, median (IQR) | 1955 (1943–1968) | 1964 (1951–1974) | 1966 (1956–1975) | 1968(1958–1976) | 1969 (1960–1976) |  | 1974 (1968–1980) | 1971 (1962–1977) | 1968 (1958–1976) | 1962 (1949–1973) | 1965 (1953–1974) |
| Weight at last obs (kg), mean (SD) | 58.5 (7.4) | 62.1 (6.4) | 66.4 (6.3) | 72.6 (6.9) | 87.0 (12.9) |  | 97.2 (14.8) | 85.1 (9.8) | 76.5 (7.7) | 63.5 (7.6) | 69.3 (13.2) |
| Weight change, 17–60 yrs (kg/yr), median (IQR) | 0.17 (0.10–0.22) | 0.31 (0.28–0.34) | 0.42 (0.39–0.44) | 0.54 (0.50–0.58) | 0.78 (0.69–0.94) |  | 0.87 (0.67–1.10) | 0.76 (0.63–0.92) | 0.62 (0.53–0.72) | 0.34 (0.24–0.44) | 0.42 (0.28–0.58) |
| Height (cm), mean (SD) | 163.9 (5.9) | 165.2 (5.8) | 166.1 (6.0) | 166.7 (6.1) | 167.1 (6.2) |  | 165.0 (6.6) | 165.3 (6.3) | 165.7 (6.1) | 165.9 (6.1) | 165.8 (6.0) |
| BMI at last obs (kg/m^2^), mean (SD) | 21.8 (2.4) | 22.8 (2.4) | 24.1 (2.4) | 26.2 (2.7) | 31.2 (4.6) |  | 35.7 (4.7) | 31.1 (2.8) | 27.8 (2.0) | 23.1 (2.5) | 25.2 (4.8) |
| BMI at last obs in categories, n (%)** |  |  |  |  |  |  |  |  |  |  |  |
| Underweight | 4,234 (5.9) | 917 (1.3) | 129 (0.2) | 12 (<0.1) | 3 (<0.1) |  | 5 (<0.1) | 2 (<0.1) | 3 (<0.1) | 5,284 (2.1) | 5,294 (1.5) |
| Normal weight | 61,887 (85.5) | 59,878 (82.7) | 49,884 (68.9) | 26,026 (36.0) | 3,244 (4.5) |  | 184 (0.9) | 52 (0.2) | 2,136 (4.2) | 198,550 (77.5) | 200,922 (55.5) |
| Overweight | 5,712 (7.9) | 10,993 (15.2) | 20,810 (28.8) | 39,809 (55.0) | 29,251 (40.4) |  | 1,206 (5.9) | 12,763 (37.4) | 41,277 (80.8) | 51,329 (20.0) | 106,575 (29.5) |
| Obesity | 524 (0.7) | 569 (0.8) | 1,531 (2.1) | 6,510 (9.0) | 39,859 (55.1) |  | 19,138 (93.2) | 21,290 (62.4) | 7,642 (15.0) | 923 (0.4) | 48,993 (13.5) |
| Current smoker at last obs, n (%) |  |  |  |  |  |  |  |  |  |  |  |
| No | 56,651 (78.3) | 60,119 (83.1) | 60,368 (83.4) | 59,700 (82.5) | 58,586 (81.0) |  | 15,829 (77.1) | 27,503 (80.6) | 41,951 (82.2) | 210,141 (82.1) | 295,424 (81.7) |
| Yes | 15,706 (21.7) | 12,238 (16.9) | 11,989 (16.6) | 12,657 (17.5) | 13,770 (19.0) |  | 4,704 (22.9) | 6,604 (19.4) | 9,107 (17.8) | 45,945 (17.9) | 66,360 (18.3) |
| Highest achieved education, n (%) |  |  |  |  |  |  |  |  |  |  |  |
| Pre–upper secondary school <9 yrs | 8,597 (11.9) | 5,226 (7.2) | 4,321 (6.0) | 4,657 (6.4) | 4,816 (6.7) |  | 1,267 (6.2) | 2,464 (7.2) | 3,731 (7.3) | 20,155 (7.9) | 27,617 (7.6) |
| Pre-upper secondary school 9 yrs | 5,498 (7.6) | 4,909 (6.8) | 5,085 (7.0) | 5,757 (8.0) | 7,476 (10.3) |  | 2,444 (11.9) | 3,493 (10.2) | 4,559 (8.9) | 18,229 (7.1) | 28,725 (7.9) |
| Upper secondary school <3 yrs | 19,176 (26.5) | 17,258 (23.8) | 17,365 (24.0) | 18,573 (25.7) | 20,940 (28.9) |  | 5,558 (27.1) | 9,581 (28.1) | 13,996 (27.4) | 64,177 (25.0) | 93,312 (25.8) |
| Upper secondary school 3 yrs | 9,301 (12.8) | 11,490 (15.9) | 13,164 (18.2) | 14,172 (19.6) | 15,792 (21.8) |  | 5,755 (28.0) | 7,797 (22.9) | 10,255 (20.1) | 40,112 (15.7) | 63,919 (17.7) |
| Post-upper secondary school ≥1 yrs | 29,785 (41.2) | 33,474 (46.3) | 32,422 (44.8) | 29,198 (40.3) | 23,332 (32.3) |  | 5,509 (26.8) | 10,772 (31.6) | 18,517 (36.3) | 113,413 (44.3) | 148,211 (41.0) |
| Birth country, n (%) |  |  |  |  |  |  |  |  |  |  |  |
| Born in SE and both parents born in SE | 60,657 (83.8) | 58,412 (80.7) | 57,031 (78.8) | 54,609 (75.5) | 51,990 (71.8) |  | 14,318 (69.7) | 23,636 (69.3) | 37,122 (72.7) | 207,623 (81.1) | 282,699 (78.1) |
| Born in SE and one/both parents born abroad | 4,599 (6.4) | 5,700 (7.9) | 6,037 (8.4) | 6,323 (8.7) | 6,710 (9.3) |  | 2,241 (10.9) | 3,109 (9.1) | 4,423 (8.7) | 19,596 (7.6) | 29,369 (8.1) |
| Born abroad | 7,101 (9.8) | 8,245 (11.4) | 9,289 (12.8) | 11,425 (15.8) | 13,656 (18.9) |  | 3,974 (19.4) | 7,362 (21.6) | 9,513 (18.6) | 28,867 (11.3) | 49,716 (13.8) |
| Marital status at last obs, n (%) |  |  |  |  |  |  |  |  |  |  |  |
| Unmarried | 13,315 (18.4) | 15,870 (21.9) | 17,713 (24.5) | 17,995 (24.9) | 19,103 (26.4) |  | 6,332 (30.8) | 9,276 (27.2) | 12,830 (25.1) | 55,558 (21.7) | 83,996 (23.2) |
| Married/registered partner | 46,566 (64.4) | 47,684 (65.9) | 46,855 (64.7) | 46,421 (64.1) | 44,095 (60.9) |  | 11,873 (57.8) | 20,894 (61.3) | 32,071 (62.8) | 166,783 (65.1) | 231,621 (64.0) |
| Divorced or widow/-er from spouse/partner | 12,476 (17.2) | 8,803 (12.2) | 7,789 (10.8) | 7,941 (11.0) | 9,158 (12.7) |  | 2,328 (11.4) | 3,937 (11.5) | 6,157 (12.1) | 33,745 (13.2) | 46,167 (12.8) |
| Abbreviations: obs: observation, yr: year; BMI: body mass index, IQR: interquartile range, SD: standard deviation, SE: Sweden.  *Characteristics after multiple imputation of missing values.  **The age of obesity onset was defined based on the predicted BMI (predicted weight from a linear mixed-effects model divided by measured height in kg/m²). As a result, there is a slight mismatch between the age-of-onset groups and the observed BMI categories at the last observation. | | | | | | | | | | | |

# Table S4. Weight change in different age periods, and obesity onset, in the study population.

|  | **Men**  **(N=258,269)** | **Women**  **(N=361,784)** |
| --- | --- | --- |
| **Weight change (kg/year) in age periods,**  **median (IQR)** |  |  |
| Ages 17–29 years | 0.64 (0.44–0.89) | 0.44 (0.21–0.74) |
| Ages 30–44 years | 0.32 (0.19–0.44) | 0.40 (0.29–0.50) |
| Ages 45–60 years | 0.22 (0.15–0.28) | 0.29 (0.23–0.33) |
| **Age of obesity onset (years), n (%)** |  |  |
| Ages 17–29 years | 4,884 (1.9) | 20,533 (5.7) |
| Ages 30–44 years | 13,570 (5.2) | 34,107 (9.4) |
| Ages 45–60 years | 30,696 (11.9) | 51,058 (14.1) |
| Never developing obesity within ages 17–60 years | 209,119 (81.0) | 256,086 (70.8) |

Abbreviations: IQR: interquartile range

| Table S5. Hazard ratios (95% confidence intervals) of mortality from obesity-related cancers excluding sex-specific cancers per 0.5 kg/year weight change at ages 17–29, 30–44 and 45–60 years in men and women, respectively. | | | | | |
| --- | --- | --- | --- | --- | --- |
| **Age periods, years** | **Men (Total N=258,269)** | |  | **Women (Total N=361,784)** | |
|  | **N events** | **HR (95% CI)** |  | **N events** | **HR (95% CI)** |
| 17 to 29 | 12,087 | 1.15 (1.12–1.19) |  | 3,806 | 1.11 (1.07–1.16) |
| 30 to 44 |  | 1.06 (1.02–1.10) |  |  | 1.07 (0.99–1.15) |
| 45 to 60 |  | 1.03 (0.99–1.08) |  |  | 1.12 (1.02–1.24) |

The individual weight change at ages 17–29, 30–44 and 45–60 years was the individual coefficient slope derived from a linear mixed-effects model of weight using linear splines of age at these periods. Other predictors in the linear mixed-effects model were the mode of weight measurement and pregnancy status at the time of weight assessment (for women). Multivariable Cox regression with age as timescale was used to estimate the HRs and 95% CIs. The HRs were adjusted for predicted weight at age 17, height, highest attained education, birth country, marital status and current smoking at the last weight assessment, and stratified by birth decade. The age periods 30–44 and 45–60 additionally included adjustment for weight change in the previous age periods. HR: hazard ratio, CI: confidence interval. Sex-specific cancers included prostate cancer in men, and cancer of breast, uterus and ovary in women.

| Table S6. Hazard ratios (95% confidence intervals) of primary mortality outcomes in relation to weight trajectories at ages 17–60, age of obesity onset, and weight change in age periods in men and women separately with at least two weight assessments. | | | | | | | | | | | |
| --- | --- | --- | --- | --- | --- | --- | --- | --- | --- | --- | --- |
|  |  | **All causes** | | **All-cause excluding respiratory disease & lung cancer** | | **Cardiovascular disease** | | **All cancer** | | **Obesity-related cancer** | |
|  | **N at risk** | **N events** | **HR (95% CI)** | **N events** | **HR (95% CI)** | **N events** | **HR (95% CI)** | **N events** | **HR (95% CI)** | **N events** | **HR (95% CI)** |
| **MEN (Total N=425,608)** |  |  |  |  |  |  |  |  |  |  |  |
| Weight trajectories, ages 17–60 years | | | | | | | | | | | |
| Quintile 1 | 85,122 | 39,302 | 1.00 (Reference) | 33,779 | 1.00 (Reference) | 15,561 | 1.00 (Reference) | 11,290 | 1.00 (Reference) | 7,137 | 1.00 (Reference) |
| Quintile 2 | 85,122 | 29,746 | 1.03 (1.01–1.04) | 26,320 | 1.06 (1.05–1.08) | 11,719 | 1.10 (1.07–1.13) | 9,028 | 1.00 (0.98–1.03) | 5,896 | 1.07 (1.03–1.11) |
| Quintile 3 | 85,121 | 18,718 | 1.04 (1.02–1.06) | 16,760 | 1.09 (1.07–1.11) | 7,128 | 1.17 (1.14–1.21) | 5,700 | 0.98 (0.95–1.01) | 3,659 | 1.06 (1.02–1.10) |
| Quintile 4 | 85,122 | 19,143 | 1.13 (1.11–1.15) | 17,276 | 1.20 (1.17–1.22) | 7,325 | 1.31 (1.28–1.35) | 5,937 | 1.03 (1.00–1.07) | 3,836 | 1.13 (1.08–1.17) |
| Quintile 5 | 85,121 | 18,228 | 1.34 (1.31–1.36) | 16,611 | 1.43 (1.40–1.46) | 7,174 | 1.68 (1.63–1.73) | 5,546 | 1.12 (1.08–1.16) | 3,612 | 1.25 (1.20–1.30) |
| Per quintile | 425,608 | 125,137 | 1.06 (1.06–1.07) | 110,746 | 1.08 (1.08–1.09) | 48,907 | 1.12 (1.12–1.13) | 37,501 | 1.02 (1.01–1.03) | 24,140 | 1.05 (1.04–1.06) |
| Age of obesity onset, years* | | | | | | | | | | | |
| 17 to 29 | 9,960 | 2,181 | 1.69 (1.62–1.78) | 2,025 | 1.71 (1.62–1.79) | 930 | 2.05 (1.91–2.21) | 539 | 1.18 (1.07–1.29) | 376 | 1.27 (1.13–1.42) |
| 30 to 44 | 23,181 | 5,345 | 1.47 (1.42–1.51) | 4,969 | 1.52 (1.48–1.57) | 2,261 | 1.77 (1.69–1.85) | 1,437 | 1.14 (1.08–1.20) | 989 | 1.25 (1.17–1.34) |
| 45 to 60 | 49,750 | 11,609 | 1.26 (1.23–1.28) | 10,614 | 1.29 (1.27–1.32) | 4,835 | 1.47 (1.42–1.51) | 3,358 | 1.08 (1.04–1.12) | 2,199 | 1.12 (1.07–1.18) |
| Never developing obesity by age 60 | 342,717 | 106,002 | 1.00 (Reference) | 93,138 | 1.00 (Reference) | 40,881 | 1.00 (Reference) | 32,167 | 1.00 (Reference) | 20,576 | 1.00 (Reference) |
| Per younger age category | 425,608 | 125,137 | 1.21 (1.20–1.23) | 110,746 | 1.23 (1.22–1.24) | 48,907 | 1.33 (1.31–1.35) | 37,501 | 1.07 (1.04–1.09) | 24,140 | 1.11 (1.08–1.13) |
| Per 0.5 kg/year weight changes at ages, years** | | | | | | | | | | | |
| 17 to 29 | 425,608 | 125,137 | 1.18 (1.17–1.19) | 110,746 | 1.21 (1.20–1.22) | 48,907 | 1.29 (1.27–1.31) | 37,501 | 1.08 (1.06–1.10) | 24,140 | 1.15 (1.12–1.17) |
| 30 to 44 |  |  | 1.09 (1.07–1.10) |  | 1.13 (1.11–1.14) |  | 1.20 (1.18–1.23) |  | 1.00 (0.98–1.03) |  | 1.03 (1.00–1.07) |
| 45 to 60 |  |  | 1.03 (1.02–1.05) |  | 1.05 (1.04–1.07) |  | 1.09 (1.07–1.12) |  | 1.02 (0.99–1.05) |  | 1.05 (1.02–1.09) |
| **WOMEN (Total N=1,046,307)***** |  |  |  |  |  |  |  |  |  |  |  |
| Weight trajectories, ages 17–60 years | | | | | | | | | | | |
| Quintile 1 | 209,262 | 22,557 | 1.00 (Reference) | 19,564 | 1.00 (Reference) | 7,281 | 1.00 (Reference) | 7,243 | 1.00 (Reference) | 4,381 | 1.00 (Reference) |
| Quintile 2 | 209,261 | 8,854 | 1.06 (1.03–1.08) | 7,888 | 1.09 (1.06–1.11) | 2,373 | 1.08 (1.03–1.13) | 3,688 | 1.07 (1.03–1.12) | 2,365 | 1.15 (1.09–1.21) |
| Quintile 3 | 209,262 | 6,786 | 1.09 (1.06–1.12) | 6,105 | 1.13 (1.09–1.16) | 1,700 | 1.16 (1.10–1.22) | 2,973 | 1.08 (1.03–1.13) | 1,891 | 1.15 (1.08–1.21) |
| Quintile 4 | 209,261 | 6,542 | 1.15 (1.12–1.18) | 5,894 | 1.19 (1.16–1.23) | 1,615 | 1.26 (1.19–1.33) | 2,889 | 1.11 (1.06–1.16) | 1,850 | 1.20 (1.13–1.27) |
| Quintile 5 | 209,261 | 7,159 | 1.38 (1.34–1.42) | 6,443 | 1.43 (1.39–1.48) | 1,885 | 1.74 (1.65–1.83) | 2,990 | 1.20 (1.15–1.26) | 1,902 | 1.29 (1.22–1.37) |
| Per quintile | 1,046,307 | 51,898 | 1.07 (1.06–1.08) | 45,894 | 1.08 (1.07–1.09) | 14,854 | 1.12 (1.11–1.14) | 19,783 | 1.04 (1.03–1.05) | 12,389 | 1.06 (1.05–1.07) |
| Age of obesity onset, years**** |  |  |  |  |  |  |  |  |  |  |  |
| 17 to 29 | 62,262 | 1,400 | 1.74 (1.63–1.85) | 1,282 | 1.77 (1.65–1.89) | 358 | 2.47 (2.18–2.80) | 461 | 1.04 (0.93–1.16) | 301 | 1.10 (0.96–1.25) |
| 30 to 44 | 93,905 | 2,836 | 1.44 (1.38–1.50) | 2,572 | 1.48 (1.41–1.54) | 779 | 1.94 (1.79–2.09) | 1,118 | 1.10 (1.03–1.18) | 688 | 1.10 (1.01–1.19) |
| 45 to 60 | 139,375 | 5,172 | 1.23 (1.19–1.27) | 4,651 | 1.25 (1.21–1.29) | 1,437 | 1.42 (1.34–1.50) | 2,151 | 1.11 (1.06–1.17) | 1,399 | 1.16 (1.10–1.23) |
| Never developing obesity by age 60 | 748,253 | 42,199 | 1.00 (Reference) | 37,119 | 1.00 (Reference) | 12,188 | 1.00 (Reference) | 15,959 | 1.00 (Reference) | 9,936 | 1.00 (Reference) |
| Per younger age category | 1,043,795 | 51,607 | 1.21 (1.19–1.23) | 45,624 | 1.22 (1.20–1.24) | 14,762 | 1.38 (1.34–1.42) | 19,689 | 1.05 (1.02–1.08) | 12,324 | 1.06 (1.03–1.10) |
| The weight trajectories were estimated using a linear mixed-effects model of weight with natural cubic splines of age, and the individual weight change at ages 17–29, 30–44 and 45–60 years was the individual coefficient slope derived from a linear mixed-effects model of weight using linear splines of age at these periods. Other predictors in the linear mixed-effects model were the mode of weight measurement and pregnancy status at the time of weight assessment (for women). Individual BMI at any age within 17–60 years was calculated as predicted individual weight at each age (derived from linear mixed-effects model for weight trajectories) divided by measured height (kg/m^2^). Age at the first time with a predicted BMI ≥30 kg/m^2^ was treated as the age of obesity onset and used to classify into groups of obesity onset. Multivariable Cox regression with age as timescale was used to estimate the HRs and 95% CIs. The HRs were adjusted for predicted weight at age 17, height (except in the obesity onset analysis), highest attained education, birth country, marital status and current smoking at the last weight assessment, and stratified by birth decade. In the analysis for weight changes in age periods, the age periods 30–44 and 45–60 additionally included adjustment for weight change in the previous age periods. The HR per higher quintile/younger age category of obesity onset was derived from a Cox model treating quintiles of weight trajectories/ group of obesity onset as a continuous variable, adjusted for the same variables as above. *The number at risk and number of events for obesity onset group were taken from imputed dataset 1 but varies slightly between imputed datasets. **For women, the analysis of weight changes across age periods could not be conducted because the linear mixed-effects model used to estimate weight change for different age periods did not converge. We believe that this was due to the low number of repeated assessments per woman (median: 2, IQR: 2 to 3) and the unequal distribution of assessments across the three age periods (17–29, 30–44, and 45–60). This resulted in a significant number of individuals lacking assessments in one or more of the age periods. ***Since estimated weight change data for ages 17–29, 30–44, and 45–60—also used in the imputation model—were unavailable, we were unable to perform multiple imputation for women. Therefore, we assigned a missing category for co-variables in Cox regression with missing information. ****We assigned the 2,512 women with age of obesity onset missing a missing category in the Cox regression analysis for women. For the analysis of mortality for younger age category in women, we excluded individuals with missing data on obesity onset. HR: hazard ratio, CI: confidence interval. | | | | | | | | | | | |

| Table S7. Hazard ratios (95% confidence intervals) of primary mortality outcomes in relation to weight trajectories at ages 17–60, age of obesity onset, and weight change in age periods in men and women with at least one weight assessment per age period 17–29, 30–44 and 45–60. | | | | | | | | | | | |
| --- | --- | --- | --- | --- | --- | --- | --- | --- | --- | --- | --- |
|  |  | **All causes** | | **All-cause excluding respiratory disease & lung cancer** | | **Cardiovascular disease** | | **All cancer** | | **Obesity-related cancer** | |
|  | **N at risk** | **N events** | **HR (95% CI)** | **N events** | **HR (95% CI)** | **N events** | **HR (95% CI)** | **N events** | **HR (95% CI)** | **N events** | **HR (95% CI)** |
| **MEN (Total N=60,355)** |  |  |  |  |  |  |  |  |  |  |  |
| Weight trajectories, ages 17–60 years | | | | | | | | | | | |
| Quintile 1 | 12,071 | 6,210 | 1.00 (Reference) | 5,366 | 1.00 (Reference) | 2,495 | 1.00 (Reference) | 1,745 | 1.00 (Reference) | 1,135 | 1.00 (Reference) |
| Quintile 2 | 12,071 | 4,881 | 0.99 (0.95–1.03) | 4,300 | 1.01 (0.97–1.05) | 1,985 | 1.05 (0.99–1.12) | 1,386 | 0.93 (0.86–1.00) | 923 | 0.96 (0.88–1.05) |
| Quintile 3 | 12,071 | 3,973 | 1.04 (0.99–1.08) | 3,513 | 1.07 (1.02–1.11) | 1,585 | 1.14 (1.07–1.21) | 1,218 | 0.97 (0.90–1.05) | 809 | 1.02 (0.93–1.12) |
| Quintile 4 | 12,071 | 3,353 | 1.18 (1.13–1.23) | 3,033 | 1.24 (1.19–1.30) | 1,350 | 1.39 (1.30–1.49) | 1,045 | 1.04 (0.96–1.12) | 702 | 1.12 (1.01–1.23) |
| Quintile 5 | 12,071 | 3,031 | 1.45 (1.39–1.52) | 2,751 | 1.55 (1.47–1.62) | 1,256 | 1.88 (1.75–2.03) | 903 | 1.13 (1.04–1.24) | 587 | 1.20 (1.08–1.34) |
| Per quintile | 60,355 | 21,448 | 1.09 (1.07–1.10) | 18,963 | 1.10 (1.09–1.12) | 8,671 | 1.15 (1.14–1.17) | 6,297 | 1.03 (1.01–1.05) | 4,156 | 1.05 (1.02–1.07) |
| Age of obesity onset, years* | | | | | | | | | | | |
| 17 to 29 | 802 | 284 | 1.88 (1.65–2.14) | 255 | 1.88 (1.63–2.16) | 127 | 2.43 (1.99–2.97) | 69 | 1.13 (0.86–1.47) | 47 | 1.13 (0.82–1.57) |
| 30 to 44 | 2,692 | 765 | 1.71 (1.59–1.85) | 709 | 1.80 (1.67–1.95) | 345 | 2.26 (2.02–2.53) | 205 | 1.21 (1.05–1.40) | 138 | 1.28 (1.07–1.53) |
| 45 to 60 | 6,358 | 1,708 | 1.32 (1.25–1.39) | 1,569 | 1.38 (1.31–1.45) | 756 | 1.67 (1.54–1.80) | 474 | 1.03 (0.94–1.14) | 314 | 1.07 (0.95–1.20) |
| Never developing obesity by age 60 | 50,503 | 18,691 | 1.00 (Reference) | 16,430 | 1.00 (Reference) | 7,443 | 1.00 (Reference) | 5,549 | 1.00 (Reference) | 3,657 | 1.00 (Reference) |
| Per younger age category | 60,355 | 21,448 | 1.29 (1.25–1.32) | 18,963 | 1.31 (1.28–1.35) | 8,671 | 1.48 (1.42–1.54) | 6,297 | 1.06 (1.01–1.12) | 4,156 | 1.09 (1.02–1.16) |
| Per 0.5 kg/year weight changes at ages, years | | | | | | | | | | | |
| 17 to 29 | 60,355 | 21,448 | 1.21 (1.18–1.24) | 18,963 | 1.26 (1.23–1.29) | 8,671 | 1.35 (1.30–1.40) | 6,297 | 1.07 (1.03–1.12) | 4,156 | 1.17 (1.11–1.23) |
| 30 to 44 |  |  | 1.14 (1.10–1.18) |  | 1.18 (1.14–1.22) |  | 1.31 (1.25–1.38) |  | 1.01 (0.94–1.07) |  | 1.00 (0.93–1.08) |
| 45 to 60 |  |  | 1.08 (1.05–1.11) |  | 1.09 (1.06–1.13) |  | 1.14 (1.09–1.20) |  | 1.12 (1.05–1.19) |  | 1.11 (1.03–1.20) |
| **WOMEN (Total N=61,877)** |  |  |  |  |  |  |  |  |  |  |  |
| Weight trajectories, ages 17–60 years | | | | | | | | | | | |
| Quintile 1 | 12,376 | 4,030 | 1.00 (Reference) | 3,472 | 1.00 (Reference) | 1,494 | 1.00 (Reference) | 1,037 | 1.00 (Reference) | 619 | 1.00 (Reference) |
| Quintile 2 | 12,375 | 3,249 | 1.00 (0.96–1.05) | 2,894 | 1.04 (0.99–1.09) | 1,216 | 1.06 (0.98–1.15) | 901 | 0.99 (0.91–1.08) | 553 | 1.03 (0.91–1.15) |
| Quintile 3 | 12,376 | 2,725 | 1.10 (1.05–1.16) | 2,463 | 1.16 (1.10–1.23) | 990 | 1.21 (1.12–1.32) | 847 | 1.07 (0.98–1.17) | 524 | 1.13 (1.01–1.28) |
| Quintile 4 | 12,375 | 2,386 | 1.16 (1.10–1.22) | 2,134 | 1.22 (1.15–1.29) | 834 | 1.32 (1.21–1.43) | 807 | 1.11 (1.01–1.23) | 538 | 1.29 (1.14–1.45) |
| Quintile 5 | 12,375 | 2,098 | 1.37 (1.30–1.45) | 1,893 | 1.47 (1.39–1.56) | 735 | 1.79 (1.63–1.97) | 723 | 1.15 (1.04–1.27) | 481 | 1.33 (1.18–1.51) |
| Per quintile | 61,877 | 14,488 | 1.08 (1.06–1.09) | 12,856 | 1.09 (1.08–1.11) | 5,269 | 1.14 (1.12–1.16) | 4,315 | 1.04 (1.02–1.06) | 2,715 | 1.08 (1.05–1.11) |
| Age of obesity onset, years* |  |  |  |  |  |  |  |  |  |  |  |
| 17 to 29 | 766 | 184 | 1.79 (1.51–2.11) | 166 | 1.80 (1.51–2.15) | 75 | 2.14 (1.64–2.81) | 39 | 1.00 (0.70–1.41) | 29 | 1.16 (0.77–1.74) |
| 30 to 44 | 2,634 | 495 | 1.59 (1.45–1.75) | 455 | 1.67 (1.51–1.84) | 186 | 2.04 (1.75–2.37) | 156 | 1.23 (1.04–1.45) | 113 | 1.43 (1.18–1.75) |
| 45 to 60 | 6,678 | 1,322 | 1.28 (1.21–1.36) | 1,196 | 1.31 (1.23–1.39) | 498 | 1.50 (1.37–1.65) | 423 | 1.13 (1.02–1.25) | 282 | 1.20 (1.06–1.37) |
| Never developing obesity by age 60 | 51,799 | 12,487 | 1.00 (Reference) | 11,039 | 1.00 (Reference) | 4,510 | 1.00 (Reference) | 3,697 | 1.00 (Reference) | 2,291 | 1.00 (Reference) |
| Per younger age category | 61,877 | 14,488 | 1.25 (1.21–1.29) | 12,856 | 1.27 (1.23–1.32) | 5,269 | 1.40 (1.33–1.48) | 4,315 | 1.09 (1.02–1.16) | 2,715 | 1.16 (1.08–1.25) |
| Per 0.5 kg/year weight changes at ages, years | | | | | | | | | | | |
| 17 to 29 | 61,877 | 14,488 | 1.23 (1.19–1.27) | 12,856 | 1.27 (1.23–1.32) | 5,269 | 1.45 (1.37–1.53) | 4,315 | 1.08 (1.02–1.14) | 2,715 | 1.18 (1.10–1.27) |
| 30 to 44 |  |  | 1.15 (1.10–1.20) |  | 1.19 (1.13–1.24) |  | 1.24 (1.15–1.34) |  | 1.14 (1.05–1.23) |  | 1.18 (1.07–1.30) |
| 45 to 60 |  |  | 1.02 (0.97–1.07) |  | 1.04 (0.99–1.10) |  | 1.03 (0.96–1.12) |  | 1.15 (1.06–1.25) |  | 1.25 (1.13–1.39) |
| The weight trajectories were estimated using a linear mixed-effects model of weight with natural cubic splines of age, and the individual weight change at ages 17–29, 30–44 and 45–60 years was the individual coefficient slope derived from a linear mixed-effects model of weight using linear splines of age at these periods. Other predictors in the linear mixed-effects model were the mode of weight measurement and pregnancy status at the time of weight assessment (for women). Individual BMI at any age within 17–60 years was calculated as predicted individual weight at each age (derived from linear mixed-effects model for weight trajectories) divided by measured height (kg/m^2^). Age at the first time with a predicted BMI ≥30 kg/m^2^ was treated as the age of obesity onset and used to classify into groups of obesity onset. Multivariable Cox regression with age as timescale was used to estimate the HRs and 95% CIs. The HRs were adjusted for predicted weight at age 17, height (except in the obesity onset analysis), highest attained education, birth country, marital status and current smoking at the last weight assessment, and stratified by birth decade. In the analysis for weight changes in age periods, the age periods 30–44 and 45–60 additionally included adjustment for weight change in the previous age periods. The HR per higher quintile/younger age category of obesity onset was derived from a Cox model treating quintiles of weight trajectories/ group of obesity onset as a continuous variable, adjusted for the same variables as above. *The number at risk and number of events for obesity onset group were taken from imputed dataset 1 but varies slightly between imputed datasets. HR: hazard ratio, CI: confidence interval. | | | | | | | | | | | |

| Table S8. Hazard ratios (95% confidence intervals) of the five death causes with the strongest associations with smoking, in relation to weight trajectories at 17–60 years, age of obesity onset, and weight changes in age periods in men and women separately without adjusting for smoking information. | | | | | | | | | | | |
| --- | --- | --- | --- | --- | --- | --- | --- | --- | --- | --- | --- |
| **MEN** |  | **Oesophageal cancer** | | **Liver cancer** | | **Aortic aneurysm** | | **Ischemic heart disease** | | **Digestive disease** | |
|  | **N at risk** | **N events** | **HR (95% CI)** | **N events** | **HR (95% CI)** | **N events** | **HR (95% CI)** | **N events** | **HR (95% CI)** | **N events** | **HR (95% CI)** |
| Weight trajectories, ages 17–60 years | | | | | | | | | | | |
| Quintile 1 | 51,654 | 165 | 1.00 (Reference) | 173 | 1.00 (Reference) | 259 | 1.00 (Reference) | 4,935 | 1.00 (Reference) | 681 | 1.00 (Reference) |
| Quintile 2 | 51,654 | 185 | 1.23 (1.00-1.52) | 136 | 0.98 (0.78–1.22) | 288 | 1.34 (1.13–1.59) | 4,121 | 1.09 (1.04–1.13) | 524 | 0.94 (0.84–1.05) |
| Quintile 3 | 51,654 | 121 | 0.94 (0.74–1.19) | 148 | 1.36 (1.09–1.70) | 188 | 1.17 (0.97–1.42) | 3,254 | 1.19 (1.13–1.24) | 439 | 1.00 (0.88–1.13) |
| Quintile 4 | 51,654 | 104 | 0.88 (0.69–1.14) | 142 | 1.54 (1.22–1.94) | 169 | 1.31 (1.07–1.60) | 2,809 | 1.32 (1.26–1.38) | 427 | 1.16 (1.02–1.31) |
| Quintile 5 | 51,653 | 119 | 1.12 (0.87–1.43) | 201 | 2.67 (2.15–3.32) | 131 | 1.35 (1.08–1.69) | 2,573 | 1.67 (1.58–1.75) | 513 | 1.70 (1.50–1.93) |
| Per quintile | 258,269 | 694 | 0.99 (0.93–1.04) | 800 | 1.28 (1.21–1.35) | 1,035 | 1.06 (1.01–1.11) | 17,692 | 1.12 (1.11–1.14) | 2,584 | 1.13 (1.10–1.16) |
| Age of obesity onset, years* | | | | | | | | | | | |
| 17 to 29 | 4,885 | 14 | 1.55 (0.86–2.80) | 39 | 4.56 (3.05–6.81) | 9 | 0.86 (0.43–1.74) | 348 | 2.19 (1.94–2.47) | 59 | 2.52 (1.88–3.38) |
| 30 to 44 | 13,570 | 36 | 1.36 (0.96–1.94) | 72 | 3.03 (2.32–3.94) | 30 | 1.05 (0.72–1.53) | 856 | 1.86 (1.73–2.00) | 159 | 2.12 (1.78–2.52) |
| 45 to 60 | 30,695 | 71 | 1.07 (0.83–1.37) | 100 | 1.65 (1.33–2.05) | 96 | 1.23 (0.99–1.53) | 1,802 | 1.44 (1.37–1.52) | 309 | 1.53 (1.36–1.74) |
| Never developing obesity by age 60 | 209,119 | 573 | 1.00 (Reference) | 589 | 1.00 (Reference) | 900 | 1.00 (Reference) | 14,686 | 1.00 (Reference) | 2,057 | 1.00 (Reference) |
| Per younger age category | 258,269 | 694 | 1.14 (1.00–1.31) | 800 | 1.69 (1.53–1.88) | 1,035 | 1.06 (0.93–1.20) | 17,692 | 1.35 (1.32–1.39) | 2,584 | 1.43 (1.34–1.53) |
| Per 0.5 kg/year weight changes at ages, years | | | | | | | | | | | |
| 17 to 29 | 258,269 | 694 | 1.13 (1.00–1.27) | 800 | 1.52 (1.38–1.68) | 1,035 | 1.11 (0.99–1.24) | 17,692 | 1.30 (1.27–1.33) | 2,584 | 1.28 (1.20–1.36) |
| 30 to 44 |  |  | 0.96 (0.82–1.12) |  | 1.44 (1.27–1.64) |  | 1.07 (0.93–1.23) |  | 1.17 (1.13–1.21) |  | 1.15 (1.06–1.24) |
| 45 to 60 |  |  | 0.74 (0.62–0.88) |  | 1.25 (1.07–1.45) |  | 1.07 (0.93–1.24) |  | 1.10 (1.06–1.13) |  | 1.15 (1.05–1.26) |
| **WOMEN** |  | **All-cause** | | **Pancreatic cancer** | | **Cardiovascular disease** | | **Ischemic heart disease** | | **Digestive disease** | |
|  | **N at risk** | **N events** | **HR (95% CI)** | **N events** | **HR (95% CI)** | **N events** | **HR (95% CI)** | **N events** | **HR (95% CI)** | **N events** | **HR (95% CI)** |
| Weight trajectories, ages 17–60 years | | | | | | | | | | | |
| Quintile 1 | 72,357 | 11,618 | 1.00 (Reference) | 310 | 1.00 (Reference) | 3,956 | 1.00 (Reference) | 1,451 | 1.00 (Reference) | 311 | 1.00 (Reference) |
| Quintile 2 | 72,357 | 6,030 | 1.01 (0.98–1.05) | 198 | 1.13 (0.94–1.35) | 1,981 | 1.09 (1.03–1.15) | 740 | 1.11 (1.01–1.21) | 179 | 1.15 (0.96–1.39) |
| Quintile 3 | 72,357 | 4,464 | 1.09 (1.06–1.13) | 140 | 1.07 (0.88–1.32) | 1,343 | 1.18 (1.11–1.25) | 540 | 1.28 (1.16–1.42) | 120 | 1.13 (0.91–1.40) |
| Quintile 4 | 72,357 | 3,807 | 1.11 (1.07–1.16) | 125 | 1.10 (0.89–1.37) | 1,132 | 1.25 (1.17–1.34) | 410 | 1.22 (1.09–1.36) | 134 | 1.50 (1.22–1.85) |
| Quintile 5 | 72,356 | 3,157 | 1.37 (1.31–1.42) | 103 | 1.21 (0.95–1.52) | 909 | 1.75 (1.62–1.89) | 356 | 1.81 (1.60–2.04) | 122 | 1.98 (1.58–2.48) |
| Per quintile | 361,784 | 29,076 | 1.06 (1.05–1.07) | 876 | 1.04 (0.99–1.09) | 9,321 | 1.12 (1.10–1.13) | 3,497 | 1.12 (1.10–1.15) | 866 | 1.16 (1.11–1.22) |
| Age of obesity onset, years* | | | | | | | | | | | |
| 17 to 29 | 20,534 | 596 | 1.69 (1.53–1.86) | 18 | 1.37 (0.79–2.37) | 178 | 2.21 (1.85–2.63) | 83 | 2.73 (2.10–3.56) | 29 | 3.03 (1.91–4.81) |
| 30 to 44 | 34,103 | 1,343 | 1.45 (1.37–1.54) | 51 | 1.44 (1.06–1.95) | 419 | 1.95 (1.76–2.17) | 175 | 2.08 (1.77–2.44) | 48 | 1.85 (1.36–2.53) |
| 45 to 60 | 51,059 | 2,819 | 1.20 (1.16–1.25) | 89 | 1.09 (0.87–1.37) | 889 | 1.37 (1.28–1.47) | 337 | 1.33 (1.19–1.49) | 110 | 1.67 (1.36–2.05) |
| Never developing obesity by age 60 | 256,088 | 24,318 | 1.00 (Reference) | 718 | 1.00 (Reference) | 7,835 | 1.00 (Reference) | 2,902 | 1.00 (Reference) | 679 | 1.00 (Reference) |
| Per younger age category | 361,784 | 29,076 | 1.20 (1.17–1.23) | 876 | 1.15 (1.02–1.30) | 9,321 | 1.36 (1.31–1.41) | 3,497 | 1.40 (1.32–1.49) | 866 | 1.45 (1.29–1.62) |
| Per 0.5 kg/year weight changes at ages, years | | | | | | | | | | | |
| 17 to 29 | 361,784 | 29,076 | 1.14 (1.12–1.16) | 876 | 1.10 (1.01–1.20) | 9,321 | 1.28 (1.24–1.31) | 3,497 | 1.33 (1.27–1.39) | 866 | 1.21 (1.11–1.32) |
| 30 to 44 |  |  | 1.08 (1.05–1.12) |  | 1.02 (0.87–1.19) |  | 1.12 (1.06–1.17) |  | 1.08 (1.00–1.16) |  | 1.35 (1.16–1.57) |
| 45 to 60 |  |  | 0.96 (0.93–0.99) |  | 1.02 (0.83–1.25) |  | 0.96 (0.91–1.02) |  | 0.94 (0.86–1.02) |  | 0.93 (0.77–1.13) |

The weight trajectories were estimated using a linear mixed-effects model of weight with natural cubic splines of age, and the individual weight change at ages 17–29, 30–44 and 45–60 years was the individual coefficient slope derived from a linear mixed-effects model of weight using linear splines of age at these periods. Other predictors in the linear mixed-effects model were the mode of weight measurement and pregnancy status at the time of weight assessment (for women). Individual BMI at any age within 17–60 years was calculated as predicted individual weight at each age (derived from linear mixed-effects model for weight trajectories) divided by measured height (kg/m^2^). Age at the first time with a predicted BMI ≥30 kg/m^2^ was treated as the age of obesity onset and used to classify into groups of obesity onset. Multivariable Cox regression with age as timescale was used to estimate the HRs and 95% CIs. The HRs were adjusted for predicted weight at age 17, height (except in the obesity onset analysis), highest attained education, birth country, marital status and current smoking at the last weight assessment, and stratified by birth decade. In the analysis for weight changes in age periods, the age periods 30–44 and 45–60 additionally included adjustment for weight change in the previous age periods. The HR per higher quintile/younger age category of obesity onset was derived from a Cox model treating quintiles of weight trajectories/ group of obesity onset as a continuous variable, adjusted for the same variables as above. *The number at risk and number of events for obesity onset group were taken from imputed dataset 1 but varies slightly between imputed datasets. HR: hazard ratio, CI: confidence interval.

| Table S9. Hazard ratios (95% confidence intervals) of all-cause and cause-specific mortality in relation to per 5-unit BMI (kg/m^2^) at the last assessment, in men and women separately. | | | | | | |
| --- | --- | --- | --- | --- | --- | --- |
| **Cause of death** | | **Men (Total N=258,269)** | |  | **Women (Total N=361,784)** | |
|  |  | **N events** | **HR (95% CI)** |  | **N events** | **HR (95% CI)** |
| Main | All-cause | 86,673 | 1.18 (1.17–1.19) |  | 29,076 | 1.13 (1.11–1.14) |
|  | All-cause excluding respiratory disease & lung cancer | 76,601 | 1.24 (1.22–1.25) |  | 25,695 | 1.16 (1.14–1.17) |
|  | Cardiovascular disease | 33,682 | 1.34 (1.32–1.36) |  | 9,321 | 1.22 (1.19–1.25) |
|  | All cancer | 26,593 | 1.06 (1.04–1.09) |  | 9,977 | 1.08 (1.05–1.11) |
|  | Obesity-related cancer | 17,176 | 1.15 (1.12–1.17) |  | 6,237 | 1.14 (1.11–1.18) |
| Cardiovascular diseases | Hypertension | 1,140 | 1.57 (1.46–1.70) |  | 580 | 1.42 (1.32–1.52) |
|  | Ischemic heart disease | 17,692 | 1.37 (1.34–1.40) |  | 3,497 | 1.26 (1.22–1.31) |
|  | Atrial fibrillation/flutter | 1,358 | 1.41 (1.30–1.52) |  | 610 | 1.27 (1.17–1.38) |
|  | Heart failure | 2,191 | 1.41 (1.33–1.50) |  | 838 | 1.20 (1.11–1.31) |
|  | Cerebrovascular disease | 6,020 | 1.18 (1.14–1.23) |  | 2,169 | 1.08 (1.02–1.14) |
|  | Ischemic stroke | 1,521 | 1.28 (1.19–1.38) |  | 488 | 1.19 (1.07–1.32) |
|  | Haemorrhagic stroke | 1,278 | 1.19 (1.10–1.30) |  | 429 | 0.92 (0.80–1.04) |
|  | Aortic aneurysm | 1,035 | 1.16 (1.05–1.27) |  | - | - |
| Cancers | Pancreatic cancer | 1,827 | 1.16 (1.08–1.24) |  | 876 | 1.15 (1.06–1.24) |
|  | Stomach cancer | 1,087 | 1.14 (1.04–1.25) |  | 261 | 0.93 (0.79–1.09) |
|  | Colon cancer | 1,922 | 1.19 (1.11–1.27) |  | 852 | 1.13 (1.04–1.22) |
|  | Rectal cancer | 1,144 | 1.20 (1.10–1.32) |  | 319 | 1.10 (0.97–1.26) |
|  | Lymphoid neoplasms | 1,399 | 1.11 (1.02–1.20) |  | 387 | 1.24 (1.11–1.38) |
|  | Myeloid neoplasms | 753 | 1.01 (0.89–1.13) |  | 266 | 1.06 (0.92–1.24) |
|  | Multiple myeloma | 672 | 1.06 (0.94–1.20) |  | - | - |
|  | Oesophageal cancer | 694 | 1.07 (0.95–1.20) |  | - | - |
|  | Liver cancer | 800 | 1.74 (1.60–1.88) |  | - | - |
|  | Kidney cancer | 832 | 1.33 (1.21–1.47) |  | - | - |
|  | Bladder cancer | 957 | 0.91 (0.82–1.02) |  | - | - |
|  | Prostate cancer | 5,089 | 1.08 (1.03–1.13) |  | - | - |
|  | Female breast cancer | - | - |  | 1,497 | 1.13 (1.07–1.20) |
|  | Uterine cancer | - | - |  | 284 | 1.46 (1.32–1.62) |
|  | Ovarian cancer | - | - |  | 650 | 1.04 (0.94–1.14) |
| Other | Diabetes type 2 | 1,561 | 2.02 (1.94–2.12) |  | 484 | 1.66 (1.56–1.77) |
|  | Digestive disease | 2,584 | 1.34 (1.27–1.42) |  | 866 | 1.30 (1.22–1.40) |
|  | Genitourinary disease | 969 | 1.29 (1.18–1.42) |  | 259 | 1.53 (1.39–1.68) |
| Negative control | Brain cancer | 873 | 0.97 (0.87–1.09) |  | 331 | 0.87 (0.75–1.01) |
| Multivariable Cox regression with age as timescale was used to estimate the HRs and 95% CIs. The HRs were adjusted for highest attained education, country of birth, marital status at the last weight assessment, current smoking at the last weight assessment, mode of the last weight assessment, pregnancy status (only for women) at the last assessment, and stratified by birth decade. HR: hazard ratio, CI: confidence interval. | | | | | | |

|  | **Men** | | **Women** | |
| --- | --- | --- | --- | --- |
|  | **N events/N at risk** | **HR (95% CI)** | **N events/N at risk** | **HR (95% CI)** |
| Weight trajectories, ages 17–60 years |  |  |  |  |
| Quintile 1 | 185/51,654 | 1.00 (Reference) | 99/72,357 | 1.00 (Reference) |
| Quintile 2 | 202/51,654 | 1.08 (0.89–1.33) | 73/72,357 | 0.98 (0.72–1.33) |
| Quintile 3 | 168/51,654 | 0.95 (0.77–1.18) | 71/72,357 | 1.12 (0.82–1.54) |
| Quintile 4 | 170/51,654 | 1.00 (0.81–1.24) | 44/72,357 | 0.78 (0.54–1.13) |
| Quintile 5 | 148/51,653 | 0.94 (0.75–1.18) | 44/72,356 | 1.01 (0.70–1.47) |
| Per quintile | 873/258,269 | 0.98 (0.93–1.03) | 331/361,784 | 0.98 (0.90–1.07) |
| Age of obesity onset, years* |  |  |  |  |
| 17 to 29 | 10/4,885 | 0.59 (0.30–1.16) | 4/20,534 | 0.68 (0.23–2.00) |
| 30 to 44 | 37/13,570 | 0.86 (0.61–1.21) | 19/34,103 | 1.09 (0.66–1.82) |
| 45 to 60 | 91/30,695 | 0.93 (0.74–1.16) | 26/51,059 | 0.71 (0.47–1.08) |
| Never developing obesity by age 60 | 735/209,119 | 1.00 (Reference) | 282/256,088 | 1.00 (Reference) |
| Per younger age category | 873/258,269 | 0.90 (0.79–1.03) | 331/361,784 | 0.92 (0.74–1.14) |
| Per 0.5 kg/year weight changes at ages, years | |  |  |  |
| 17 to 29 | 873/258,269 | 0.92 (0.84–1.02) | 331/361,784 | 0.95 (0.81–1.10) |
| 30 to 44 |  | 0.98 (0.85–1.14) |  | 1.04 (0.78–1.40) |
| 45 to 60 |  | 0.99 (0.81–1.20) |  | 1.01 (0.67–1.53) |

# Table S10. Hazard ratios (95% confidence intervals) of mortality from brain cancer in relation to weight trajectories at ages 17–60 years, age of obesity onset, and weight changes in age periods in men and women separately.

The weight trajectories were estimated using a linear mixed-effects model of weight with natural cubic splines of age, and the individual weight change at ages 17–29, 30–44 and 45–60 years was the individual coefficient slope derived from a linear mixed-effects model of weight using linear splines of age at these periods. Other predictors in the linear mixed-effects model were the mode of weight measurement and pregnancy status at the time of weight assessment (for women). Individual BMI at any age within 17–60 years was calculated as predicted individual weight at each age (derived from linear mixed-effects model for weight trajectories) divided by measured height (kg/m^2^). Age at the first time with a predicted BMI ≥30 kg/m^2^ was treated as the age of obesity onset and used to classify into groups of obesity onset. Multivariable Cox regression with age as timescale was used to estimate the HRs and 95% CIs. The HRs were adjusted for predicted weight at age 17, height (except in the obesity onset analysis), highest attained education, birth country, marital status and current smoking at the last weight assessment, and stratified by birth decade. In the analysis for weight change in age periods, the age periods 30–44 and 45–60 additionally included adjustment for weight change in the previous age periods. The HR per higher quintile/younger age category of obesity onset was derived from a Cox model treating quintiles of weight trajectories/ group of obesity onset as a continuous variable, adjusted for the same variables as above. *The number at risk and number of events for obesity onset group were taken from imputed dataset 1 but varies slightly between imputed datasets. HR: hazard ratio, CI: confidence interval.

# Table S11. Hazard ratios (95% confidence intervals) and E-values for point estimates and lower confidence limits of hazard ratios of mortality associated with weight trajectory 5 vs weight trajectory 1.

|  |  | **Men** |  |  | **Women** |  |  |
| --- | --- | --- | --- | --- | --- | --- | --- |
|  | **Cause of death** | **HR (95% CI) Weight trajectory quintile 5 vs 1*** | **E-value for HR estimate** | **E-value for lower 95% confidence limit** | **HR (95% CI) Weight trajectory quintile 5 vs 1*** | **E-value for HR estimate** | **E-value for lower 95% confidence limit** |
| Main | All-cause | 1.36 (1.33–1.39) | 2.06 | 1.99 | 1.42 (1.37–1.48) | 2.20 | 2.07 |
|  | All-cause excl. respiratory disease & lung cancer | 1.45 (1.42–1.49) | 2.27 | 2.19 | 1.50 (1.44–1.57) | 2.37 | 2.23 |
|  | Cardiovascular disease | 1.71 (1.64–1.77) | 2.80 | 2.67 | 1.83 (1.69–1.97) | 3.05 | 2.78 |
|  | All cancer | 1.13 (1.08–1.17) | 1.50 | 1.37 | 1.23 (1.15–1.32) | 1.77 | 1.57 |
|  | Obesity-related cancer | 1.26 (1.19–1.33) | 1.83 | 1.68 | 1.37 (1.26–1.49) | 2.08 | 1.83 |
| Cardiovascular diseases | Hypertension | 2.42 (1.99–2.95) | 4.28 | 3.40 | 3.68 (2.80–4.84) | 6.83 | 5.05 |
|  | Ischemic heart disease | 1.74 (1.65–1.83) | 2.87 | 2.69 | 1.91 (1.69–2.16) | 3.24 | 2.78 |
|  | Atrial fibrillation/flutter | 1.77 (1.47–2.14) | 2.94 | 2.29 | 2.37 (1.76–3.18) | 4.17 | 2.92 |
|  | Heart failure | 1.94 (1.67–2.24) | 3.28 | 2.74 | 2.01 (1.52–2.67) | 3.44 | 2.41 |
|  | Cerebrovascular disease | 1.41 (1.28–1.54) | 2.17 | 1.89 | 1.35 (1.15–1.59) | 2.04 | 1.56 |
|  | Ischemic stroke | 1.44 (1.20–1.73) | 2.24 | 1.69 | 1.80 (1.31–2.48) | 3.01 | 1.95 |
|  | Haemorrhagic stroke | 1.42 (1.17–1.72) | 2.19 | 1.62 | 1.17 (0.85–1.62) | NA** | NA** |
|  | Aortic aneurysm | 1.48 (1.18–1.85) | 2.32 | 1.65 | - | - | - |
| Cancers | Oesophageal cancer | 1.19 (0.92–1.52) | NA** | NA** | - | - | - |
|  | Stomach cancer | 1.24 (1.01–1.50) | 1.78 | 1.14 | 0.76 (0.49–1.17) | NA** | NA** |
|  | Colon cancer | 1.36 (1.17–1.59) | 2.07 | 1.61 | 1.18 (0.93–1.50) | NA** | NA** |
|  | Rectal cancer | 1.36 (1.11–1.65) | 2.05 | 1.46 | 1.13 (0.76–1.67) | NA** | NA** |
|  | Liver cancer | 2.77 (2.23–3.45) | 4.99 | 3.88 | - | - | - |
|  | Pancreatic cancer | 1.24 (1.07–1.45) | 1.80 | 1.34 | 1.27 (1.00–1.60) | 1.85 | 1.06 |
|  | Prostate cancer | 1.18 (1.06–1.30) | 1.63 | 1.33 | - | - | - |
|  | Kidney cancer | 1.39 (1.09–1.77) | 2.12 | 1.39 | - | - | - |
|  | Bladder cancer | 0.89 (0.71–1.12) | NA** | NA** | - | - | - |
|  | Lymphoid neoplasms | 1.10 (0.92–1.32) | NA** | NA** | 1.34 (0.94–1.90) | NA** | NA** |
|  | Multiple myeloma | 1.06 (0.81–1.39) | NA** | NA** | - | - | - |
|  | Myeloid neoplasms | 1.11 (0.86–1.43) | NA** | NA** | 1.18 (0.77–1.81) | NA** | NA** |
|  | Breast | - | - | - | 1.72 (1.46–2.03) | 2.83 | 2.27 |
|  | Uterine | - | - | - | 3.10 (2.14–4.49) | 5.66 | 3.71 |
|  | Ovarian | - | - | - | 0.97 (0.74–1.29) | NA** | NA** |
| Other | Diabetes type 2 | 4.58 (3.89–5.40) | 8.63 | 7.24 | 5.79 (4.39–7.62) | 11.05 | 8.25 |
|  | Digestive disease | 1.79 (1.58–2.02) | 2.97 | 2.53 | 2.14 (1.71–2.68) | 3.71 | 2.82 |
|  | Genitourinary disease | 1.68 (1.35–2.11) | 2.76 | 2.03 | 2.97 (1.98–4.45) | 5.38 | 3.37 |

*The weight trajectories were estimated using a linear mixed-effects model of weight with natural cubic splines of age (four knots). Other predictors in the linear mixed-effects model were the mode of weight measurement (measured, self-reported, recalled) and pregnancy status at the time of weight assessment (yes, no). Multivariable Cox regression with age as timescale was used to estimate the HRs and 95% CIs. The HRs were adjusted for predicted weight at age 17, height, highest attained education, birth country, marital status andcurrent smoking at the last weight assessment, and stratified by birth decade.

**NA: There was no statistically significant association, therefore, we did not calculate the E-value for this.

| Table S12. Hazard ratios (95% confidence intervals) of primary mortality outcomes in relation to weight trajectories at ages 17–60, age of obesity onset, and weight change in age periods in men and women with five imputed data and complete data. | | | | | | | | | | |
| --- | --- | --- | --- | --- | --- | --- | --- | --- | --- | --- |
|  | **All causes** | | **All-cause excluding respiratory disease & lung cancer** | | **Cardiovascular disease** | | **All cancer** | | **Obesity-related cancer** | |
|  | **HR (95% CI)**  **5 imputed data** | **HR (95% CI)**  **Complete-case data** | **HR (95% CI)**  **5 imputed data** | **HR (95% CI)**  **Complete-case data** | **HR (95% CI)**  **5 imputed data** | **HR (95% CI)**  **Complete-case data** | **HR (95% CI)**  **5 imputed data** | **HR (95% CI)**  **Complete-case data** | **HR (95% CI)**  **5 imputed data** | **HR (95% CI)**  **Complete-case data** |
| **MEN (Total: Imputed data N=258,269; Complete-case data N=251,342)** | | |  |  |  |  |  |  |  |  |
| Weight trajectories, ages 17–60 years | | | | | | | | | | |
| Quintile 1 | Reference | Reference | Reference | Reference | Reference | Reference | Reference | Reference | Reference | Reference |
| Quintile 2 | 1.02 (1.00─1.04) | 1.02 (1.00-1.04) | 1.06 (1.04─1.08) | 1.06 (1.04-1.08) | 1.09 (1.06─1.12) | 1.09 (1.06-1.13) | 1.00 (0.97─1.04) | 1.01 (0.97-1.04) | 1.07 (1.02─1.11) | 1.07 (1.03-1.12) |
| Quintile 3 | 1.05 (1.02─1.07) | 1.05 (1.02-1.07) | 1.09 (1.07─1.12) | 1.09 (1.07-1.12) | 1.18 (1.14─1.22) | 1.19 (1.15-1.23) | 0.99 (0.96─1.03) | 0.99 (0.96-1.03) | 1.07 (1.03─1.12) | 1.07 (1.02-1.12) |
| Quintile 4 | 1.13 (1.11─1.16) | 1.13 (1.11-1.16) | 1.20 (1.17─1.23) | 1.20 (1.17-1.23) | 1.34 (1.29─1.38) | 1.33 (1.29-1.38) | 1.02 (0.98─1.06) | 1.03 (0.99-1.07) | 1.12 (1.06─1.17) | 1.13 (1.07-1.18) |
| Quintile 5 | 1.36 (1.33─1.39) | 1.35 (1.32-1.39) | 1.45 (1.42─1.49) | 1.45 (1.41-1.49) | 1.71 (1.64─1.77) | 1.70 (1.64-1.77) | 1.13 (1.08─1.17) | 1.13 (1.08-1.18) | 1.26 (1.19─1.33) | 1.26 (1.20-1.33) |
| Per quintile | 1.07 (1.06─1.07) | 1.07 (1.06-1.07) | 1.09 (1.08─1.09) | 1.09 (1.08-1.09) | 1.13 (1.12─1.14) | 1.13 (1.12-1.14) | 1.02 (1.01─1.03) | 1.02 (1.01-1.03) | 1.05 (1.04─1.06) | 1.05 (1.04-1.06) |
| Age of obesity onset, years | | | | | | | | | | |
| 17 to 29 | 1.69 (1.60─1.79) | 1.68 (1.58-1.78) | 1.71 (1.61─1.81) | 1.69 (1.59-1.80) | 2.03 (1.85─2.22) | 2.02 (1.84-2.21) | 1.16 (1.04─1.30) | 1.16 (1.03-1.30) | 1.29 (1.13─1.47) | 1.29 (1.12-1.47) |
| 30 to 44 | 1.48 (1.43─1.54) | 1.48 (1.43-1.53) | 1.55 (1.49─1.61) | 1.54 (1.49-1.60) | 1.83 (1.74─1.93) | 1.83 (1.73-1.93) | 1.12 (1.04─1.19) | 1.11 (1.04-1.19) | 1.25 (1.15─1.35) | 1.24 (1.14-1.35) |
| 45 to 60 | 1.25 (1.22─1.28) | 1.25 (1.22-1.28) | 1.29 (1.26─1.33) | 1.29 (1.26-1.32) | 1.48 (1.43─1.54) | 1.48 (1.42-1.53) | 1.06 (1.02─1.11) | 1.07 (1.02-1.12) | 1.10 (1.04─1.16) | 1.11 (1.05-1.17) |
| Never developing obesity by age 60 | Reference | Reference | Reference | Reference | Reference | Reference | Reference | Reference | Reference | Reference |
| Per younger age category | 1.22 (1.20─1.23) | 1.21 (1.20-1.23) | 1.24 (1.22─1.25) | 1.23 (1.22-1.25) | 1.35 (1.32─1.37) | 1.34 (1.32-1.37) | 1.06 (1.03─1.08) | 1.06 (1.03-1.08) | 1.10 (1.07─1.14) | 1.10 (1.07-1.14) |
| Per 0.5 kg/year weight changes at ages, years | | | | | | | | | | |
| 17 to 29 | 1.18 (1.17─1.19) | 1.18 (1.16-1.19) | 1.21 (1.20─1.23) | 1.21 (1.20-1.23) | 1.29 (1.27─1.31) | 1.29 (1.26-1.31) | 1.08 (1.06─1.10) | 1.08 (1.06-1.10) | 1.15 (1.12─1.18) | 1.15 (1.12-1.18) |
| 30 to 44 | 1.09 (1.08─1.11) | 1.09 (1.07-1.11) | 1.13 (1.11─1.15) | 1.13 (1.11-1.14) | 1.21 (1.18─1.24) | 1.21 (1.18-1.24) | 1.01 (0.98─1.03) | 1.01 (0.98-1.03) | 1.04 (1.00─1.07) | 1.04 (1.00-1.07) |
| 45 to 60 | 1.04 (1.02─1.06) | 1.04 (1.02-1.06) | 1.06 (1.04─1.08) | 1.06 (1.04-1.08) | 1.10 (1.08─1.13) | 1.10 (1.08-1.13) | 1.02 (0.99─1.05) | 1.02 (0.99-1.05) | 1.05 (1.01─1.09) | 1.05 (1.02-1.09) |
| **WOMEN (Total: Imputed data N=361,784; Complete-case data N=306,955)** | | |  |  |  |  |  |  |  |  |
| Weight trajectories, ages 17–60 years | | | | | | | | | | |
| Quintile 1 | Reference | Reference | Reference | Reference | Reference | Reference | Reference | Reference | Reference | Reference |
| Quintile 2 | 1.04 (1.01─1.08) | 1.05 (1.02-1.09) | 1.08 (1.04─1.11) | 1.09 (1.05-1.13) | 1.11 (1.05─1.18) | 1.13 (1.07-1.20) | 1.03 (0.97─1.09) | 1.03 (0.97-1.09) | 1.09 (1.02─1.17) | 1.09 (1.01-1.17) |
| Quintile 3 | 1.13 (1.09─1.17) | 1.14 (1.10-1.19) | 1.17 (1.13─1.22) | 1.19 (1.14-1.24) | 1.22 (1.14─1.30) | 1.23 (1.15-1.31) | 1.12 (1.05─1.18) | 1.13 (1.06-1.20) | 1.18 (1.10─1.28) | 1.20 (1.11-1.30) |
| Quintile 4 | 1.16 (1.12─1.21) | 1.17 (1.13-1.22) | 1.21 (1.16─1.26) | 1.22 (1.17-1.27) | 1.30 (1.22─1.39) | 1.28 (1.19-1.38) | 1.09 (1.03─1.16) | 1.12 (1.05-1.20) | 1.21 (1.12─1.31) | 1.23 (1.13-1.34) |
| Quintile 5 | 1.42 (1.37─1.48) | 1.45 (1.38-1.51) | 1.50 (1.44─1.57) | 1.53 (1.46-1.60) | 1.83 (1.69─1.97) | 1.87 (1.73-2.03) | 1.23 (1.15─1.32) | 1.22 (1.13-1.31) | 1.37 (1.26─1.49) | 1.36 (1.25-1.50) |
| Per quintile | 1.08 (1.07─1.09) | 1.08 (1.07-1.09) | 1.09 (1.08─1.10) | 1.09 (1.08-1.11) | 1.13 (1.11─1.15) | 1.13 (1.11-1.15) | 1.05 (1.03─1.06) | 1.05 (1.03-1.07) | 1.08 (1.06─1.10) | 1.08 (1.06-1.10) |
| Age of obesity onset, years |  |  |  |  |  |  |  |  |  |  |
| 17 to 29 | 1.71 (1.55─1.88) | 1.80 (1.62-2.00) | 1.75 (1.58─1.94) | 1.85 (1.65-2.06) | 2.26 (1.89─2.70) | 2.49 (2.05-3.01) | 1.06 (0.89─1.26) | 1.00 (0.82-1.22) | 1.15 (0.93─1.41) | 1.12 (0.88-1.42) |
| 30 to 44 | 1.48 (1.40─1.57) | 1.53 (1.44-1.63) | 1.54 (1.45─1.64) | 1.60 (1.50-1.71) | 2.00 (1.81─2.22) | 2.07 (1.86-2.31) | 1.16 (1.05─1.28) | 1.19 (1.07-1.32) | 1.22 (1.08─1.38) | 1.25 (1.10-1.43) |
| 45 to 60 | 1.24 (1.19─1.29) | 1.25 (1.20-1.30) | 1.26 (1.21─1.32) | 1.27 (1.22-1.33) | 1.42 (1.32─1.52) | 1.45 (1.34-1.56) | 1.13 (1.06─1.21) | 1.13 (1.05-1.21) | 1.21 (1.12─1.31) | 1.21 (1.11-1.32) |
| Never developing obesity by age 60 | Reference | Reference | Reference | Reference | Reference | Reference | Reference | Reference | Reference | Reference |
| Per younger age category | 1.21 (1.19─1.24) | 1.23 (1.21-1.26) | 1.23 (1.21─1.26) | 1.25 (1.22-1.29) | 1.38 (1.33─1.43) | 1.41 (1.36-1.47) | 1.07 (1.03─1.11) | 1.07 (1.03-1.11) | 1.11 (1.06─1.16) | 1.11 (1.06-1.17) |
| Per 0.5 kg/year weight changes at ages, years | | | | | | | | | | |
| 17 to 29 | 1.16 (1.14─1.18) | 1.16 (1.14-1.18) | 1.19 (1.17─1.21) | 1.19 (1.17-1.21) | 1.30 (1.27─1.34) | 1.30 (1.26-1.34) | 1.06 (1.03─1.09) | 1.07 (1.04-1.10) | 1.11 (1.07─1.15) | 1.11 (1.08-1.15) |
| 30 to 44 | 1.11 (1.08─1.14) | 1.12 (1.09-1.15) | 1.14 (1.10─1.17) | 1.15 (1.11-1.18) | 1.14 (1.09─1.20) | 1.16 (1.10-1.22) | 1.15 (1.09─1.20) | 1.14 (1.09-1.20) | 1.17 (1.11─1.25) | 1.17 (1.10-1.25) |
| 45 to 60 | 0.97 (0.94─1.01) | 0.98 (0.95-1.02) | 1.00 (0.97─1.04) | 1.02 (0.98-1.06) | 0.97 (0.92─1.03) | 1.00 (0.94-1.06) | 1.09 (1.03─1.16) | 1.10 (1.03-1.17) | 1.17 (1.08─1.26) | 1.18 (1.09-1.27) |

The weight trajectories were estimated using a linear mixed-effects model of weight with natural cubic splines of age, and the individual weight change at ages 17–29, 30–44 and 45–60 years was the individual coefficient slope derived from a linear mixed-effects model of weight using linear splines of age at these periods. Other predictors in the linear mixed-effects model were the mode of weight measurement and pregnancy status at the time of weight assessment (for women). Individual BMI at any age within 17–60 years was calculated as predicted individual weight at each age (derived from linear mixed-effects model for weight trajectories) divided by measured height (kg/m^2^). Age at the first time with a predicted BMI ≥30 kg/m^2^ was treated as the age of obesity onset and used to classify into groups of obesity onset. Multivariable Cox regression with age as timescale was used to estimate the HRs and 95% CIs. The HRs were adjusted for predicted weight at age 17, height (except in the obesity onset analysis), highest attained education, birth country, marital status and current smoking at the last weight assessment, and stratified by birth decade. In the analysis for weight changes in age periods, the age periods 30–44 and 45–60 additionally included adjustment for weight change in the previous age periods. The HR per higher quintile/younger age category of obesity onset was derived from a Cox model treating quintiles of weight trajectories/ group of obesity onset as a continuous variable, adjusted for the same variables as above. HR: hazard ratio, CI: confidence interval.

# References

1. Bhaskaran K, Dos-Santos-Silva I, Leon DA, Douglas IJ, Smeeth L. Association of BMI with overall and cause-specific mortality: a population-based cohort study of 3·6 million adults in the UK. *Lancet Diabetes Endocrinol* 2018; **6**(12): 944–53.

2. Jochems SHJ, Stattin P, Haggstrom C, et al. Height, body mass index and prostate cancer risk and mortality by way of detection and cancer risk category. *Int J Cancer* 2020; **147**(12): 3328–38.

3. Kim MS, Kim WJ, Khera AV, et al. Association between adiposity and cardiovascular outcomes: an umbrella review and meta-analysis of observational and Mendelian randomization studies. *Eur Heart J* 2021; **42**(34): 3388–403.

4. Elhakeem A, Hughes RA, Tilling K, et al. Using linear and natural cubic splines, SITAR, and latent trajectory models to characterise nonlinear longitudinal growth trajectories in cohort studies. *BMC Med Res Methodol* 2022; **22**(1): 68.

5. White IR, Royston P, Wood AM. Multiple imputation using chained equations: Issues and guidance for practice. *Stat Med* 2011; **30**(4): 377–99.

6. White IR, Royston P. Imputing missing covariate values for the Cox model. *Stat Med* 2009; **28**(15): 1982–98.

7. Lipsitch M, Tchetgen Tchetgen E, Cohen T. Negative controls: a tool for detecting confounding and bias in observational studies. *Epidemiology* 2010; **21**(3): 383–8.
